# A synthesis strategy for tetracyclic terpenoids leads to agonists of ERβ

**DOI:** 10.1038/s41467-019-10415-6

**Published:** 2019-06-04

**Authors:** Wan Shin Kim, Zachary A. Shalit, Sidney M. Nguyen, Emmalie Schoepke, Alan Eastman, Thomas P. Burris, Arti B. Gaur, Glenn C. Micalizio

**Affiliations:** 1 Dartmouth College, Department of Chemistry, Burke Laboratory, Hanover, NH 03755 USA; 2 Dartmouth College, Geisel School of Medicine, Department of Neurology, Lebanon, NH 03756 USA; 3 Center for Clinical Pharmacology, Washington University School of Medicine and St. Louis College of Pharmacy, St. Louis, MO 63110 USA; 4 Dartmouth College, Geisel School of Medicine, Department of Molecular and Systems Biology, Lebanon, NH 03756 USA

**Keywords:** Natural product synthesis, Natural product synthesis

## Abstract

Natural product and natural product-like molecules continue to be important for the development of pharmaceutical agents, as molecules in this class play a vital role in the pipeline for new therapeutics. Among these, tetracyclic terpenoids are privileged, with >100 being FDA-approved drugs. Despite this significant pharmaceutical success, there remain considerable limitations to broad medicinal exploitation of the class due to lingering scientific challenges associated with compound availability. Here, we report a concise asymmetric route to forging natural and unnatural (enantiomeric) C19 and C20 tetracyclic terpenoid skeletons suitable to drive medicinal exploration. While efforts have been focused on establishing the chemical science, early investigations reveal that the emerging chemical technology can deliver compositions of matter that are potent and selective agonists of the estrogen receptor beta, and that are selectively cytotoxic in two different glioblastoma cell lines (U251 and U87).

## Abstract (web summary)

Many natural-product like drugs have a tetracyclic terpenoid core. Here, the authors developed a synthesis of triterpene-like tetracyclic systems, and apply this method to the preparation of a number of enantiomeric compounds, two of which are very selective ligands for estrogen receptor beta

## Introduction

The means by which organic molecules can be synthesized has a profound impact on medicinal chemistry and drug discovery. Simply stated, without efficient and flexible processes capable of preparing compositions of matter with drug-like properties, medicinal chemistry programs cannot easily materialize. While it is well appreciated that natural products are a rich source of leads for drug discovery, it is also understood that their molecular architectures are typically quite challenging to address with de novo synthesis. In fact, organic chemistry to enable broad investigation of structure–activity relationships associated with medicinally relevant natural products is often not available. This reality is one of the many factors that continue to fuel developments in the field of chemical synthesis, where attention is directed at providing de novo means of producing natural product and natural product-inspired agents with increased efficiency and molecular flexibility.

**Fig. 1:**
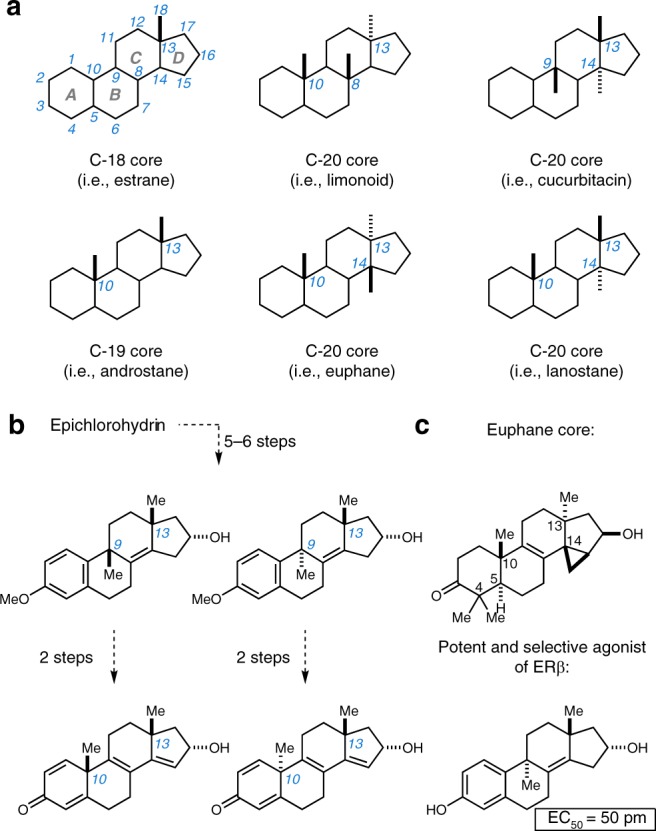
Tetracyclic terpenoid skeletons and a means of synthesis from epichlorohydrin. **a** The general skeleton of tetracyclic terpenoids (C18 through C20). **b** A unified enantiospecific route to a variety of tetracyclic terpenoid motifs. **c** Application to the synthesis of a euphane-based tetracyclic skeleton and the synthesis and discovery of a potent and selective agonist of the estrogen receptor beta (ERβ)

Of the vast classes of natural products known to possess important biological and medicinal properties, perhaps none is more proven as a source of therapeutics than tetracyclic terpenoids. This large class of molecules, including steroids and cardiac glycosides, has had a transformative impact on our society (Fig. 1a; C-18 and C-19 cores)^1–6^. Today, scores of such agents are FDA-approved drugs used to treat a wide range of conditions, and this success supports the conclusion that the basic molecular skeleton associated with the class is a validated platform for drug discovery and development.

Given this standing in medicinal chemistry, it is perhaps surprising that tetracyclic terpenoids persist as significant challenges for de novo synthesis. While numerous achievements have described synthetic forging of members of the broad class^7–13^, and the scientific advances central to such triumphs in natural product total synthesis have had a profound impact on the evolution of organic chemistry as a discipline, it remains the case that investigation of structure–activity relationships and production of active pharmaceutical ingredients (APIs) based on tetracyclic terpenoids typically proceed by derivatization of readily available natural products (a synthesis activity otherwise known as semisynthesis)^12^.

This reality greatly constrains efforts aiming to investigate the medicinal potential of diverse compositions of matter inspired by this natural product class, and has likely contributed to the fact that 100% of FDA-approved drugs in the area have the natural absolute stereochemistry (the mirror image isomer observed in nature). Accepting that the drug-like physical properties of a molecule are not strictly dependent on absolute stereochemistry, it appears as though a vast region of pharmaceutically relevant chemical space has remained underexplored for decades^14–17^.

In nature, tetracyclic terpenoids possess a wide variety of molecular skeletons that differ dramatically from the common enantiodefined C18 and C19 core skeletons of well-studied steroid hormones (Fig. 1a). In fact, many subclasses of natural products in this area that have not been thoroughly investigated in biology and medicine possess additional quaternary centers at C8, C9, and C14, and display unique relative stereochemistry with respect to each quaternary center.

Here, as summarized in Fig. 1b, c, a unified asymmetric entry to tetracyclic systems related to the large and diverse class of tetracyclic terpenoids is described. This chemical technology is shown to be capable of accessing the euphane tetracyclic system, while also delivering potent and selective agonists of the estrogen receptor beta (ERβ).

## Results

### Design of the synthetic route

**Fig. 2:**
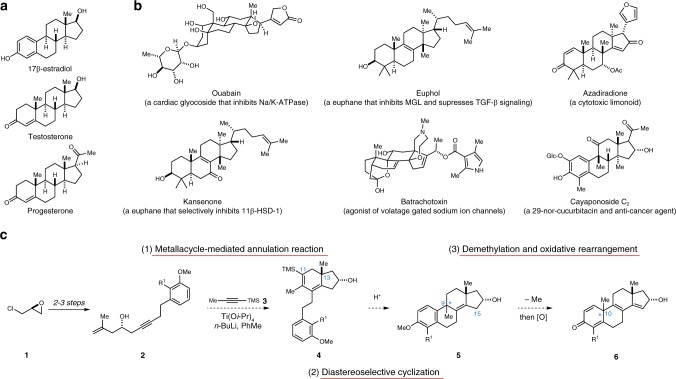
Design of a synthesis strategy to access varied tetracyclic terpenoid skeletons. **a** Examples of steroid hormones including an estrane, an androstane, and a pregnane. **b** Complex tetracyclic terpenoids that present distinct challenges for chemical synthesis in comparison with relatively simple steroid hormones. **c** Design of a concise approach to the enantiospecific assembly of C19 tetracyclic terpenoid core skeletons **5** and **6**

Studies began with recognition that the challenges associated with enantioselective synthesis of steroid hormones are distinct from those associated with highly oxygenated and densely functionalized tetracyclic terpenoids that possess widely varying substitution and stereochemistries (Fig. 2a, b). Hoping to establish a unified theme capable of addressing the key molecular challenges associated with a variety of tetracyclic terpenoid skeletons (i.e., addressing the location and relative stereochemistry of characteristic quaternary carbon centers), the synthetic strategy depicted in Fig. 2c was conceived. Taking a departure from previous studies aimed at the enantiospecific synthesis of estranes^18^, it was thought that metallacycle-mediated annulative cross-coupling^19^ between a readily available enyne (**2**) and trimethylsilylpropyne **3** would deliver hydrindane intermediate **4**, possessing the C13 quaternary center common to this large natural product class. Notably, and consistent with previous studies, it was anticipated that **4** would be formed with high levels of regioselectivity, positioning the trimethylsilyl (TMS) group at C11 of the carbocyclic nucleus.

With hydrindane **4** in hand, it was thought possible to introduce a C9 quaternary center simply by engaging the vinylsilane moiety in initial acid-mediated protodesilylation, followed by a second regioselective protonation of the diene to deliver a transient fully substituted allylic cation intermediate (not depicted). Intramolecular regio- and stereoselective Friedel–Crafts alkylation was then envisioned as a means to establish the fused tetracyclic skeleton **5**—a molecular composition that has structural features reminiscent of numerous natural products (i.e., cayaponoside C_2_, Fig. 2b). Rather than serving as a terminal intermediate of relevance for a relatively small collection of terpenoid targets harboring a C9 quaternary center, it was thought that this intermediate may also serve as a platform to access numerous other terpenoid skeletons. Specifically, a sequence consisting of A-ring methyl ether cleavage followed by a unique oxidative rearrangement marked by a 1,2-alkyl shift from C9 to C10 was imagined to pave a path from **5** to the tetracyclic system **6**. Notably, this tetraene-containing product contains the C10/C13 quaternary centers seen in numerous steroid hormones, cardiac glycosides, limonoids, euphanes, and lanostanes (among others).

While oxidative alkyl shift reactions have been described as a means to convert phenols to dienones possessing a quaternary center^20–22^, these reactions are typically conducted with substrates that enable C–C bond formation through a semi-pinacol-like process (substrates containing a benzylic tertiary alcohol), where termination of the cationic rearrangement is coupled to the formation of a ketone. The proposed process for conversion of **5** to **6** in Fig. 2c calls for migration of a methyl group from a quaternary center, generation of a tertiary allylic cation, and selective loss of a proton from C15 of the D-ring. Besides not having strong precedent in support of this proposed molecular transformation, at the outset of these studies methyl migration in oxidative dearomatization chemistry was appreciated as being notoriously problematic^21,22^.

### Asymmetric synthesis of tetracyclic terpenoid skeletons

**Fig. 3:**
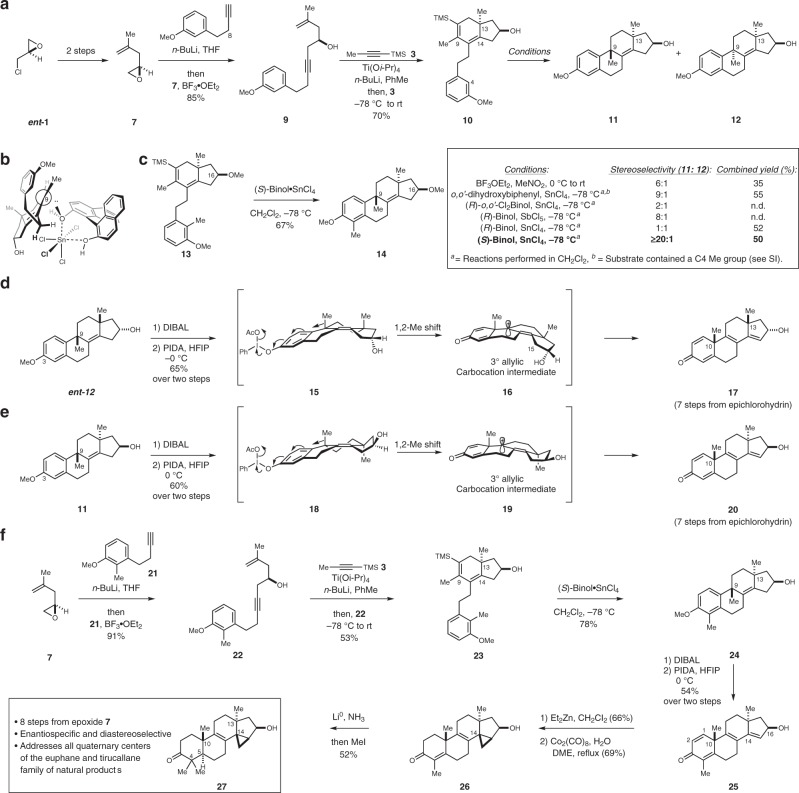
Establishment of an asymmetric synthesis of tetracyclic terpenoid skeletons from epichlorohydrin. **a** Establishing a synthetic route from epichlorohydrin to steroidal tetracycles bearing a quaternary center at C9. **b** A proposed orientation of (*S*)-Binol•SnCl_4_ in the site- and stereoselective cyclization reaction. **c** Demonstration that the C16 (D-ring) alcohol is not important for stereocontrol in the acid-mediated cyclization reaction. **d** Development of an oxidative shift process for the generation of a steroidal tetracycle. **e** Application of this strategy to access an isomeric skeleton of relevance for euphane and tirucallane natural products. **f** Combining an oxidative alkyl shift with an alkoxide-directed Simmons–Smith cyclopropanation in a sequence that forges additional quaternary centers at C4, C10, and C14

As illustrated in Fig. 3a, epichlorohydrin (*ent-***1**) was converted to epoxide **7** by an established two-step procedure^23^, and subsequent BF_3_•OEt_2_-promoted addition of an acetylide derived from **8** provided the stereodefined substrate **9** in 85% yield. Regio- and stereoselective annulative cross-coupling with TMS-propyne **3** then delivered hydrindane **10** in 70% yield as a single regio- and stereoisomer^19^. Next, conditions were explored to accomplish the desired tandem protodesilylation and cyclization reaction to form the C9–C10 bond and establish the C9 quaternary center. As illustrated in the highlighted table, treatment of **10** with BF_3_•OEt_2_ in nitromethane at 0 °C, converted the hydrindane intermediate to the fully functionalized tetracycles **11** and **12**. Here, cyclization appeared to occur only at C9 of the intermediate allylic cation (rather than at C14), with moderate selectivity for production of the *anti*-isomer **11** (dr = 6:1; the major product possesses a quaternary center at C9 that projects the methyl group on the opposite face of the tetracycle as the preixisting group at C13).

In an effort to both enhance diastereoselection for this cyclization and to alter the inherent facial preference for the process, chiral Brønstead acids derived from complexation of Binol to a Lewis acid were explored. This strategy was introduced by Professor Yamamoto and further studied by Professor Corey as a means to achieve enantioselective polyene cyclization reactions^24,25^, and has to our knowledge not been investigated for related double-asymmetric Friedel–Crafts type cyclization chemistry. To establish a baseline for understanding simple diastereoselection in this double-asymmetric annulation process, an achiral system based on the combination of *o,o’*-dihydroxybiphenyl•SnCl_4_ in CH_2_Cl_2_ at −78 °C was first studied. Notably, the selectivity for formation of the *anti*-isomer in this cyclization reaction was increased to 9:1. Next, use of enantiodefined Binols in complex with SnCl_4_ or SbCl_5_ led to the discovery of a clear double asymmetric relationship. In the mismatched case, use of (*R*)-Binol•SbCl_5_ delivered the cyclized products as an 8:1 mixture of *anti*-(**11**) and *syn*-(**12**) stereoisomers,  and use of (*R*)-Binol•SnCl_4_ resulted in production of the *syn*-isomer in approximately an equal ratio with the *anti*-isomer (52% combined yield).

In contrast, the matched case proved to be highly stereoselective. Treatment of **10** with (*S*)-Binol•SnCl_4_ delivered the *anti*-isomer **11** in 50% yield with ≥ 20:1 stereoselectivity. Building on the empirical model first offered by Yamamoto^24^ for enantioselective Brønstead acid-mediated polyene cyclization, it is proposed that the relevant Binol•SnCl_4_ complex promotes cyclization of substrates like **10** through an arrangement similar to that depicted in Fig. 3b. While the factors that contribute to controlling selectivity in this reaction will be the subject of ongoing investigations, at this early stage of development it has been established that the D-ring free hydroxy group (at C16) is not an essential feature for stereoselection in this unique double-asymmetric Brønstead acid-mediated ring-closing process. As illustrated in Fig. 3c, the protected substrate **13** (C16 methyl ether) can be smoothly converted to a stereodefined tetracycle (**14**) in 67% yield, and with similarly high levels of stereocontrol (ds ≥ 20:1).

Moving forward, a concise sequence of steps was realized to transform each of the products of double-asymmetric Friedel–Crafts cyclization to tetracyclic C19 skeletons typical of steroid hormones. As depicted in Fig. 3d, reductive demethylation of the enantiodefined *syn*-isomer ***ent-*12** (prepared from ***ent*-10**) with diisobutylaluminum hydride delivered the corresponding phenol in high yield^26^. The designed oxidative rearrangement was then found to proceed smoothly by treatment with (diacetoxyiodo)benzene (also referred to as phenyliodine(III) diacetate, or PIDA)^20–22^. Presumably acting by way of intermediate **15**, stereospecific 1,2-methyl shift from C9 to C10 is thought to deliver a highly stabilized tertiary allylic carbocation **16** that is terminated by selective loss of a proton from C15, leading to production of the stereodefined dienone **17**—a tetracyclic product that houses the quaternary centers at C10 and C13 that are seen in scores of tetracyclic terpenoid targets known to possess an array of medicinally relevant properties. Notably, the basic strategy to access **17** is equally effective for preparing the stereoisomeric tetracycle **20** (Fig. 3e); a compound that possesses the stereochemical features at C10 and C13 that are common in limonoids and euphane and tirucallane natural products.

As illustrated in Fig. 3f, this reactivity proved effective for establishing molecular features that are prominent in the family of tetracyclic triterpenoids exemplified in Fig. 2a (i.e., euphol and kansenone). After coupling epoxide **7** to alkyne **21**, the resulting enyne (**22**) was transformed to hydrindane **23** by alkoxide-directed metallacycle-mediated annulative cross-coupling. Next, the double-asymmetric Brønstead acid-promoted cyclization process proved highly effective for coversion of **23** to the tetracycle **24** (78% yield; ds ≥ 25:1), and subsequent demethylation and oxidative rearrangement delivered the tetraene **25** as a single isomer. Installation of the additional quaternary center at C14 was then accomplished by capitalizing on the stereochemistry at C16 that was previously employed to both enable and control the regio- and stereochemical course of the metallacycle-mediated annulation reaction. While a number of strategies may be considered to achieve such a functionalization process (i.e., [2,3] or [3,3]-sigmatropic rearrangement chemistry), hydroxyl-directed Simmons–Smith cyclopropanation^27^ was selected for study here. Gratifyingly, this approach proved highly effective at establishing the *trans*-fused hydrindane system featuring adjacent quaternary centers at C13 and C14. Subsequent treatment with Co_2_(CO)_8_ and H_2_O resulted in site-selective A-ring enone reduction^28^, and furnished the stereodefined intermediate **26** in 69% yield. Finally, a one-pot Birch reduction and alkylation delivered the fully functionalized and differentiated product **27** that possesses the typical quaternary carbons at C4, C10, C13, and C14 observed in euphane natural products in 52% yield as a single stereoisomer.

While further synthetic manipulation of intermediates like **27** was not of interest in this study, the chemical transformations depicted in Fig. 3 realize a common synthetic pathway for enantiospecific access to tetracyclic terpenoid skeletons possessing a variety of stereochemical relationships, as well as spatially distinct patterns of fully substituted sp^3^ carbons (quaternary centers). Furthermore, the highly unsaturated nature of many of the key intermediates provide opportunities to exploit types of functionalization chemistry not typically encountered in other synthetic approaches to such tetracycle formation (i.e., intermediates accessible through biomimetic cation–olefin cyclization). As such, it is anticipate that this suite of chemistry has the potential to offer convenient access to synthetic agents in the broader class that are not easily prepared through other strategies for de novo synthesis, and that are even wholly incompatible with efforts that embrace semisynthesis (functionalization of readily available natural tetracyclic terpenoid starting materials).

### Discovery of estrogen receptor beta (ERβ) agonists

With recognition that the chemical pathway established proceeds by way of structurally unique partially aromatic tetracycles in the estrane series (i.e., **11** and **12**; Fig. 3a), attention was directed toward investigating the potential value of these species outside of their future role as synthetic intermediates in natural product and function-oriented synthesis campaigns. In fact, relatively few natural tetracyclic terpenoids have the core structure seen in the intermediates accessed here (after double-asymmetric Friedel–Crafts cyclization), and none have the combination of substitution and unsaturation seen in **11** and **12**. That said, it was appreciated that partially aromatic synthetic steroidal systems have been described as modulators of estrogen receptors, ERα and ERβ (vide infra).

In particular, the estrogen receptor beta (ERβ) is a nuclear hormone receptor that was discovered in 1996 and has been recognized as a potentially important pharmaceutical target^29,30^. This nuclear hormone receptor is distinct from ERα; it is not expressed in the pituitary and has little role in the proliferation of the mammary gland or endometrium^30^. Agonism of ERβ is of great current pharmaceutical interest due to a variety of observations that include decreasing survival of p53-defective cancer cells by imparing G_2_/M checkpoint signaling^31^, playing a role as a tumor suppressor of epithelial ovarian cancer^32^ and of gliomas^33^, reducing invasiveness of triple-negative breast cancer cells^34^, inducing cell death in acute myeloid leukemia^35^, and decreasing proliferation of ovarian cancer cells^36^. Notably, ERβ has also been considered as a target of potential value for neuroprotection^37^, diabetes^38^, inflammation and pain^39–42^.

Being aware that 9α-substituted estratrienes have been described as selectively active estrogens^43^, we sought to investigate the ER-modulating properties associated with some molecules now readily available from the chemical advances described herein. Notably, unlike most explorations into the bioactivity of steroidal systems, the synthetic chemistry established here provides straightforward access to both enantiomeric series, and delivers unique compositions of matter whose additional unsaturation at C8 offers unique physical properties (i.e., altered solubility and rigidity) along with subtle perturbation of three dimensional molecular structure in comparison to related saturated variants.

**Fig. 4:**
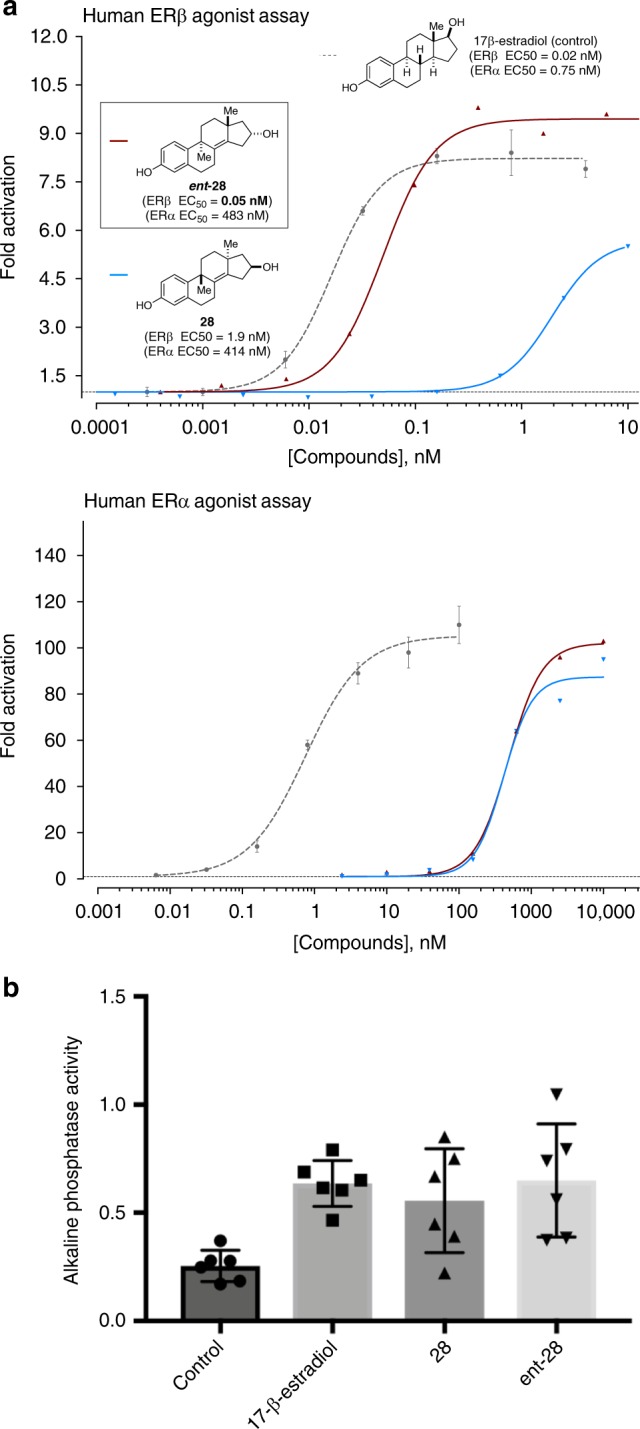
**28** and ***ent***-**28** are selective agonists of ERβ. **a** ERβ and ERα agonist assays (Z’ = 0.67 for ERβ Z’ = 0.77 for ERα, see Supplementary Information for experimental details). **b 28** and ***ent*-28** are agonists of ERβ in human DU-145 prostate cancer cells (17β-estradiol, **28**, and ***ent*-28** were evaluated at 5 µM). Error bars represent standard deviation of the mean (*n* = 6 per group), *y*-axis in OD 405 nM. Source data are provided as a Source Data File

As illustrated in Fig. 4a, the enantiomeric tetracycles **28** and ***ent*-28**, each prepared in only six steps from epichlorohydrin (as previously presented in Fig. 3a), were found to be potent and selective agonists of ERβ in an in vitro, bioluminescence cellular-reporter assay. Here, 17β-estradiol was used as a control, as it is appreciated to be a potent agonist of both ERα and ERβ. The enantiomer of interest for the de novo synthesis of euphane- and tirucallane-based natural products (**28**) was found to induce partial agonism of ERβ with an EC_50_ of 1.9 nM and clear selectivity for ERβ vs. ERα in comparison with 17β-estradiol. Speaking to the great impact that absolute stereochemistry can have on the biological and/or medicinal properties of organic molecules, the enantiomeric tetracycle (***ent*-28**) proved to be markedly more potent and selective than its antipode — it was found to be a highly potent full agonist of ERβ with an EC_50_ value of 0.05 nM, and a superior selectivity profile over ERα. To our knowledge, these novel compositions of matter have unprecedented properties as selective agonists of ERβ^33,36,37,41,42,44–49^, while offering a validated and pharmaceutically relevant backbone on which to build selective clinically valuable agents.

### Regulation of ERβ in prostate cancer cells

Prostatic epitheleal hyperplasia is evident in mice lacking ERβ^50^, suggesting that ERβ may play a role in limiting proliferation in the prostate. These data suggest that pharmacological activation of ERβ may be useful in treatment of prostate cancer. The DU-145 prostate cancer cell line only expresses ERβ^51^, and can be used as a model to examine the activation of ERβ in the absence of any potential confounding signal driven by ERα. Alkaline phosphatase (ALP) expression is induced in an ER-dependent manner, and has been used to characterize the activity of synthetic ER agonists and antagonists^52^.

In order to examine the ability of **28** and ***ent*-28** to activate ERβ in the context of a cell line expressing endogenous ERβ, DU-145 cells were treated for 24 h with either 17β-estradiol, **28**, or ***ent*-28**, and ALP activity derived from the cells was examined. As shown in Fig. 4b, 17β-estradiol was effective in inducing ALP activity as expected. In addition, both **28** and ***ent*-28** were effective at inducing ALP to levels comparable to 17β-estradiol. These data are consistent with **28** and ***ent*-28** functioning as ERβ agonists.

### Inhibition of glioblastoma in vitro

Glioblastomas are the most malignant and lethal brain tumors, with a median survival rate of 14 months and a 5-year survival rate of less than 10%. Despite attempts to combine surgery, radiation, and chemotherapeutics, gliomas recur in more than 90% of cases. This is primarily due to gliomas being heterogeneous tumors with each individual tumor expressing distinct patterns of oncogenes and mutated tumor-suppressor genes, thus making molecular characterization and targeted treatment challenging. However, elevated levels of ERβ expression have been observed in most glioblastomas examined to date^33,53^. ERβ is an established tumor suppressor in several cancers; higher expression of ERβ is correlated to a better prognosis, and ERβ agonists induce apoptosis^53–55^. Nevertheless, despite the tumor suppressive role of ERβ, 17β-estradiol (a potent but unselective agonist of ERα and ERβ) is not used as a therapy against gliomas because long-term treatment can result in cancers of the female reproductive system and prostate cancer in men^56,57^. In comparison with 17β-estradiol, **28** and ***ent*-28**^58^ have a unique profile for selective agonism of ERβ (Fig. 4a). Furthermore, as data from both the ERβ specific in vitro bioluminescence reporter assay (Fig. 4a) and the ERβ only expressing prostate cancer line DU-145 (Fig. 4b) established that **28** and ***ent*-28** are ERβ agonists, we hypothesized that targeting ERβ with **28** or ***ent*-28** in gliomas may establish a novel and highly efficacious therapy against this deadly brain tumor.

**Fig. 5:**
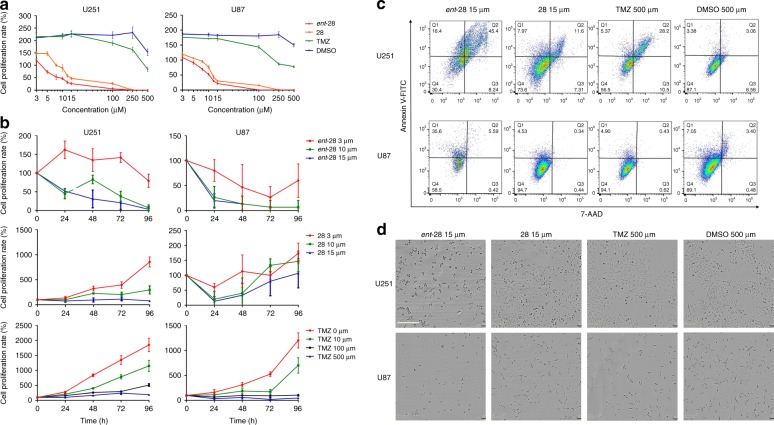
***Ent***-**28** and **28** inhibit proliferation and induce apoptosis in glioblastoma cell lines. **a** IC_50_ at 24 h: dose-dependent growth inhibition of U251 and U87 cells treated with **28**, ***ent*-28**, TMZ, and vehicle control DMSO (*n* = 4). **b** Cell proliferation over 96 h: viable cell counts of U251 and U87 cells treated with **28**, ***ent*-28**, TMZ, and vehicle control DMSO determined every 24 h for 96 h (*n* = 4). **c** Apoptosis at 24 h: flow-cytometric analysis of U251 and U87 co-stained with Annexin V-FITC/7-AAD after treatment with **28**, ***ent*-28**, TMZ, and vehicle control DMSO (*n* = 4). **d** Morphological analyses at 24 h: phase-contrast images using Incucyte for live cell tracking of U251 and U87 cells in response to treatment with **28**, ***ent*-28**, TMZ, and vehicle control DMSO. Live cells were tracked and imaged for 96 h (data not shown). Scale bar = 300 μm. All data in this figure are representative of four independent experiments, and four replicates of each treatment condition were analyzed per experiment. Mean ± s.d. of quadruplicates from one experiment are presented on the line plots. Source data are provided as a Source Data File

To determine the biological effect of **28** and ***ent-*28**, human glioblastoma-derived cell lines U251 and U87 were treated with varying concentrations of **28**, ***ent*-28**, temazolomide (TMZ; a standard of care chemotherapeutic agent), or vehicle-toxicity control, dimethylsulfoxide (DMSO) used at final volumes that were identical to those used to attain all concentrations tested of **28** and ***ent-*28**. Remarkably, **28** and ***ent*-28** demonstrated inhibition of glioma cell growth (IC_50_) at a 50-fold lower concentration when compared with TMZ after 24 h (Fig. 5a). Furthermore, when cell viability was assessed every 24h for 5 days, we observed that **28** and ***ent*-28** reduced viability and the resulting proliferation of glioblastoma lines in a dose-dependent manner compared with cells that were incubated with TMZ or DMSO (Fig. 5a; Supplementary Fig. 1). More importantly, cell viability of human neural stem cells as well as human astrocytes was not affected at the equivalent concentration, demonstrating that both **28** and ***ent*-28** have glioma-specific cytotoxic activity (Fig. 5; Supplementary Figs. 2, 3). This was further confirmed by MTT cell-viability assays wherein **28** and ***ent*-28** treatment significantly reduced the growth of glioblastoma cell lines in a dose-dependent manner (Supplementary Figs. 4, 5).

In order to elucidate the mechanism of this cell death, glioblastoma cells treated for 24 h with **28**, ***ent*-28**, TMZ, or DMSO control were analyzed for early- and late-apoptosis markers (Annexin V and 7-Aminoactinomycin D) by FACS analyses. These experiments revealed a sevenfold higher induction of apoptosis in U251 and U87 cells treated with **28** or ***ent*-28** compared with cells treated with either TMZ or DMSO controls (Fig. 5c; Supplementary Fig. 6). In addition, over a period of 5 days, morphological changes in U251 and U87 cells treated with **28** and ***ent*-28** were tracked using the Incucyte live cell imaging system. Increased loss of adherence and cellular integrity were observed following treatment of U251 and U87 with **28** and ***ent*-28**, correlating with the decreased viability and increased cell death observed previously; similar behavior was not seen with human neural stem cells as well as human astrocytes (Fig. 5d; Supplementary Figs. 2, 3).

Collectively, these investigations indicate that **28** and ***ent*-28** each reduce cell viability, decrease survival, and induce apoptosis selectively in glioblastoma cells. This profile supports further investigation of the novel enantiomeric tetracycles **28** and ***ent*-28** as therapeutic agents against gliomas that are resistant to all currently available treatment modalities.

In conclusion, the rich and diverse collection of tetracyclic terpenoids has served as motivation for the design and execution of a new chemical synthesis platform capable of constructing central motifs of their carbocyclic backbones in just a handful of steps from an inexpensive and readily available chiral starting material (epichlorohydrin). The process comprises a modern metallacycle-mediated annulative cross-coupling, a C9–C10 bond-forming process through a double-asymmetric Friedel–Crafts cyclization, and an oxidative rearrangement reaction. It has been shown that this sequence of molecular transformations can be used to deliver a variety of stereodefined systems that contain quaternary centers at C9, C10, C13, and C14, and that compositions of matter encountered in this chemical synthesis pathway are potent agonists of ERβ and have selective anticancer properties against glioblastoma. While it is anticipated that molecules now accessible from this chemical advance will be a rich source of bioactive leads for drug discovery and development across a diverse therapeutic landscape, it is already clear that the science described allows for de novo synthesis of either enantiomer of the tetracyclic skeletons resident in a wide variety of terpenoids with ease, rendering unique opportunities for medicinal exploration viable. Efforts directed at advancing this collection of advances in preparative organic chemistry to address challenges in natural product synthesis and medicinal science are of great current and future interest.

## Methods

### Synthesis of enyne 22

To a stirring solution of **21** (5.4 g, 31 mmol, 2.0 equiv) in 90 mL of THF at −78 °C under N_2_ atmosphere was added *n*-BuLi (2.5 M in hexanes, 10 mL, 25 mmol, 1.6 equiv) dropwise over 3 min, and the resulting mixture was stirred for 30 min at the same temperature. After the specified period of time, BF_3_•OEt_2_ (4.0 g, 28 mmol, 1.8 equiv) was added dropwise to the reaction mixture followed by **7** (1.5 g, 15 mmol, 1.0 equiv). The resulting mixture was stirred at −78 °C under N_2_ atmosphere for 55 min, and then quenched with 50 mL of saturated solution of NaHCO_3_ at the same temperature. The resulting biphasic solution was warmed to room temperature, and then further diluted with 50 mL of ethyl acetate. The organic layer was separated, dried over Na_2_SO_4_, filtered, and then the filtrate was concentrated in vacuo. SiO_2_ flash column chromatography afforded the title compound **22** as a clear oil (3.9 g, 91%); Spectral Data for **22**: ^1^H NMR (600 MHz, Chloroform-*d*): δ 7.13 (t, *J* = 7.9 Hz, 1H), 6.85 (d, *J* = 1.1 Hz, 1H), 6.76 (d, *J* = 1.1 Hz, 1H), 4.92 – 4.86 (m, 1H), 4.86 – 4.80 (m, 1H), 3.89 – 3.84 (m, 1H), 3.83 (s, 3H), 2.87 (t, *J* = 7.7 Hz, 2H), 2.47 (tt, *J* = 7.6, 2.4 Hz, 2H), 2.43 – 2.33 (m, 2H), 2.29 (ddd, *J* = 13.9, 4.9, 1.2 Hz, 1H), 2.25 – 2.18 (m, 4H), 2.16 (s, 1H), 1.79 (s, 3H); ^13^C NMR (150 MHz, chloroform-*d*): δ 157.7, 142.4, 140.1, 126.0, 124.5, 121.4, 113.3, 108.3, 82.3, 76.8, 67.8, 55.4, 44.7, 32.9, 27.2, 22.5, 19.8, 11.2; IR (thin film): 3441, 2933, 2835, 1585, 1463, 1439, 1258, 1101 cm;^−1^ HRMS (ESI–TOF) calculated for C_18_H_25_O_2_ [M+H]^+^ 273.1855, found 273.1855;$[\alpha ]_{\mathrm{D}}^{22} = - 1.1$ (0.05, CHCl_3_).

### Synthesis of hydrindane 23

To a stirring solution of 1-(trimethylsilyl)propyne (**3**) (4.5 g, 40 mmol, 3.1 equiv) and Ti(O*i*-Pr)_4_ (11 g, 38 mmol, 3.0 equiv) in 200 mL of anhydrous toluene at −78 °C under N_2_ atmosphere was added *n*-BuLi (2.3 M in hexanes, 33 mL, 76 mmol, 5.9 equiv) dropwise. After the addition, the cooling bath was removed, and the resulting dark brown mixture was warmed to rt, and then further warmed to 50 °C. The reaction mixture was stirred for 50 min at the same temperature without a reflux condenser, and then cooled to rt. A separate round bottom flask charged with a solution of **22** (3.5 g, 13 mmol, 1.0 equiv) in 50 mL of anhydrous toluene at −78 °C under N_2_ atmosphere was added *n*-BuLi (2.3 M in hexanes, 5.5 mL, 13 mmol, 1.0 equiv) dropwise. The resulting solution was warmed to rt, cannulated into the above dark brown mixture, and then stirred overnight at rt under N_2_ atmosphere (approx. 12 h). After this period, 100 mL of saturated solution of NH_4_Cl was added, and the mixture was further diluted with 100 mL of ethyl acetate. The organic layer was separated, and the aqueous layer was extracted with 250 mL × 2 ethyl acetate. The combined organic layers were dried with Na_2_SO_4_, filtered, and the filtrate was concentrated in vacuo. SiO_2_ flash column chromatography afforded the title compound **23** as a yellow oil (2.6 g, 53% isolated yield); Spectral Data for **23**: ^1^H NMR (500 MHz, chloroform-*d*): δ 7.07 (t, *J* = 7.9 Hz, 1H), 6.71 (d, *J* = 7.9 Hz, 2H), 4.41 – 4.31 (m, 1H), 3.81 (s, 3H), 2.73 – 2.62 (m, 2H), 2.57 (dt, *J* = 13.5, 8.0 Hz, 1H), 2.44 – 2.37 (m, 1H), 2.35 – 2.27 (m, 1H), 2.20 (s, 3H), 2.16 (d, *J* = 15.5 Hz, 1H), 2.05 – 1.97 (m, 3H), 1.95 (app d, *J* = 2.7 Hz, 3H), 1.40 (dd, *J* = 12.4, 7.5 Hz, 1H), 1.20 (s, 1H), 0.79 (s, 3H), 0.16 (s, 9H); ^13^C NMR (150 MHz, Chloroform-*d*): δ 157.8, 144.4, 141.8, 141.3, 129.5, 128.5, 126.0, 124.8, 122.3, 108.0, 72.1, 55.7, 51.3, 41.6, 39.4, 38.5, 33.2, 30.5, 21.4, 19.2, 11.4, 0.2; IR (thin film): 3349, 2950, 1585, 1465, 14371102, 1063, 1063, 835 cm;^−1^ HRMS (ESI–TOF): calculated for C_24_H_37_O_2_Si [M+H]^+^ 385.2563, found 385.2563; $[\alpha ]_{\mathrm{D}}^{22} = - 39.3$ (*c* 0.014, CHCl_3_).

### Synthesis of tetracycle 24

To a stirring suspension of (*S*)-BINOL (6.1 g, 21 mmol, 5.3 equiv) in 200 mL of dichloromethane at −78 °C under N_2_ atmosphere was added a solution of SnCl_4_ (1.0 M in dichloromethane, 21 mL, 21 mmol, 5.3 equiv) dropwise using syringe. The resulting mixture was stirred for 29 min at −78 °C, and then a solution of **23** (1.5 g, 4.0 mmol, 1.0 equiv) in 80 mL of dichloromethane was added dropwise via cannula transfer. The resulting mixture was stirred for 1.3 hr at −78 °C, and then warmed to rt over 30 min. The reaction was judged to be complete by TLC-analysis, and 100 mL of saturated solution of NH_4_Cl was added. The organic layer was separated, washed with 200 mL 5% solution of NaOH. The aqueous layer was further extracted with 200 mL of dichloromethane. The combined organic layers were dried over Na_2_SO_4_, filtered, and the filtrate was concentrated in vacuo. SiO_2_ flash column chromatography afforded the title compound **24** as an amorphous white solid (0.96 g, 78%); Spectral Data for **24**: ^1^H NMR (600 MHz, chloroform-*d*) δ 7.14 (d, *J* = 8.7 Hz, 1H), 6.77 (d, *J* = 8.7 Hz, 1H), 4.63 – 4.57 (m, 1H), 3.80 (s, 3H), 2.92 (dd, *J* = 14.5, 5.7 Hz, 1H), 2.85 (dd, *J* = 16.7, 8.7 Hz, 1H), 2.54 – 2.46 (m, 2H), 2.38 – 2.27 (m, 2H), 2.16 (dd, *J* = 11.9, 6.7 Hz, 1H), 2.13 – 2.05 (m, 4H), 1.85 – 1.78 (m, 1H), 1.71 (dt, *J* = 9.3, 3.1 Hz, 2H), 1.57 (s, 1H), 1.38 – 1.32 (m, 4H), 0.89 (s, 3H); ^13^C NMR (150 MHz, chloroform-*d*) δ 155.0, 140.4, 135.9, 131.8, 124.1, 123.7, 108.7, 71.7, 55.7, 52.1, 41.5, 38.3, 37.7, 35.0, 33.6, 31.6, 29.7, 25.9, 24.8, 11.5; IR (thin film): 3344, 2934, 2856, 2833, 1595, 1482, 1263, 1101, 755; HRMS (ESI–TOF): calculated for C_21_H_29_O_2_ [M+H]^+^ 313.2168, found 313.2162; $[\alpha ]_{\mathrm{D}}^{22} = - 169.71$ (*c* 0.0046, CHCl_3_).

### Synthesis of enone 25

To a stirring solution of **24** (1.5 g, 4.7 mmol, 1.0 equiv) in 50 mL of anhydrous toluene at rt under N_2_ atmosphere was added DIBAL-H (1.0 M in hexanes, 47 mL, 47 mmol, 10 equiv). The resulting mixture was warmed to 100 °C, refluxed overnight (approx. 16 h), and then cooled to rt. Small chunks of ice was slowly added, and the resulting mixture was diluted with 200 mL water, 10 mL 1 M solution of HCl, and 250 mL of ethyl acetate. The separated organic layer was dried with Na_2_SO_4_, filtered, and concentrated in vacuo to afford 1.41 g of the crude product as an amorphous yellow solid. The crude product (1.4 g) at 0 °C under N_2_ atmosphere was added with 50 mL of HFIP followed by PIDA (1.4 g, 4.3 mmol). The resulting mixture was stirred for 1 min at 0 °C (PIDA was fully dissolved at this point), and then 30 mL of saturated solution of NaHCO_3_ was added. HFIP was removed in vacuo, and the remaining aqueous mixture was extracted with 100 mL × 3 ethyl acetate. The combined organic layers were dried over Na_2_SO_4_, filtered, and the filtrate was concentrated in vacuo. The crude product was purified with SiO_2_ flash column chromatography to afford the title compound **25** as an amorphous white solid (0.76 g, 54% over 2 steps). Spectral data for **25**: ^1^H NMR (500 MHz, chloroform-*d*) δ 7.14 (d, *J* = 10.1 Hz, 1H), 6.28 (d, *J* = 10.1 Hz, 1H), 5.50 (s, 1H), 5.11 – 5.02 (m, 1H), 2.97 (ddd, *J* = 13.0, 6.0, 1.7 Hz, 1H), 2.71 (ddt, *J* = 16.5, 6.3, 2.0 Hz, 1H), 2.53 – 2.39 (m, 2H), 2.34 (app dd, *J* = 12.1, 6.5 Hz, 2H), 2.18 (ddtd, *J* = 18.6, 10.3, 4.1, 2.0 Hz, 1H), 1.95 (s, 3H), 1.82 (ddd, *J* = 12.6, 5.0, 1.4 Hz, 1H), 1.64 (s, 1H), 1.57 (td, *J* = 12.4, 5.6 Hz, 2H), 1.45 (s, 3H), 1.40 (dd, *J* = 12.1, 7.6 Hz, 1H), 0.88 (s, 3H); ^13^C NMR (150 MHz, chloroform-*d*) δ 185.4, 159.4, 151.9, 149.9, 137.9, 128.3, 127.6, 125.4, 124.3, 76.6, 51.7, 44.8, 43.3, 36.2, 28.8, 28.4, 25.4, 24.7, 23.6, 10.5; IR (thin film): 3404, 2923, 2851, 1659, 1625,1607, 1449, 1051, 833, 753 cm;^−1^ HRMS (ESI–TOF): calculated for C_20_H_25_O_2_[M+H]^+^ 297.1855, found 297.1856; $[\alpha ]_{\mathrm{D}}^{22} = + 156.27$ (*c* 0.0032, CHCl_3_).

### Synthesis of tetracycle 26

To a stirring solution of **S7** (2.9 g, 9.3 mmol, 1.0 equiv) and water (3.6 g, 200 mmol, 22 equiv) in 190 mL dimethoxyethane was added Co_2_(CO)_8_ (8.0 g, 23 mmol, 2.5 equiv). The resulting mixture was refluxed for 3 h at 90 °C, and for 1 h at 100 °C. The reaction mixture was cooled to rt, and another portion of Co_2_(CO)_8_ (3.2 g, 9.4 mmol, 1.0 equiv) was added. The reaction mixture was warmed to 90 °C again, continued to reflux for 1.7 h, and then cooled to rt. The resulting mixture was passed through a pad of silica gel using 850 mL of ethyl acetate, 100 mL 5% methanol/95% ethyl acetate, and 10% methanol/90% ethyl aceate as the eluent, and then the filtrate was concentrated in vacuo. The crude product was purified by dry column vacuum chromatography^1^ using 200 mL of dichloromethane and then a gradient elution (20% ethyl acetate/80% hexanes to 50% ethyl aceate/50% hexanes, 5% increase in ethyl acetate/ fraction, 500 mL/fraction) to afford 2.02 g of the title compound **26** as an amourphous white solid (69% yield). Spectral data for **26**: ^1^H NMR (600 MHz, chloroform-*d*) δ 4.63 (td, *J* = 7.7, 4.0 Hz, 1H), 2.72 (ddd, *J* = 12.9, 4.9, 2.3 Hz, 1H), 2.51 (ddd, *J* = 17.3, 14.7, 5.1 Hz, 1H), 2.42 (ddd, *J* = 17.3, 4.8, 2.7 Hz, 1H), 2.24 – 2.07 (m, 4H), 1.94 (dt, *J* = 7.4, 3.5 Hz, 1H), 1.86 – 1.66 (m, 7H), 1.65 – 1.58 (m, 3H), 1.30 (s, 3H), 0.96 (dd, *J* = 12.5, 8.7 Hz, 1H), 0.88 (s, 3H), 0.82 (dd, *J* = 5.1, 3.1 Hz, 1H), 0.38 (dd, *J* = 7.5, 5.2 Hz, 1H); ^13^C NMR (150 MHz, chloroform-*d*) δ 198.7, 163.5, 133.3, 129.5, 127.4, 73.7, 41.4, 40.0, 38.9, 37.7, 34.0, 33.4, 31.6, 26.33, 26.26, 26.1, 23.4, 23.0, 21.7, 10.8, 10.1; IR (thin film): 3399, 2922, 1660, 1613, 1450, 1373, 1357, 1332, 1074, 1054, 1025 cm;^−1^ HRMS: calculated for C_21_H_29_O_2_ [M+H]^+^ 313.2168, found 313.2164; $[\alpha ]_{\mathrm{D}}^{22} = + 252.2$ (*c* 0.021, CHCl_3_).

### Synthesis of tetracycle 27

To a stirring blue solution of Li (20 mg, 2.9 mmol, 10 equiv) in 10 mL NH_3_ (l) at −78 °C under N_2_ atmosphere was added a solution of **26** (89 mg, 0.28 mmol, 1.0 equiv) in 2 mL THF dropwise. The resulting mixture was stirred for 15 min at −78 °C, warmed to −35 °C, and then stirred for 50 min while maintaining the cold bath temperature between −35 °C and −25 °C. After this period, the reaction mixture was cooled to −78 °C, and then MeI (0.81 g, 5.7 mmol, 20 equiv) was added dropwise. The cold bath was removed, and NH_3_ (l) was evaporated under a stream of N_2_ over 30 min. In total, 5 -mL saturated solution of NH_4_Cl was added, and the resulting mixture was further diluted with 10 mL of ethyl acetate and 5 mL of water. The organic layer was separated, and the aqueous layer was extracted with 25 mL × 2 ethyl acetate. The combined organic layers were dried with Na_2_SO_4_, filtered, and the filtrate was concentrated in vacuo. SiO_2_ flash column chromatography afforded the title compound **27** as a pale yellow film (48 mg, 52%); Spectral data for **27**: ^1^H NMR (500 MHz, chloroform-*d*) δ 4.69 – 4.59 (m, 1H), 2.57 (ddd, *J* = 15.9, 10.4, 7.5 Hz, 1H), 2.46 (ddd, *J* = 15.9, 7.5, 3.8 Hz, 1H), 2.20 – 2.07 (m, 2H), 2.07 – 2.00 (m, 1H), 1.94 (dt, *J* = 7.4, 3.5 Hz, 1H), 1.81 – 1.71 (m, 1H), 1.71 – 1.56 (m, 7H), 1.45 (dq, *J* = 18.4, 6.2 Hz, 1H), 1.36 (br s, 1H), 1.09 (s, 3H), 1.08 (s, 3H), 1.05 (s, 3H), 0.97 – 0.90 (m, 4H), 0.75 (dd, *J* = 5.0, 3.1 Hz, 1H), 0.32 (dd, *J* = 7.5, 5.0 Hz, 1H); ^13^C NMR (150 MHz, chloroform-*d*) δ 217.9, 134.8, 128.0, 73.9, 51.7, 47.4, 41.7, 39.1, 38.1, 37.5, 35.5, 34.6, 31.9, 26.79, 26.76, 26.3, 22.9, 21.9, 21.3, 19.9, 19.7, 10.2; IR (thin film): 3392, 2950, 2924, 1704, 1473, 1457, 1383, 1058, 1020, 754 cm;^−1^ HRMS: calculated for C22H31O2 [M–H]^+^ 327.2324, found 327.2325; $[\alpha ]_{\mathrm{D}}^{22} = + 82.0$ (*c* 0.0065, CHCl_3_).

### Cell lines and reagents

Human GBM cells U87, U251 were obtained from American Type Culture Collection (ATCC, Manassas, VA) and were cultured as per ATCC guidelines. Adult human neural progenitor cells were a generous gift from Drs. Steindler & Tong, Tufts University. Human astrocytes were obtained from ThermoFisher (Catalog number: N7805100). Following standard laboratory protocols, all study model cells utilized were determined to be free of mycoplasma contamination, and were confirmed by using MycoSEQ™ Mycoplasma Detection Kit, with Discriminatory Positive Control (ThermoFisher; Catalog number: 4460623).

### Cell survival/proliferation and IC_50_ assays

To determine growth inhibition, U87 and U251 cells plated in 96-well plates for 24 h were treated with varying doses of **28**, ***ent*-28**, TMZ or vehicle control DMSO for 96 h. Cells were counted every 24 h after initiating treatment. This was done after the wells were first washed with PBS and the remaining adherent, viable cells in each well were trypsinized and counted using a hemocytometer following trypan blue exclusion. The data were normalized to untreated cell counts obtained from cells prior to initiating treatment at 0 h. The data were analyzed from four replicates and each experiment was repeated at least three times.

### Flow-cytometric analysis of apoptotic cell death

U87 and U251 cells were seeded in six-well plates (300,000 cells/well) 24 h prior to treatment. Each well was treated with various concentrations of **28**, ***ent*-28**, TMZ, or vehicle control DMSO for 24 h. DMSO was used as a negative control. The cells were then washed and stained with Annexin V-FITC and 7-AAD (BioLegend), according to the manufacturer’s instructions, and were analyzed on a MACSQuant^®^ Analyzer 10 (Miltenyi Biotec Inc.). Staurosporine (STS) treatment at 1 μM served as a positive control for Annexin V staining and DMSO was used as a negative control. The files were converted to FCS compatible format and analyzed using FlowJo analysis software (Treestar). The quantitative analysis of the percentage of apoptotic cells was reported from three independent experiments.

### Cell morphology studies of 28 and *ent*-28 versus TMZ

U87 and U251 cell lines, adult human neural progenitor cells (a generous gift from Drs. Steindler & Tong, Tufts University), and human astrocytes (ThermoFisher) were seeded in a 96-well plate format with a density of 1000 cells per well and incubated for 24 h before treatment. Each well was then supplemented with fresh media containing various concentrations of **28**, ***ent*-28**, TMZ, or vehicle control DMSO. Phase-contrast images at ×10 magnification were acquired using the IncuCyte^®^ Live-Cell Imaging System (Essen Instruments). Each well was imaged four times every 24 h over 5 days and image-based analysis of cell morphology was carried out using the Incucyte Software. The results are representative of four independent experiments.

### Cell-viability assays

Cell viability after treatment in quadruplicate in 96-well plates was detected by the MTT assay. U87 and U251 cells plated as described above were treated with **28**, ***ent-*28**, and TMZ with a DMSO control over 96 h. Every 24 h, the medium was removed and 20 μL of MTT (5 mg/mL) was added for 4 h. After removing supernatant, 100 μL of DMSO was added to resolve formazan crystals, and the plates were measured at 595 nm with a SpectraMax^®^ i3x microplate reader (Molecular Devices, Sunnyvale, CA, USA). The data were collected using Softmax Pro 6.5.1 software (Molecular Devices). The results are representative of at least three independent experiments.

### Evaluation of 28 and *ent*-28 in DU-145 prostate cancer cells

Human DU-145 prostate cancer cells were plated at a density of 10,000 cells per well (96-well plates) in RPMI + 10% fetal bovine serum (FBS) without phenol red. Two days after plating, cells were treated with compounds (5 µM) for 24 h in RPMI + 10% charcoal-stripped FBS without phenol red. ALP activity was determined using the ThermoFisher Scinetific 1-step PNPP assay according to the manufacturer’s protocol.

### Reporting summary

Further information on research design is available in the Nature Research Reporting Summary linked to this article.

## Supplementary information

Supplementary Information

Reporting Summary

Source Data

## Data Availability

All relevant data are available from the authors, and are included with the manuscript in the accompanying Supplementary Information. These include characterization for all new compositions of matter (organic molecules), as well as copies of relevant NMR spectra, experimental procedures, and supplemental figures associated with the biological evaluation of **28** and ***ent*****-28** (data associated with these evaluations are provided in the Manuscript and Supplementary Information; raw data is available upon request). X-ray crystallographic coordinates for compound **24** have been deposited at the Cambridge Crystallographic Data Centre (CCDC), under deposition number CCDC 1897943. These data can be obtained free of charge from The Cambridge Crystallographic Data Centre via www.cdc.cam.ac.uk/data_request/cif. The raw data underlying Figs. 4 and 5, as well as Supplementary Figs. 1, 2, 4, and 5 are provided in a Source Data File.

## References

[1] Bachmann WE, Cole W, Wilds AL (1939). The total synthesis of the sex hormone equilenin. J. Am. Chem. Soc..

[2] Lednicer D (2011). Steroid Chemistry at a GLance.

[3] Pines SH (2004). The Merck bile acid cortisone process: the next-to-last word. Org. Proc. Res. Dev..

[4] Marker RE, Tsukamoto T, Turner DL, Sterols. C (1940). Diosgenin. J. Am. Chem. Soc..

[5] Renneberg R (2008). Mexico, the father of the pill and the race for cortisone. Biotechnol. J..

[6] Hanson JR (2010). Steroids: Partial synthesis in medicinal chemistry. Nat. Prod. Rep..

[7] Zeelen FJ (1994). Steroid total synthesis. Nat. Prod. Rep..

[8] Chapelon A, Moraléda D, Rodriguez R, Ollivier C, Santelli M (2007). Enantioselective synthesis of steroids. Tetrahedron.

[9] Mackay EG, Sherburn MS (2015). The Diels–Alder reaction in steroid synthesis. Synthesis.

[10] Vollhardt KPC (1985). Cobalt-mediated steroid synthesis. Pure Appl. Chem..

[11] Kaplan W, Khatri HR, Nagorny P (2016). Concise enantioselective total synthesis of cardiotonic steroids 19-hydroxysarmentogenin and trewianin aglycone. J. Am. Chem. Soc..

[12] Nising CF, Bräse S (2008). Highlights in steroid chemistry: total synthesis versus semisynthesis. Angew. Chem. Int. Ed..

[13] Bartlett WR, Johnson WS, Plummer MS, Small VR (1990). Cationic cyclization of a substrate having an internal acetylenic bond. Synthesis of euphol and tirucallol. J. Org. Chem..

[14] Biellmann JF (2003). Enantiomeric steroids: synthesis, physical, and biological properties. Chem. Rev..

[15] Covey DF (2009). *ent*-Steroids: novel tools for studies of signaling pathways. Steroids.

[16] Green PS (2001). The nonfeminizing enantiomer of 17beta-estradiol exerts protective effects in neuronal cultures and a rat model of cerebral ischemia. Endocrinology.

[17] Petit GH (2011). Pregnenolone sulfate and its enantiomer: differential modulation of memory in a spatial discrimination task using forebrain NMDA receptor deficient mice. Eur. Neuropsychopharmacol..

[18] Kim WS, Du K, Eastman A, Hughes RP, Micalizio GC (2017). Synthetic *nat-* or *ent*- steroids in as few as five chemical steps from epichlorohydrin. Nat. Chem..

[19] Greszler SN, Reichard HA, Micalizio GC (2012). Asymmetric synthesis of dihydroindanes by convergent alkoxide-directed metallacycle-mediated bond formation. J. Am. Chem. Soc..

[20] Wengryniuk, S. E. & Canesi, S. Rearrangements and Fragmentations Mediated by Hypervalent Iodine Reagents*, in PATAI’s Chemistry of Functional Groups*. 10.1002/9780470682531.pat0939 (John Wiley & Sons, Ltd., Hoboken, 2018).

[21] Guérard KC (2012). Oxidative 1,2- and 1,3-alkyl shift processes: developments and applications in synthesis. J. Org. Chem..

[22] Guérard KC (2009). An unprecedented oxidative Wagner–Meerwein transposition. Org. Lett..

[23] Dai M, Krauss IJ, Danishefsky SJ (2008). Total synthesis of spirotenuipesines A and B. J. Org. Chem..

[24] Ishihara K, Nakamura S, Yamamoto H (1999). The first enantioselective biomimetic cyclization of polyprenoids. J. Am. Chem. Soc..

[25] Surendra K, Corey EJ (2012). Highly enantioselective proton-initiated polycyclization of polyenes. J. Am. Chem. Soc..

[26] Hilscher, J.-C. *Steroid Ether Splitting*. US 3,956,348, United States Patent and Trademark Office (1975).

[27] Charette AB, Beauchemin A (2001). Simmons-Smith cyclopropanation reaction. Org. React..

[28] Lee H-Y, An M (2003). Selective 1,4-reduction of unsaturated carbonyl compounds with Co_2_(CO)_8_–H_2_O. Tetrahedron Lett..

[29] Kuiper GGJM, Enmark E, Pelto-Huikko M, Nilsson S, Gustafsson J (1996). Cloning of a novel estrogen receptor expressed in rat prostate and ovary. Proc. Natl Acad. Sci. USA.

[30] Warner M, Huang B, Gustafsson J (2017). Estrogen receptor β as a pharmaceutical target. Trends Pharm. Sci..

[31] Thomas CG, Strom A, Lindberg K, Gustafsson J (2011). Estrogen receptor beta decreases survival of p53-defective cancer cells after DNA damage by impairing G_2_/M checkpoint signaling. Breast Cancer Res. Treat..

[32] Bossard C (2012). Potential role of estrogen receptor beta as a tumor suppressor of epithelial ovarian cancer. PLOS-one.

[33] Sareddy GR (2012). Therapeutic significance of estrogen recepter β agonists in gliomas. Mol. Cancer Ther..

[34] Hinsche O, Girgert R, Emons G, Gründker C (2015). Estrogen receptor β selective agonists reduce invasiveness of triple-negative breast cancer cells. Int. J. Oncol..

[35] Rota S (2017). Estrogen receptor β is a novel target in acute myeloid leukemia. Mol. Cancer Ther..

[36] Schüler-Toprak S, Moehle C, Skrzypczak M, Ortmann O, Treeck O (2017). Effect of estrogen receptor β agonists on proliferation and gene expression of ovarian cancer cells. BMC Cancer.

[37] McFarland K (2013). AC-186, a selective nonsteroidal estrogen receptor β agonist, shows gender specific neuroprotection in a Parkinson’s disease rat model. ACS Chem. Neurosci..

[38] Ponnusamy S (2017). Pharmacologic activation of estrogen receptor β increases mitochondrial function, energy expenditure, and brown adipose tissue. FASEB.

[39] Leventhal L (2006). An estrogen receptor-β agonist is active in models of inflammatory and chemical-induced pain. Eur. J. Pharm..

[40] Cvoro A (2008). Selective estrogen recptor-β agonists repress transcription of proinflammatory genes. J. Immunol..

[41] Gardell LR (2008). Differential modulation of inflammatory pain by a selective estrogen receptor beta agonist. Eur. J. Pharm..

[42] Ma J-N, McFarland K, Olsson R, Burstein ES (2016). Estrogen receptor beta selective agonists as agents to treat chemotherapeutic-induced neuropathic pain. ACS Chem. Neurosci..

[43] Kosemund, D., Mueller, G., Hillisch, A., Fritzemeier, K.-H. & Muhn, P. *9-α-Substituted Estratrienes as Selectively Active Estrogens*. US 7,414,043 B2, United States Patent and Trademark Office, (2008).

[44] Manas ES (2004). Structure-based design of estrogen receptor-β selective ligands. J. Am. Chem. Soc..

[45] Helguero LA, Faulds MH, Gustafsson J-Å, Haldosén LA (2005). Estrogen receptors alfa (ERα) and beta (ERβ) differentially regulate proliferation and apoptosis of the normal murine mammary epithelial cell line HC11. Oncogene.

[46] Sauvée C (2013). The A-CD analogue of 16β,17α-estriol is a potent and hihgly selective estrogen receptor β agonist. Med. Chem. Comm..

[47] Chen L (2014). Selective ligands of estrogen receptor β discovered using pharmacophore mapping and structure-based virtual screening. Acta Pharm. Sin..

[48] Weiser MJ, Wu TJ, Handa RJ (2009). Estrogen receptor-β agonist diarylpropionitrile: Biological activities of *R*- and *S*-enantiomers on behavior and hormonal response to stress. Neuroendocrin.

[49] Blizzard TA (2006). Androstenediol analogs as ER-β-selective SERMs. Bioorg. Med. Chem. Lett..

[50] Weihua Z (2001). A role for estrogen receptor beta in the regulation of growth of the ventral prostate. Proc. Natl Acad. Sci. USA.

[51] Cheung CP (2005). Expression and functional study of estrogen receptor-related receptors in human prostatic cells and tissues. J. Clin. Endocrinol. Metab..

[52] Holinka CF, Hata H, Kuramoto H, Gurpide E (1986). Effects of steroid hormones and antisteroids on alkaloine phosphatase activity in human endometrial cancer cells (Ishikawa line). Cancer Res..

[53] Sareddy GR (2016). Selective estrogen receptor β agonist LY500307 as a novel therapeutic agent for glioblastoma. Sci. Rep..

[54] Batistatou A (2004). Estrogen receptor beta (ERbeta) is expressed in brain astrocytic tumors and declines with dedifferentiation of the neoplasm. J. Cancer Res. Clin. Oncol..

[55] Kefalopoulou Z (2012). Prognostic value of novel biomarkers in astrocytic brain tumors: nuclear receptor co-regulators AIB1, TIF2, and PELP1 are associated with high tumor grade and worse patient prognosis. J. Neurooncol..

[56] Ellem SJ, Risbridger GP (2007). Treating prostate cancer: a rationale for targeting local oestrogens. Nat. Rev. Cancer.

[57] Beral V, Bull D, Green J, Reeves G (2007). Ovarian cancer and hormone replacement therapy in the Million Women Study. Lancet.

[58] Schwarz, S. et al. *Ent-steroids as selectively active estrogens.* PCT/EPOO/03470, WO 00/63228 (2000).

