# Supplementary material for: A synthesis strategy for tetracyclic terpenoids leads to agonists of ERβ
Source: Nat Commun. 2019 Jun 4;10:2448. doi: 10.1038/s41467-019-10415-6 (PMC6547701; doi:10.1038/s41467-019-10415-6)
Supplement: Supplementary file 1 — Supplementary Information [file 41467_2019_10415_MOESM1_ESM.pdf]

Supplementary Information

A Synthesis Strategy for Tetracyclic Terpenoids Leads to Agonists of ERβ

Table of contents

1. **Supplementary Figures** **2**

2. **Supplementary Methods** **38**

3. **Supplementary Notes** **62**

4. **Supplementary References** **63**

Supplementary Figures

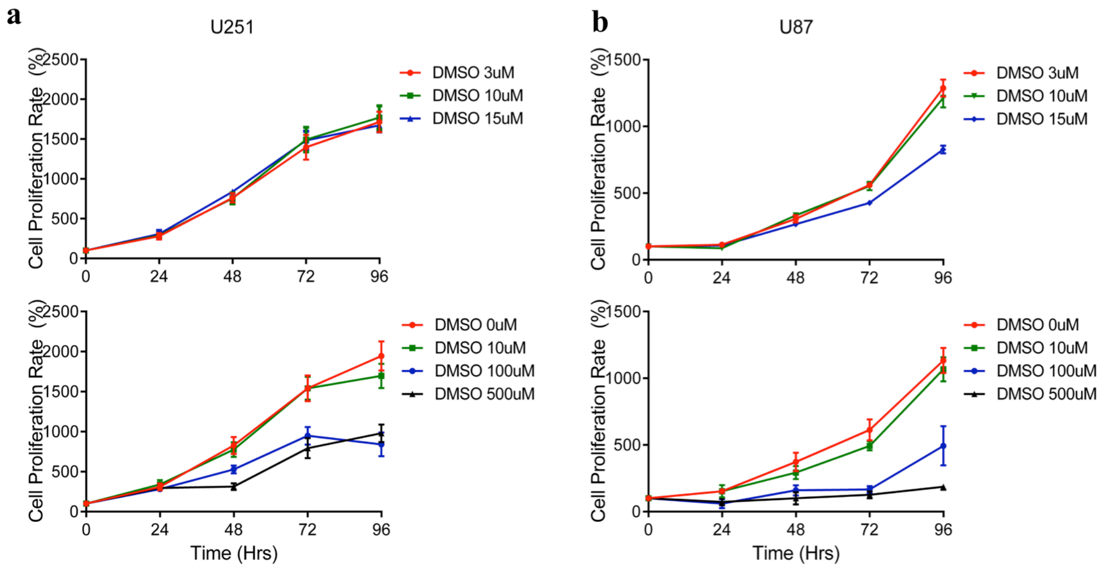

Supplementary Figure 1: **Cell viability and proliferation in glioblastoma cell lines treated with DMSO.** Viable cell counts of U251 and U87 were determined every 24 hours for 96 hours following dose dependent DMSO treatment and trypan blue exclusion (n=4). Data are representative of three independent experiments. Mean ± s.d. of quadruplicates from one experiment are presented on the line plots. Source data are provided as a Source Data File.

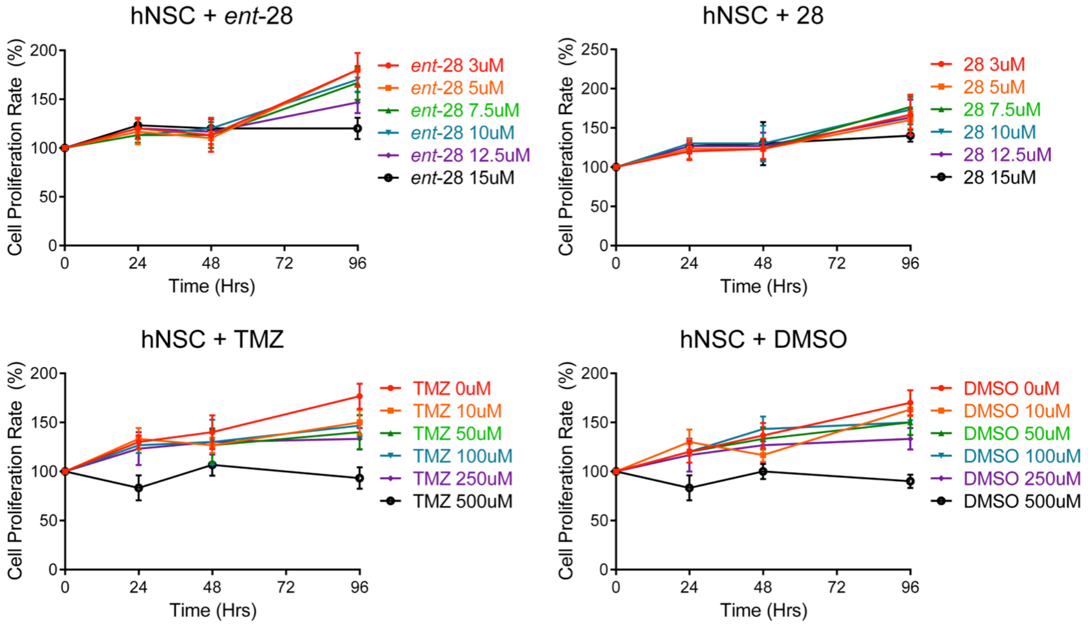

Supplementary Figure 2: **Cell viability and proliferation of human neural stem cells (hNSC) treated with *ent*-28 or 28.** Viable cell counts of hNSCs following dose dependent treatment with ***ent*-28, 28**, TMZ, and DMSO. Cell counts were determined after trypan blue exclusion every 24 hours for 96 hours (n=4). Data are representative of three independent experiments. Mean ± s.d. of quadruplicates from one experiment are presented on the line plots. Source data are provided as a Source Data File.

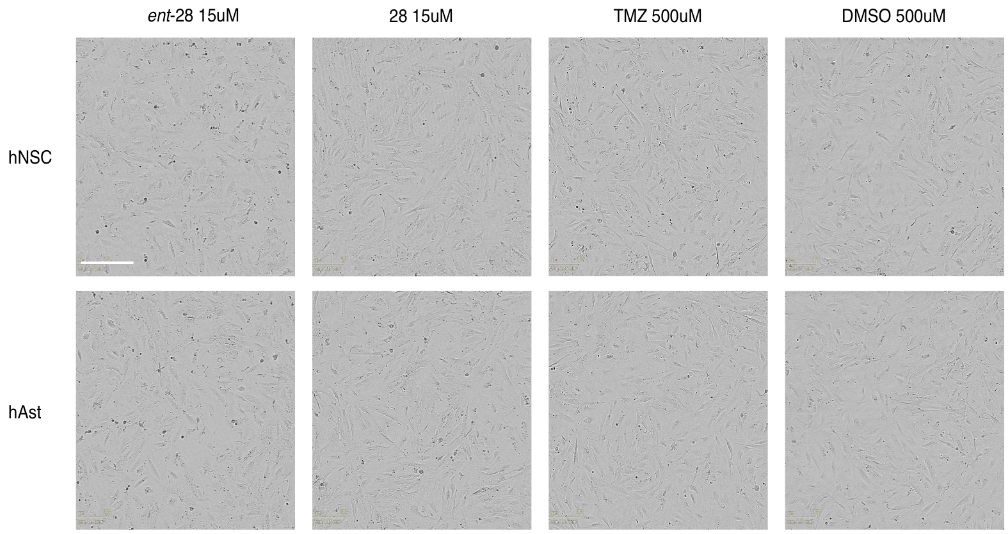

Supplementary Figure 3: **Morphological analyses of human neural stem cells (hNSC) and human astrocytes (hAst) treated with *ent*-28 or 28.** Phase-contrast images at 24 hours were taken using Incucyte for live cell tracking of hNSCs and human astrocytes in response to treatment with **28, *ent*-28**, TMZ and vehicle control DMSO. Live cells were tracked and imaged for 96 hours (data not shown). Scale bar = 300 µm. Data are representative of three independent experiments and four replicates of each treatment condition were analyzed per experiment.

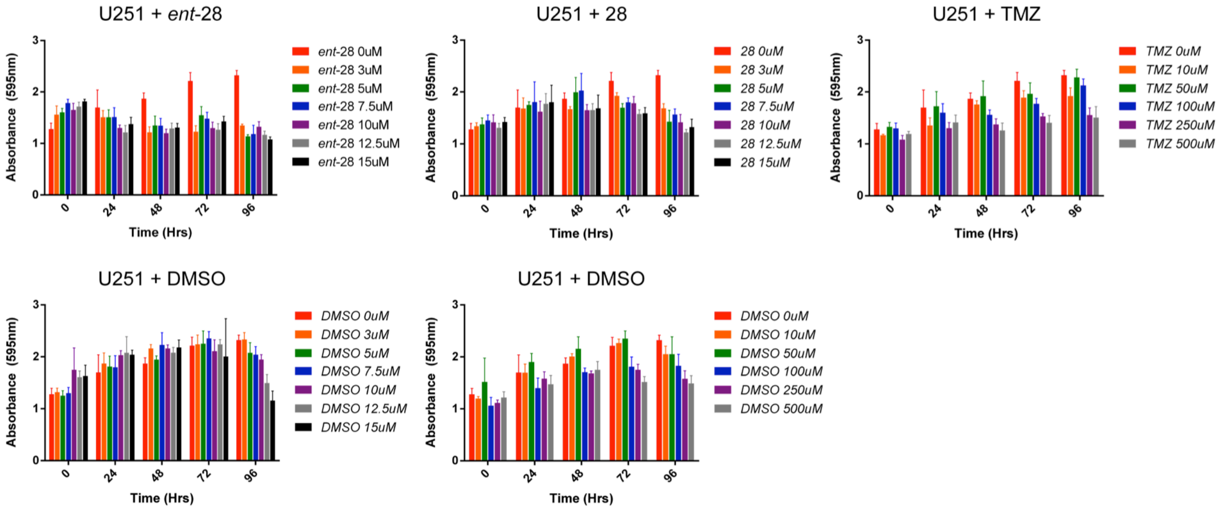

***Supplementary Figure 4: ent*-28 and 28 inhibit proliferation in U251 glioblastoma cell line.** Cell proliferation for U251 determined by MTT assay every 24 hours for 96 hours after incubation with varying concentrations of ***ent*-28, 28,** TMZ, and DMSO. Decreased absorbance (595nm) correlates to decreased proliferation. Data are representative of three independent experiments. Mean ± s.d. of quadruplicates from one experiment are presented on the bar graphs. Source data are provided as a Source Data File.

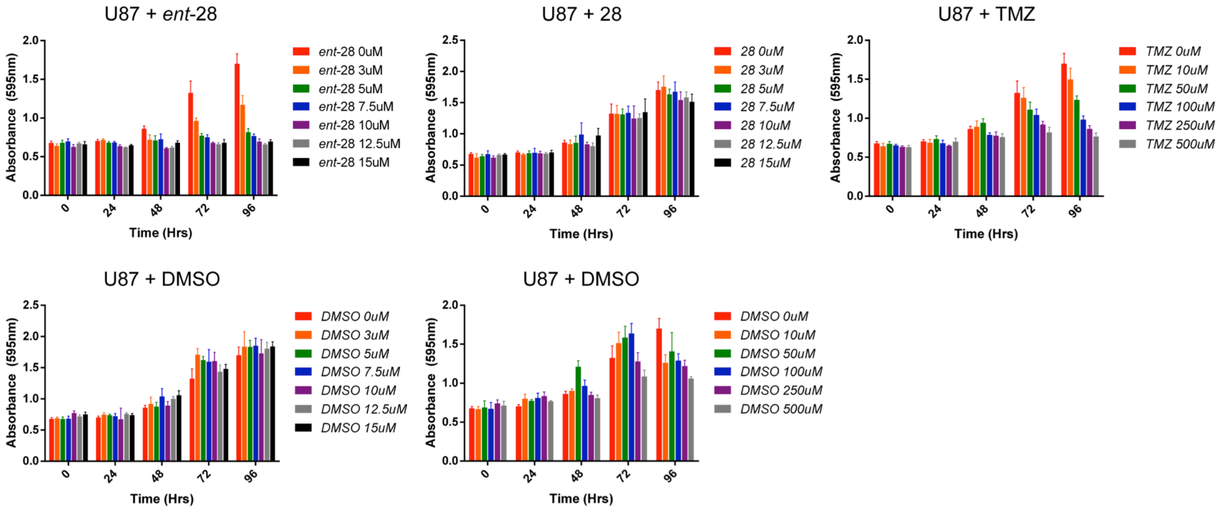

***Supplementary 5: ent*-28 and 28 inhibit proliferation in U87 glioblastoma cell line.** Cell proliferation for U87 determined by MTT assay every 24 hours for 96 hours after incubation with varying concentrations of ***ent*-28, 28,** TMZ, and DMSO. Decreased absorbance (595nm) correlates to decreased proliferation. Data are representative of three independent experiments. Mean ± s.d. of quadruplicates from one experiment are presented on the bar graphs. Source data are provided as a Source Data File.

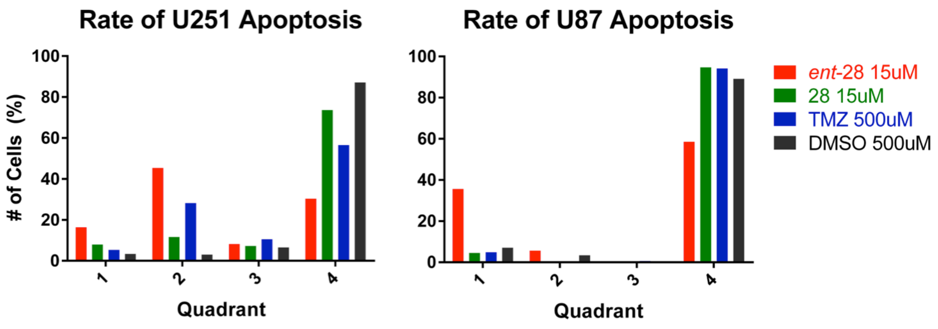

Quadrant 1: Increased Annexin V-FITC staining indicates increased apoptosis
Quadrant 2: Increased Annexin V-FITC and 7-AAD staining indicates both apoptosis and necrosis
Quadrant 3: Increased 7-AAD staining indicates increased necrosis
Quadrant 4: Low levels of Annexin V-FITC and 7-AAD staining indicates live cells

***Supplementary Figure 6: ent*-28 and 28 induce apoptosis in glioblastoma cell lines.** Histogram representing the percentage of necrotic, early and late apoptotic U251 and U87 cells following treatment with ***ent*-28, 28**, TMZ, and DMSO after 24 hours. Quadrant 1 represents annexin positive cells (early apoptotic) and quadrant 2 shows annexin and 7-AAD positive cells (late apoptosis and necrosis). Quadrant 3 shows 7-AAD positive cells (necrotic) and Quadrant 4 cells are negative for annexin and 7-AAD. Data are representative of three independent experiments.

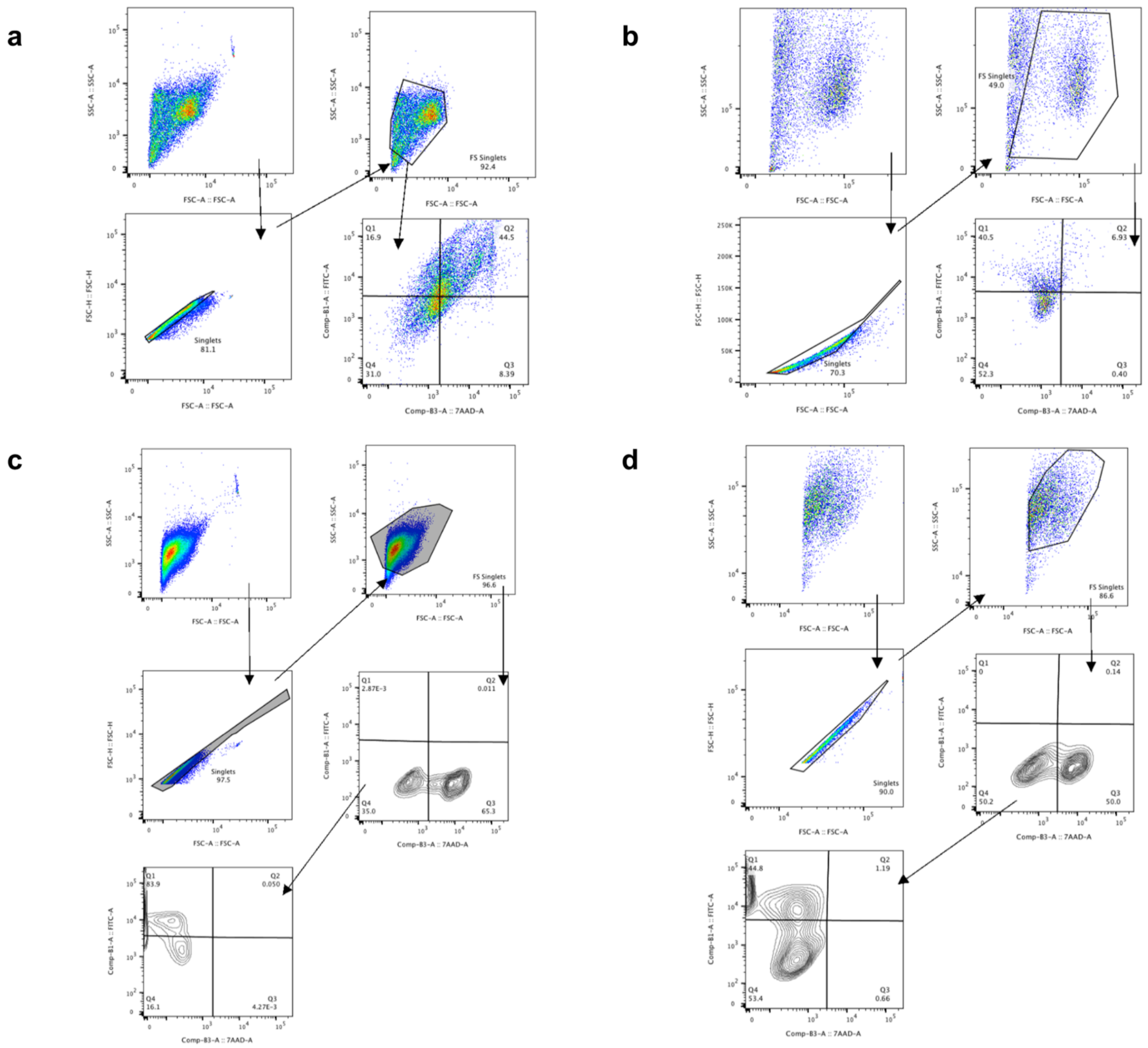

Supplementary Figure 7: **Gating strategy for U251 and U87 FSC/SSC and Annexin V-FITC/7-AAD. a, b,** Representative FSC and SSC profiles of U251 cells treated with *ent-28* (**a**) and U87 cells treated with *ent-28* (**b**). Doublets were excluded and single cells were gated from FSC-A/FSC-H plots and additionally in the FSC-A/SSC-A plots. **c, d,** FSC and SSC profiles of U251 cells treated with staurosporine (positive apoptosis inducing agent) and stained for Annexin V-FITC or 7-AAD (**c**) and U87 cells treated with staurosporine and stained for Annexin V-FITC or 7-AAD (**d**). Gating for FITC and 7-AAD were represented using contour plots.

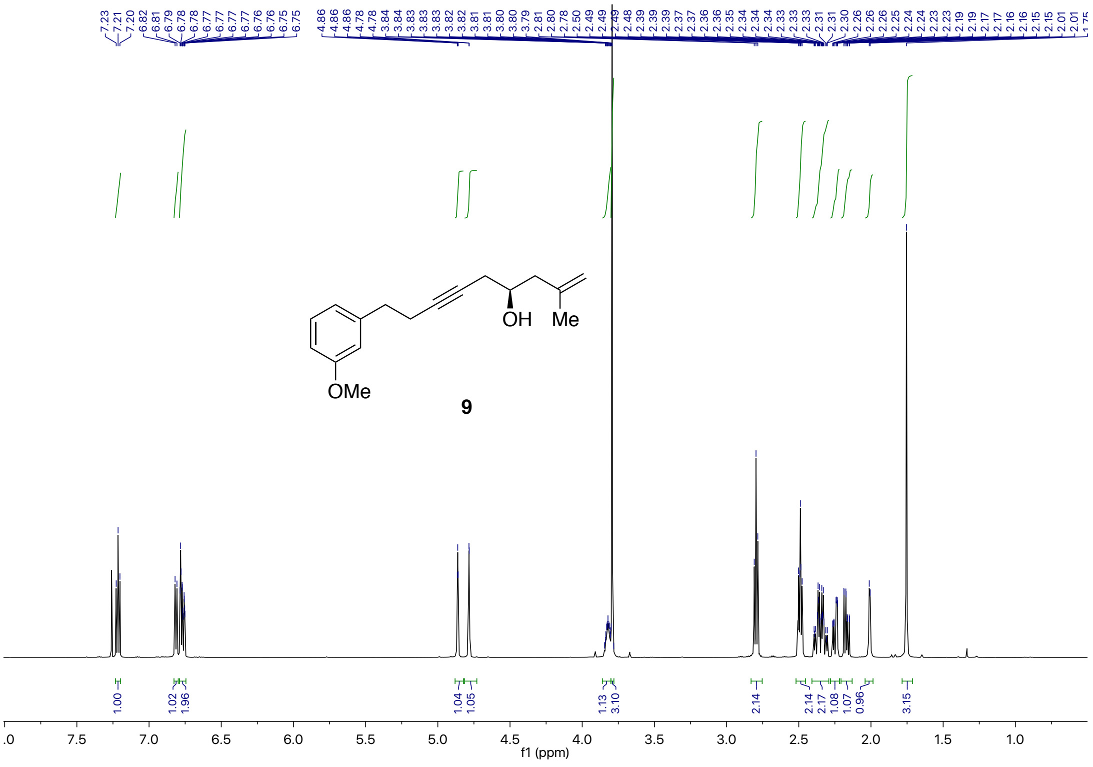

Supplementary Figure 8. ^{1}HNMR of **9**

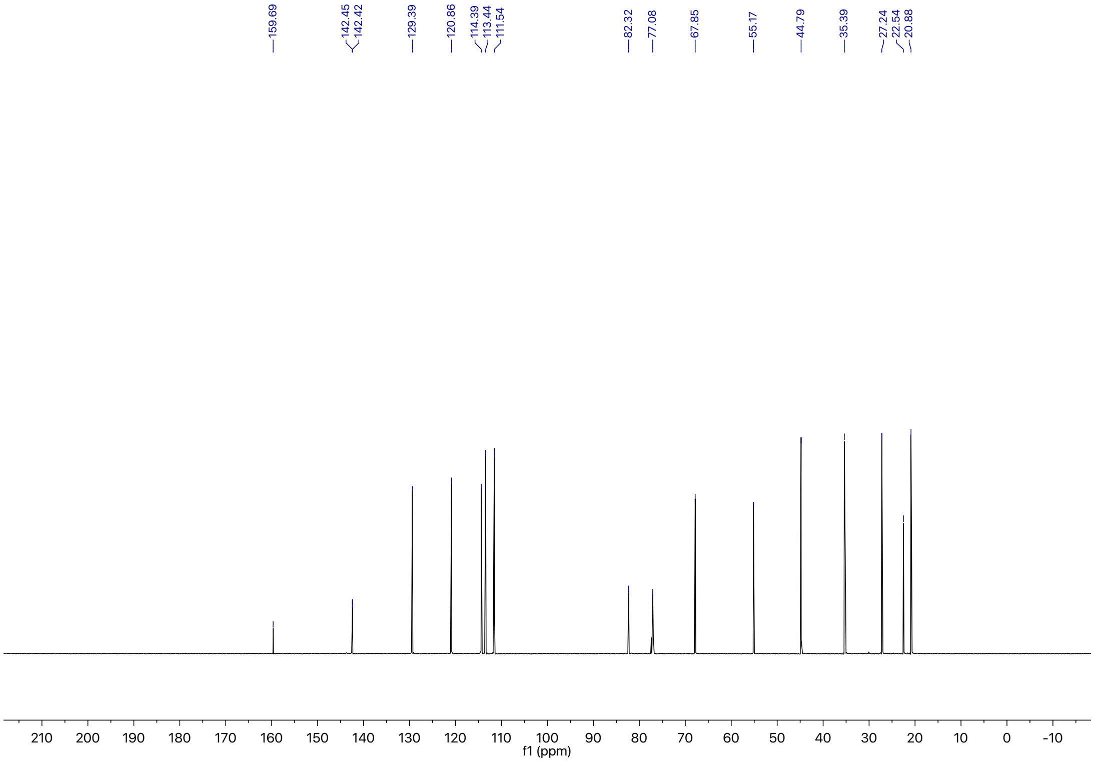

Supplementary Figure 9. ^{13}CNMR of **9**

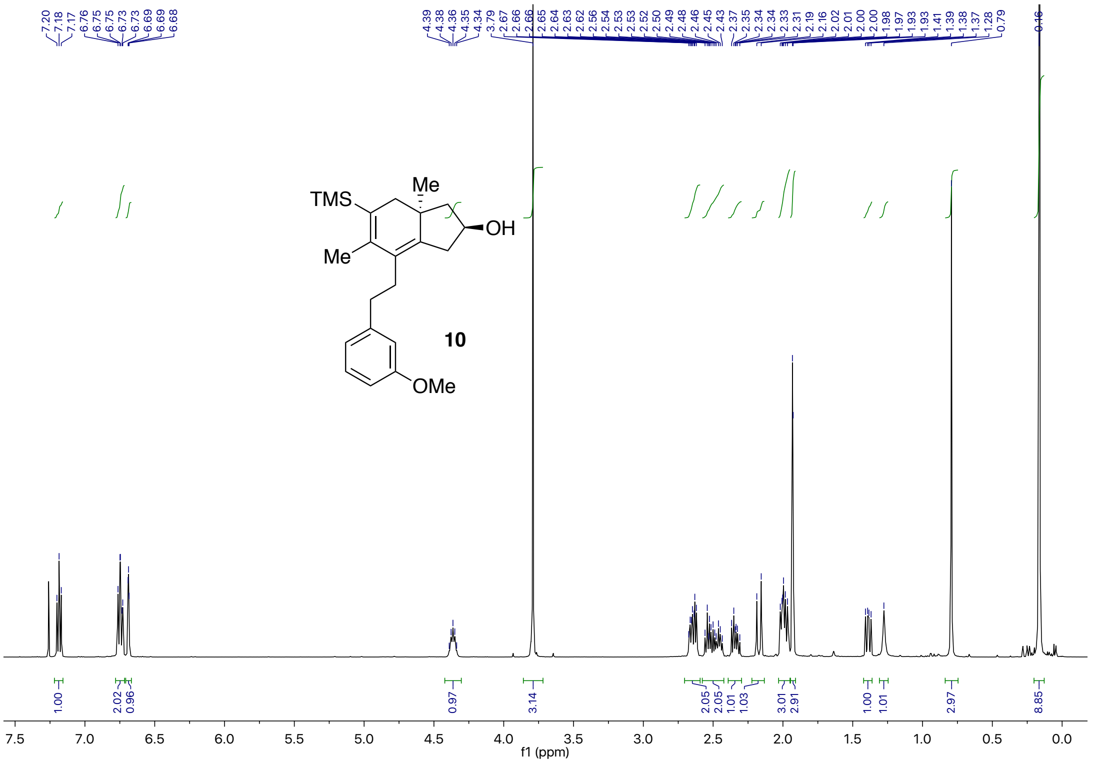

Supplementary Figure 10. ^{1}H NMR of **10**

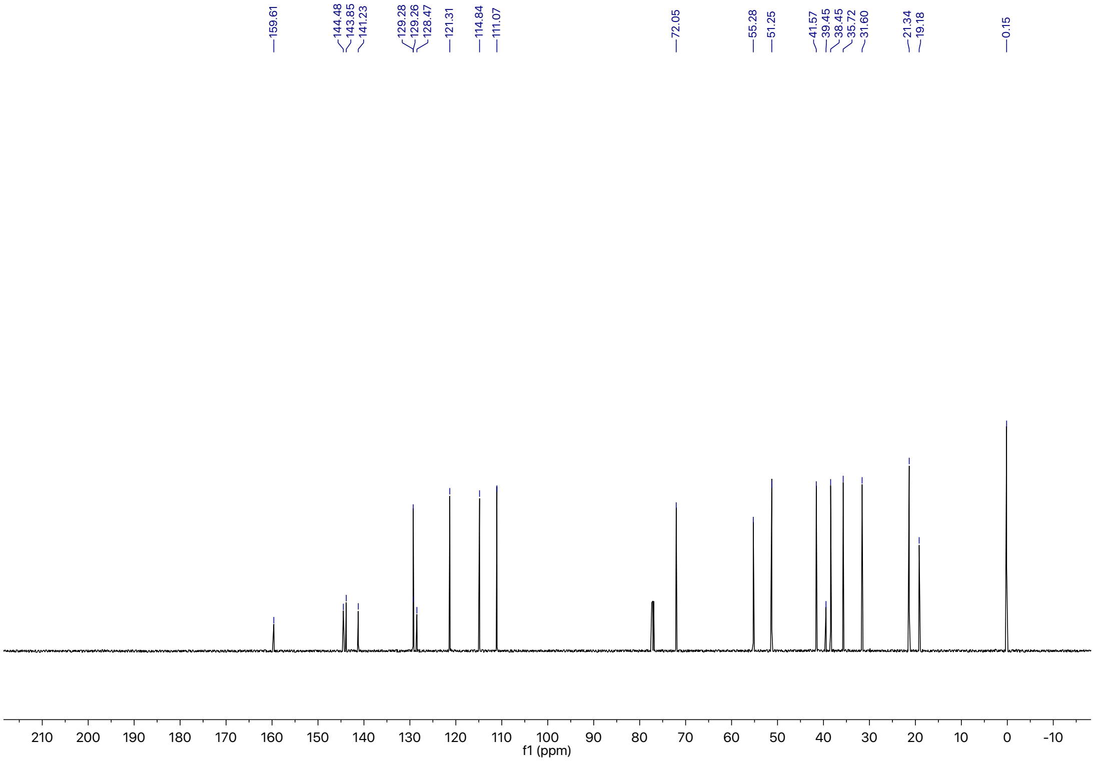

Supplementary Figure 11. ^{13}C NMR of **10**

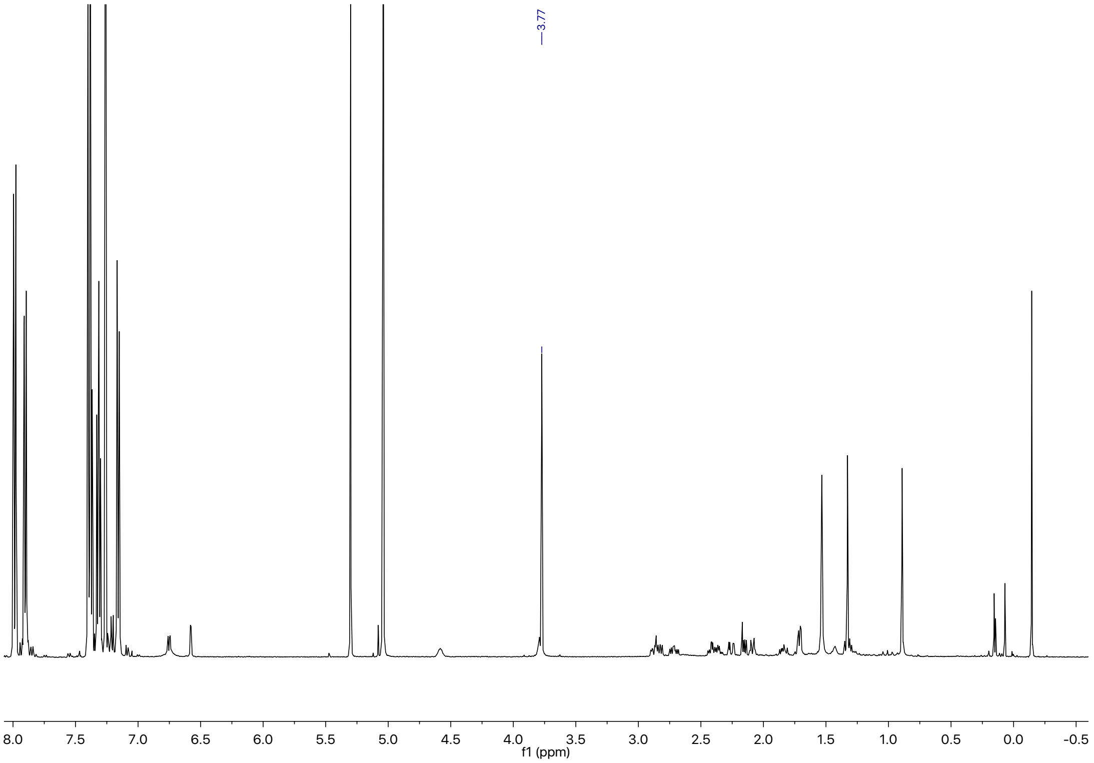

Supplementary Figure 12. Crude ^{1}H NMR of (*S*)-BINOL mediated cyclization

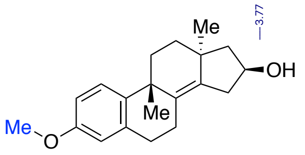

11

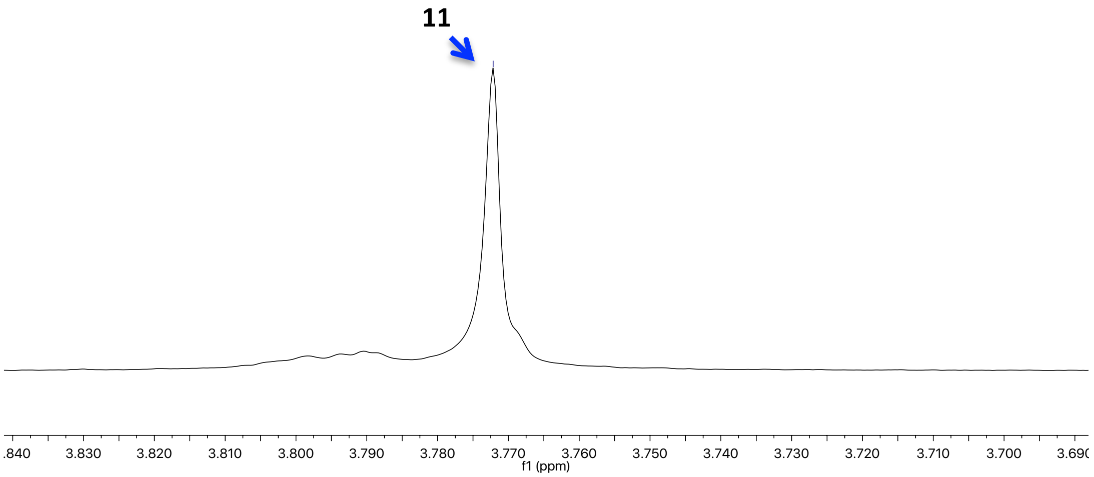

Supplementary Figure 13. Expanded Crude ^{1}H NMR of (*S*)-BINOL mediated cyclization

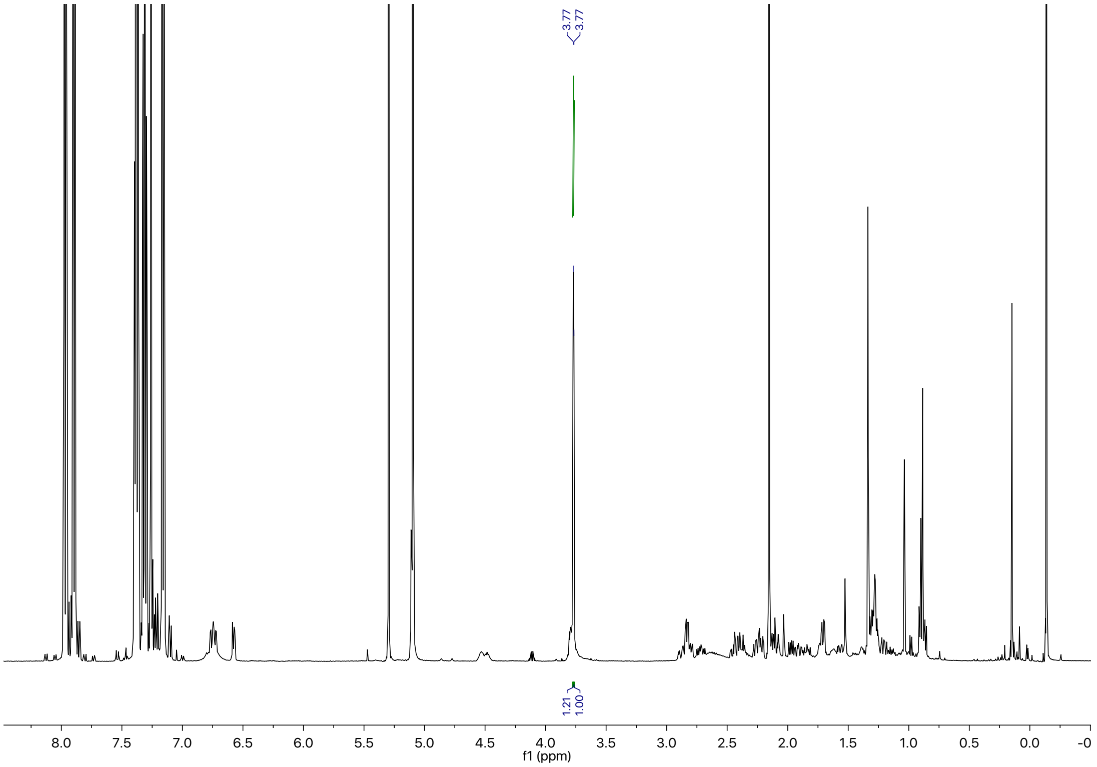

Supplementary Figure 14. Crude ^{1}H NMR of (*R*)-BINOL mediated cyclization

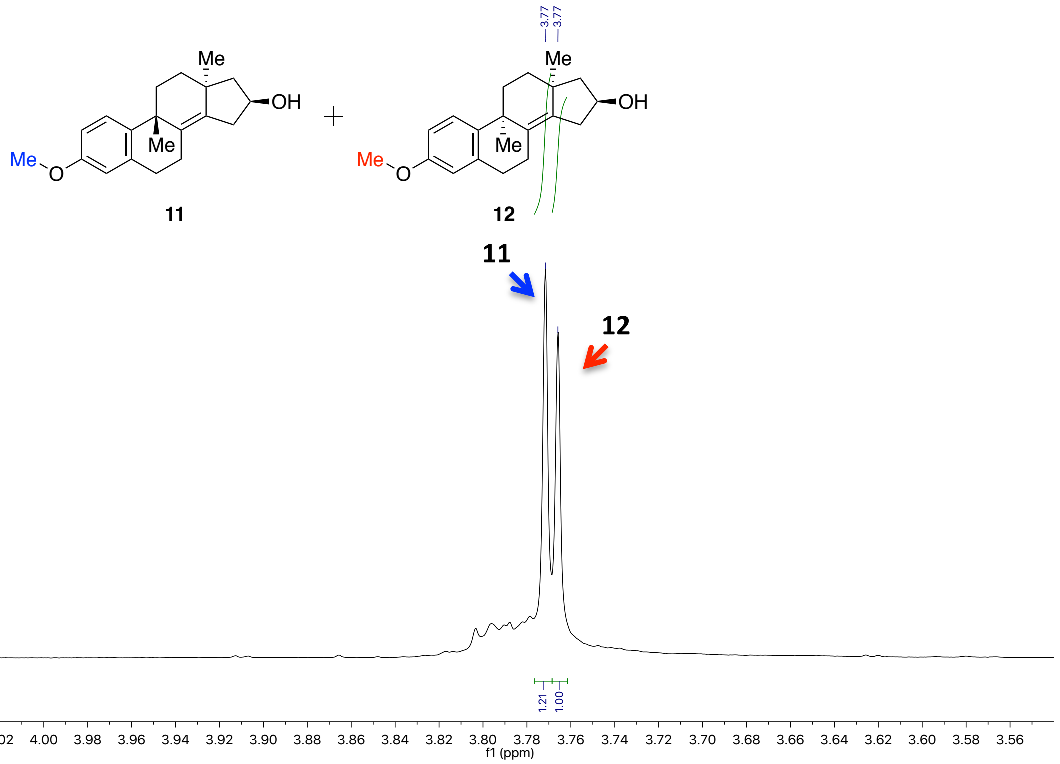

Supplementary Figure 15. Expanded Crude ^{1}H NMR of (*R*)-BINOL mediated cyclization

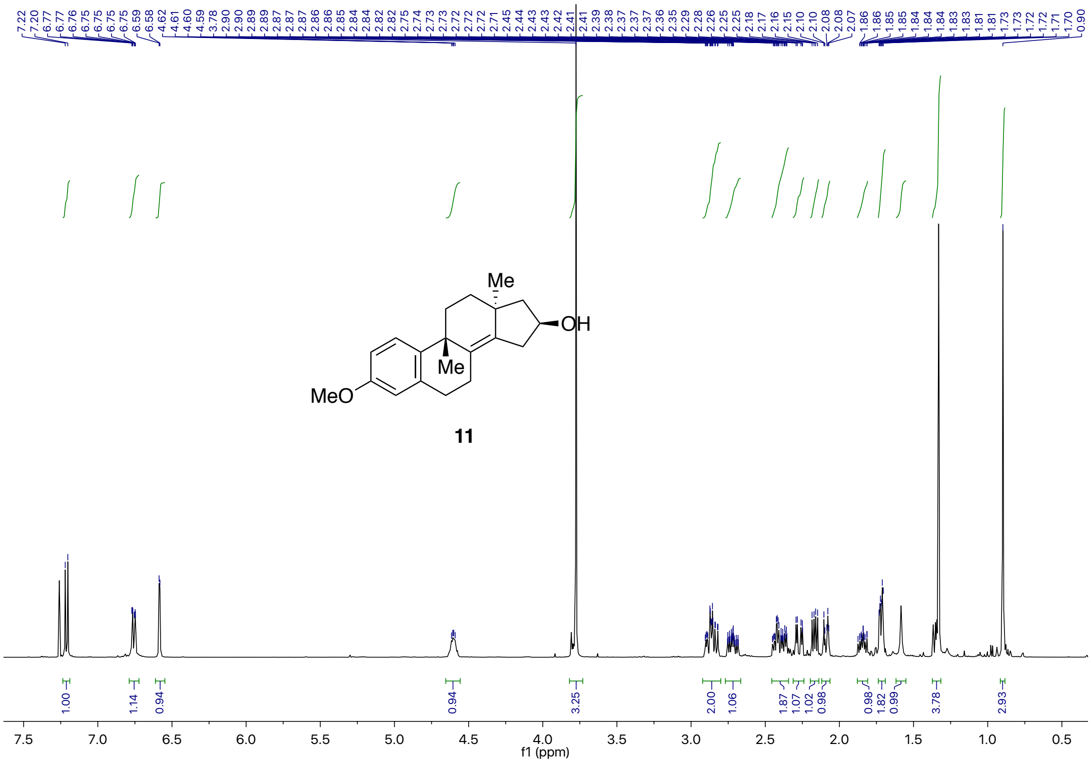

Supplementary Figure 16. ^{1}HNMR of **11**

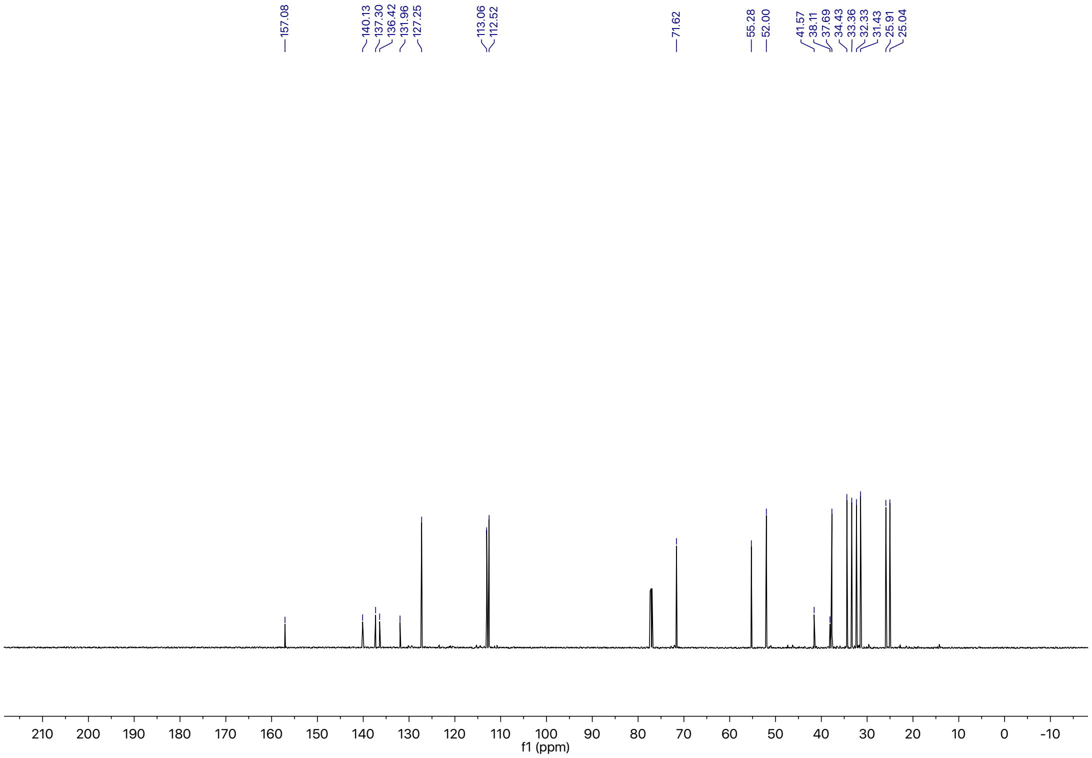

Supplementary Figure 17. ^{13}C NMR of **11**

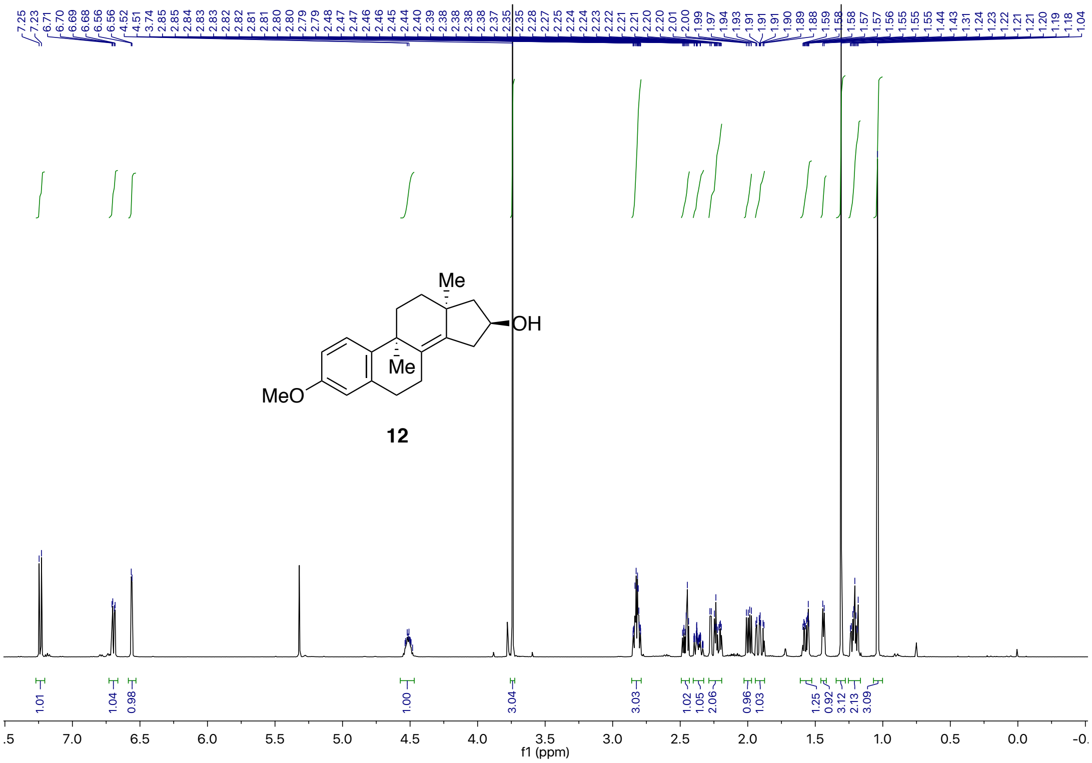

Supplementary Figure 18. ^{1}HNMR of **12**

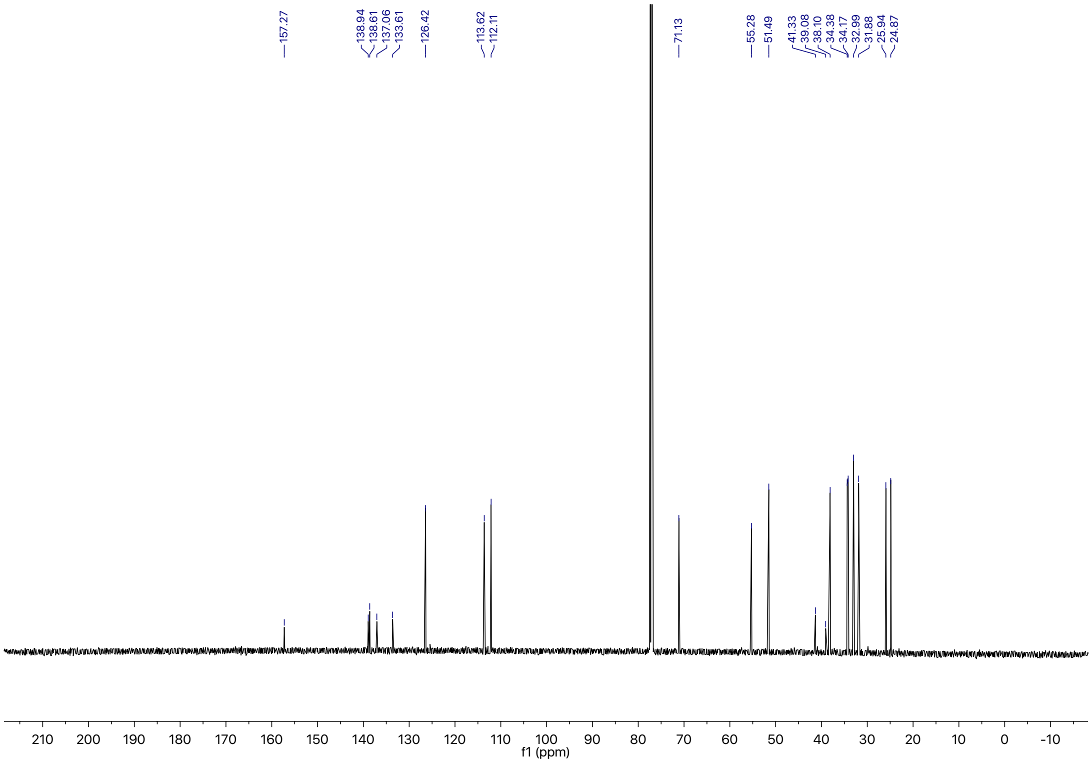

Supplementary Figure 19. ^{13}C NMR **12**

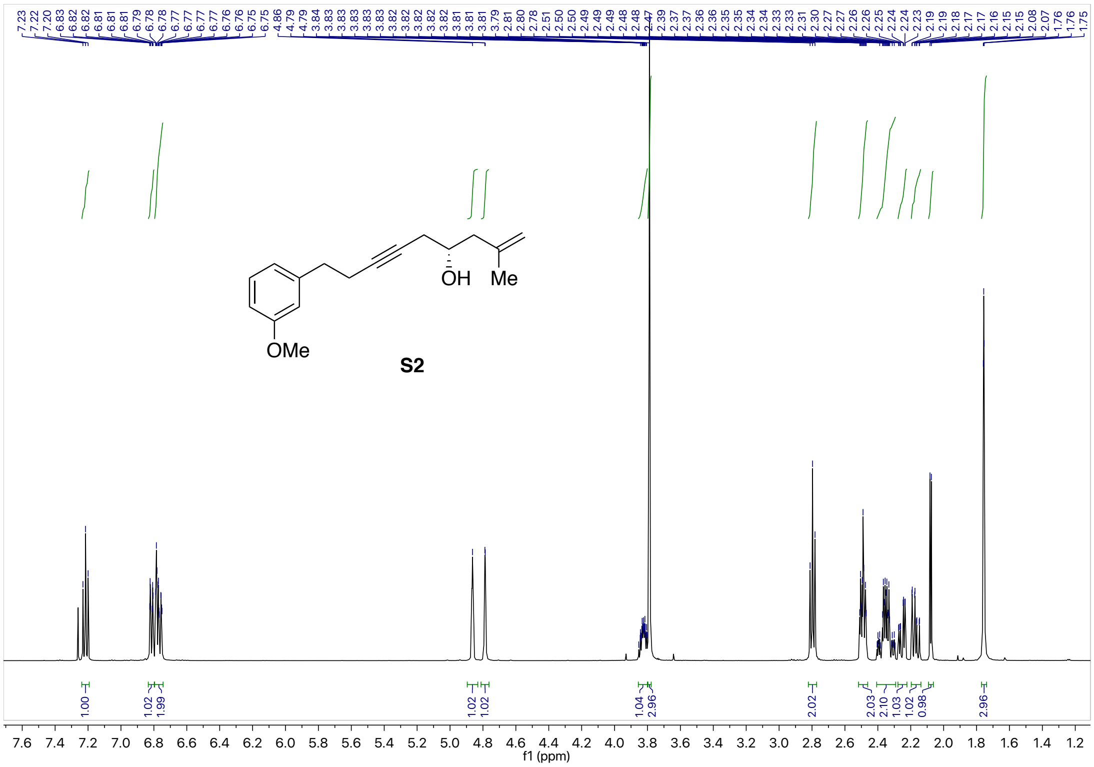

Supplementary Figure 20. ^{1}HNMR of **S2**

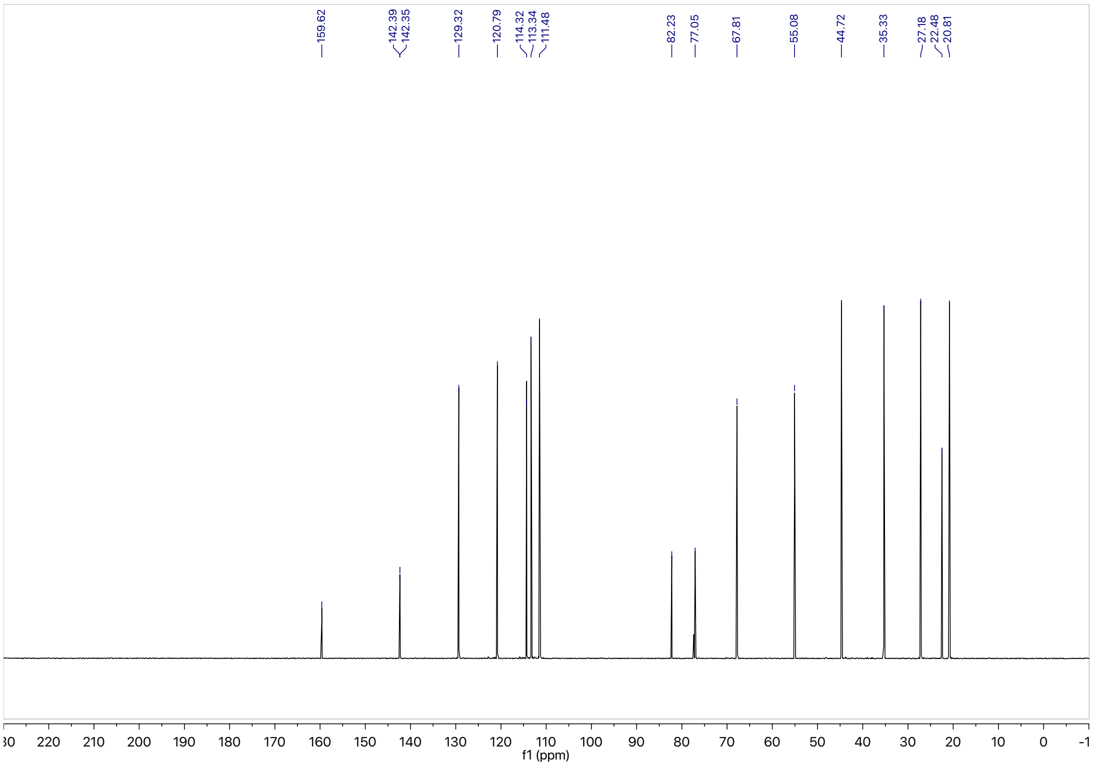

Supplementary Figure 21. ^{13}C NMR of **S2**

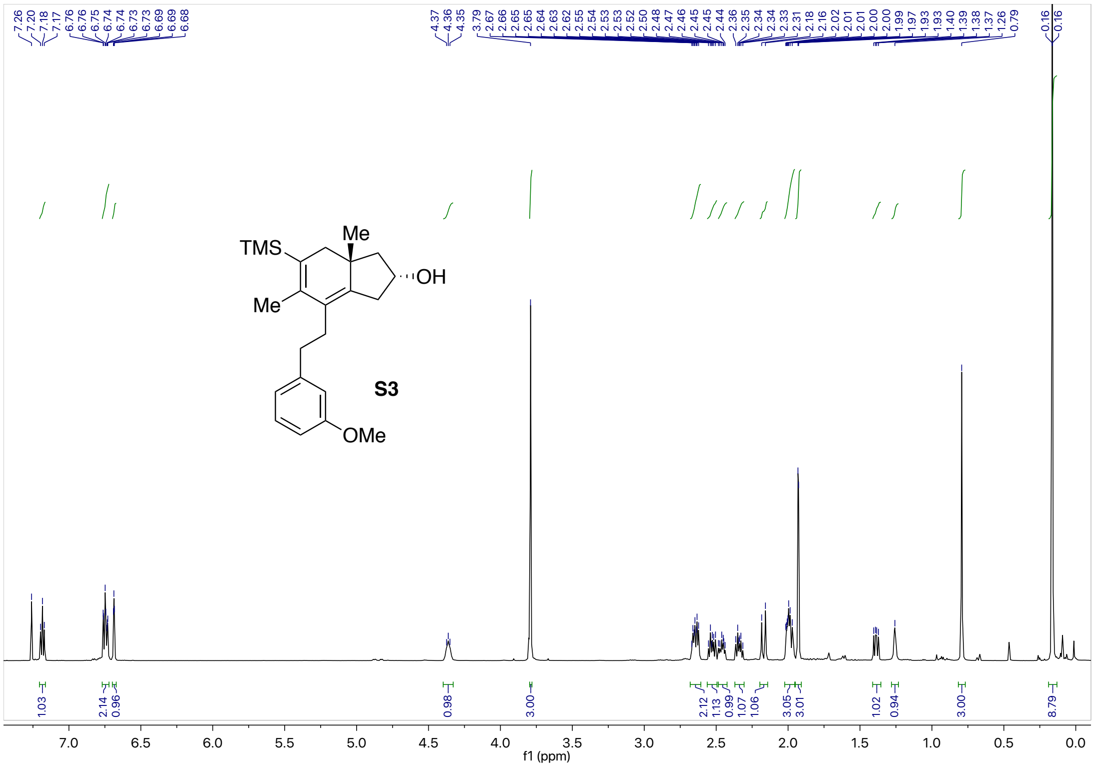

Supplementary Figure 22. ^{1}HNMR of **S3**

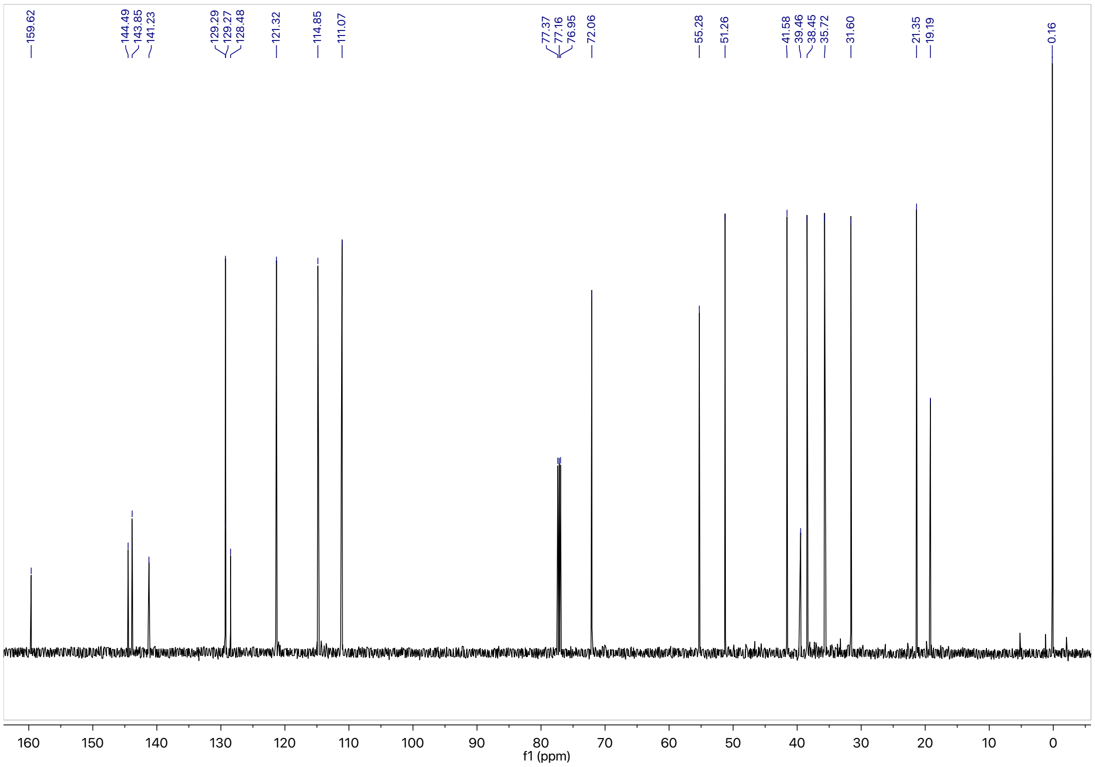

Supplementary Figure 23. ^{13}C NMR of **S3**

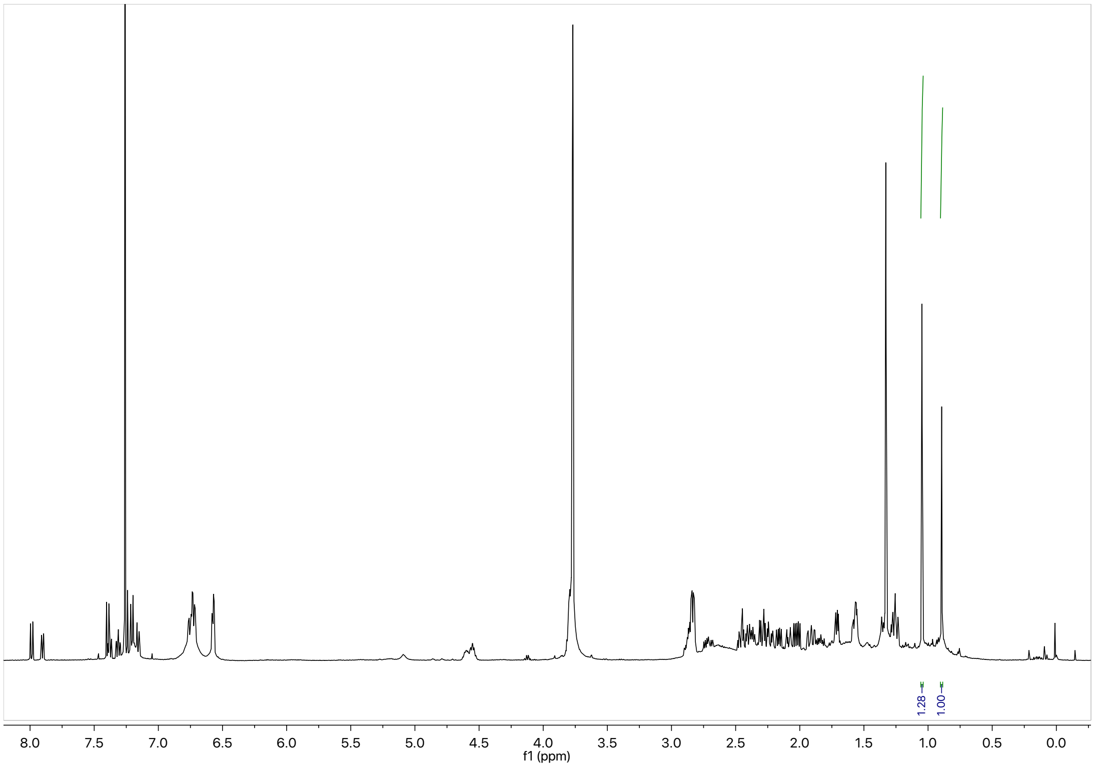

Supplementary Figure 24. Crude ^{1}H NMR of (S)-Binol / $SnCl_4$ cyclization of **S4**

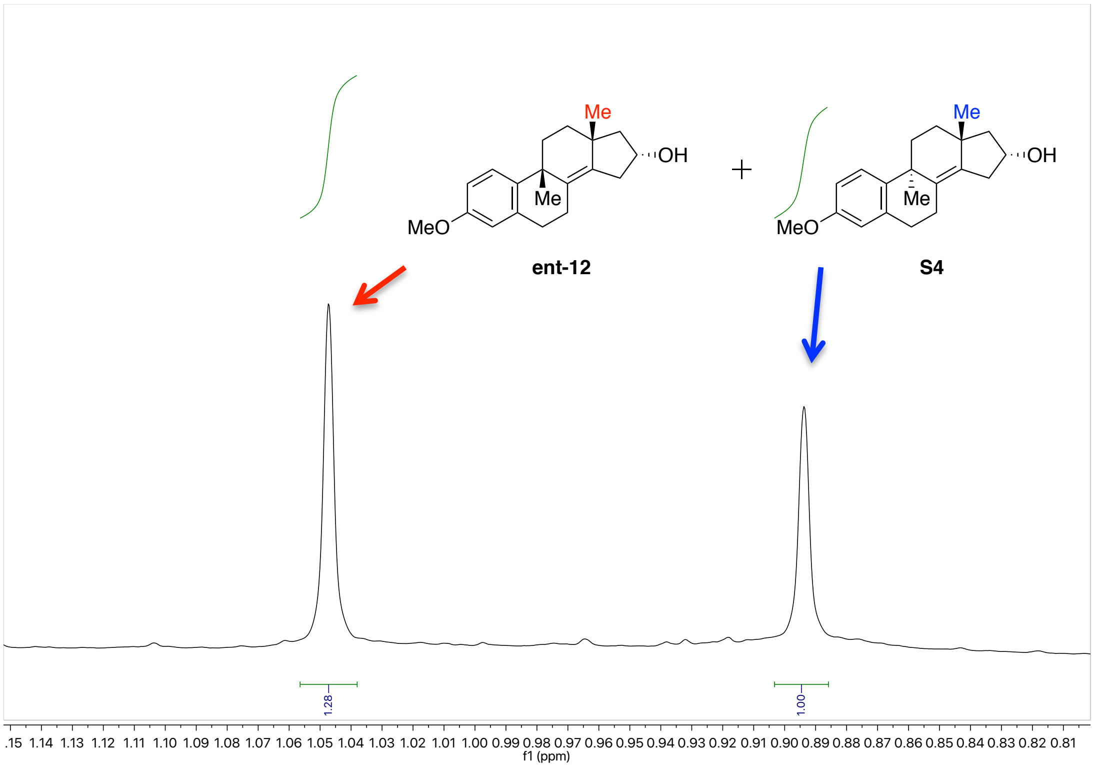

Supplementary Figure 25. Expanded Crude ^{1}H NMR of (S)-Binol / $SnCl_4$ cyclization of **S4**

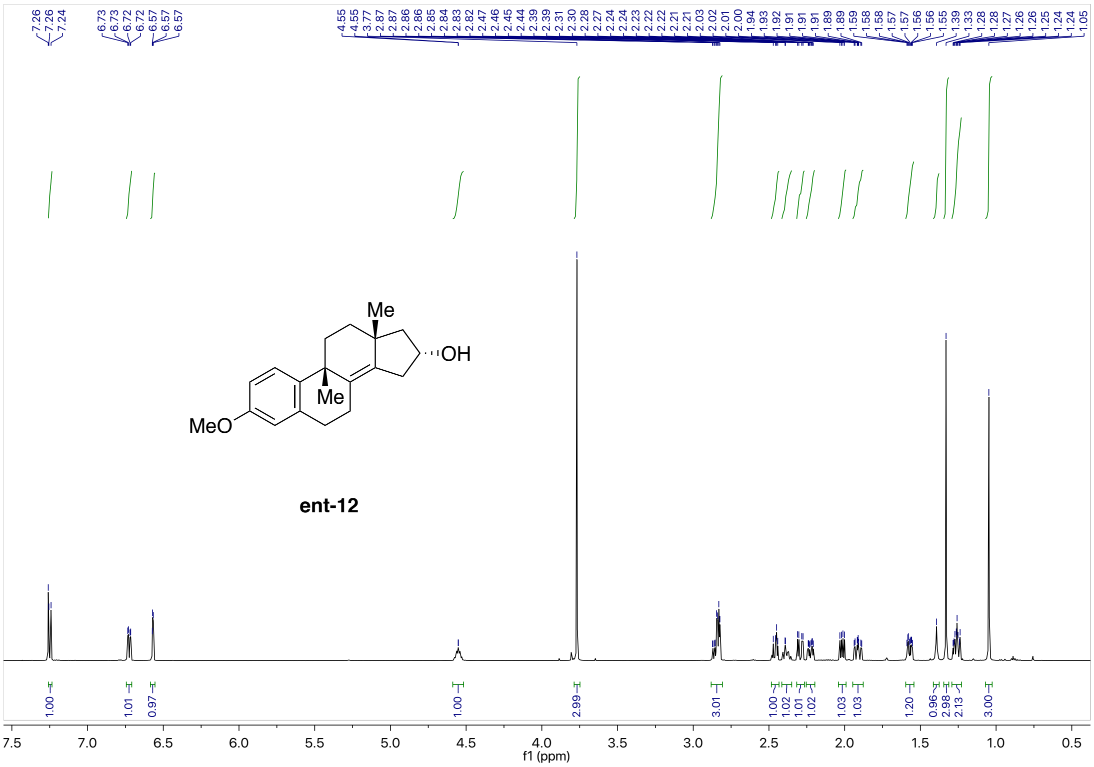

Supplementary Figure 26. ^{1}HNMR of **ent-12**

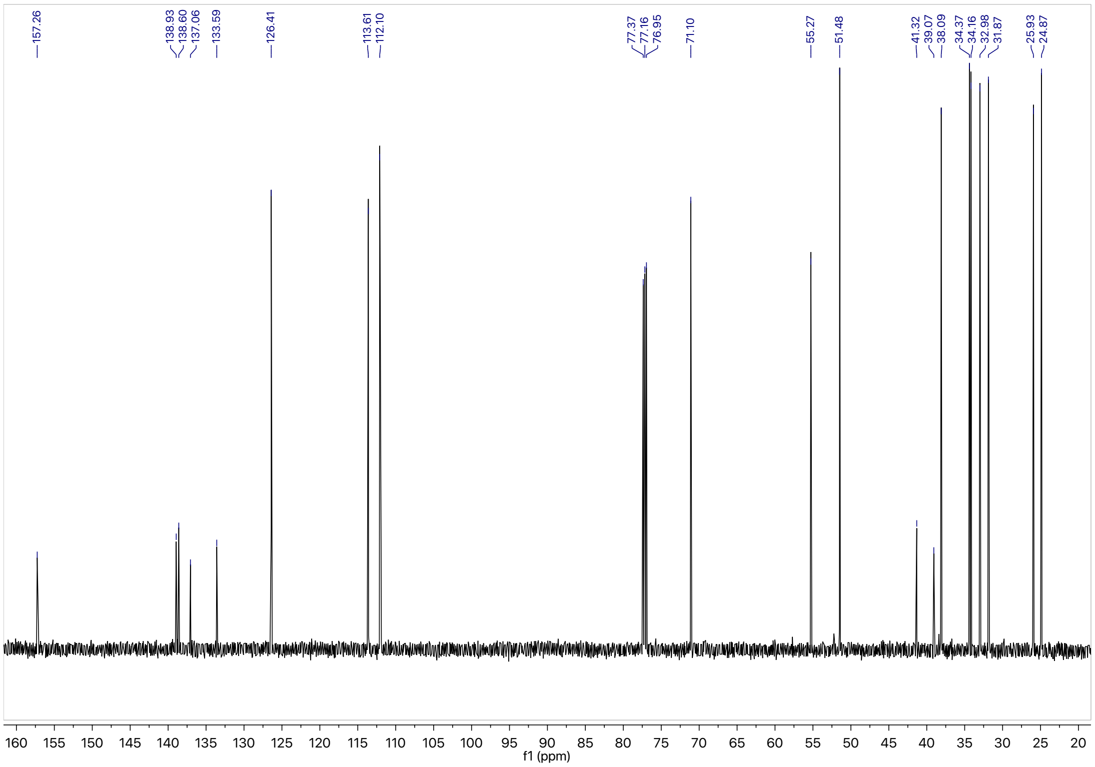

Supplementary Figure 27. ^{13}C NMR of ent-**12**

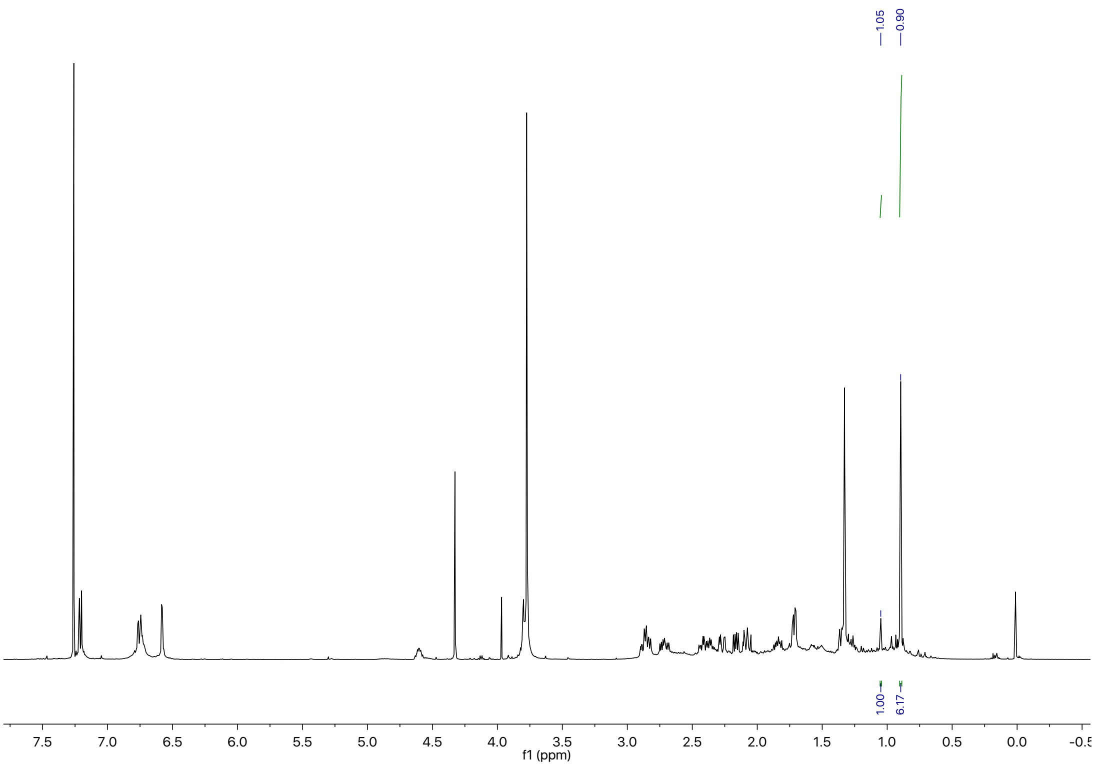

Supplementary Figure 28. Crude ^{1}H NMR of BF_3•OEt_2 Cyclization

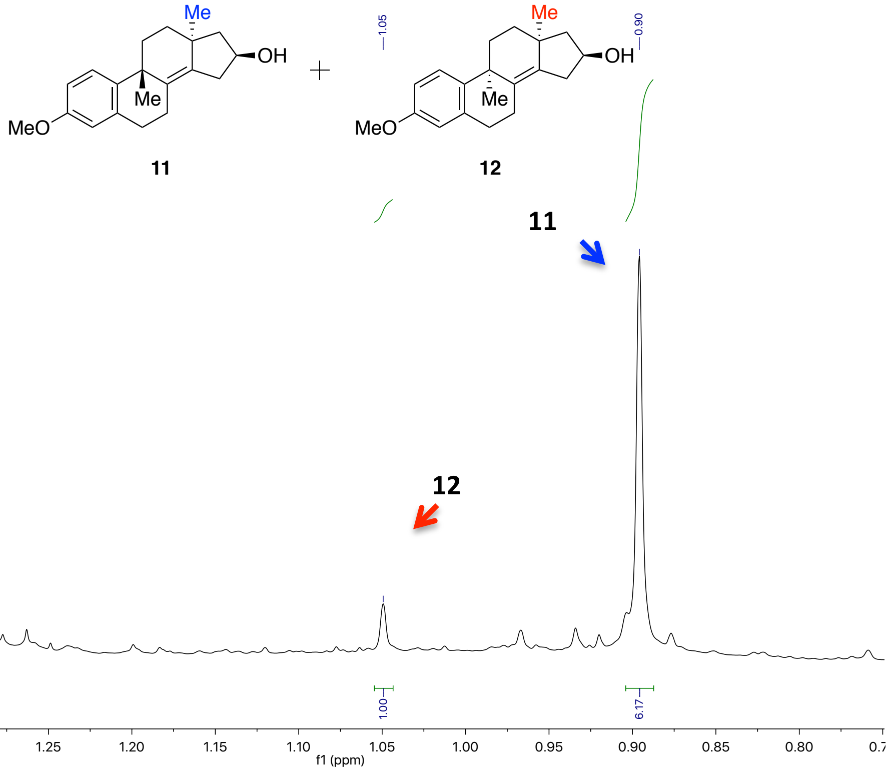

Supplementary Figure 29. Expanded Crude ^{1}H NMR of BF_3•OEt_2 Cyclization

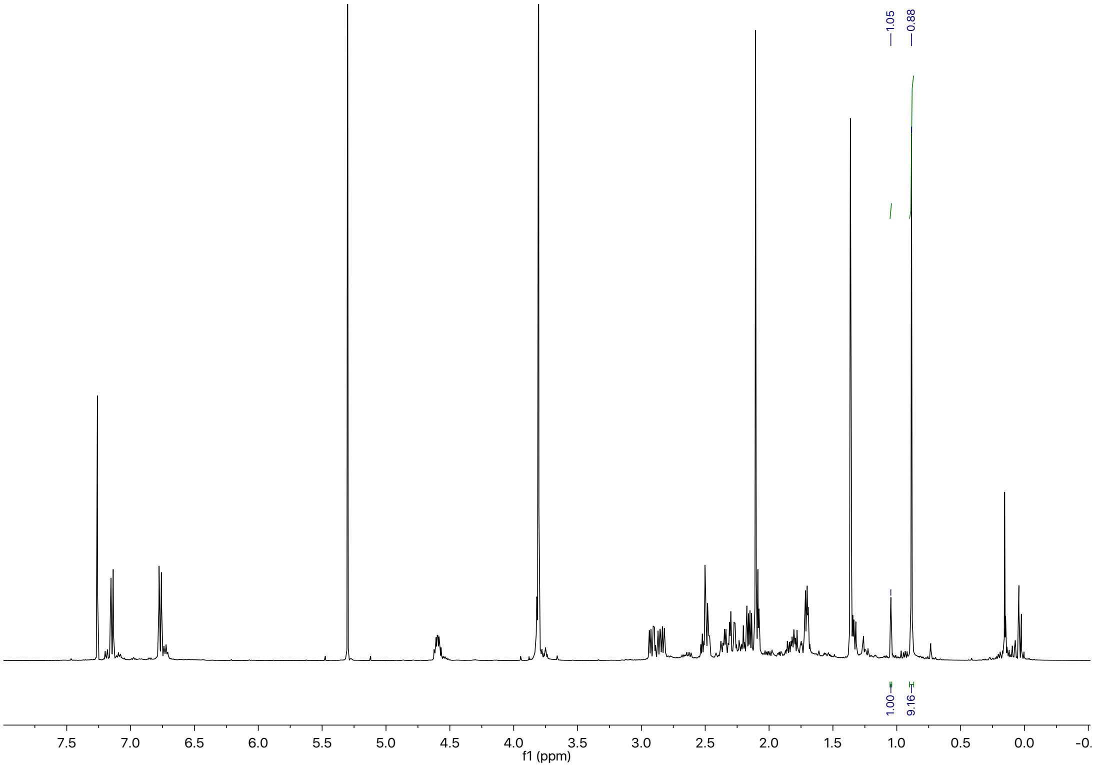

Supplementary Figure 30. Crude ^{1}H NMR of *o,o'*–dihydroxybiphenyl / $SnCl_4$ cyclization

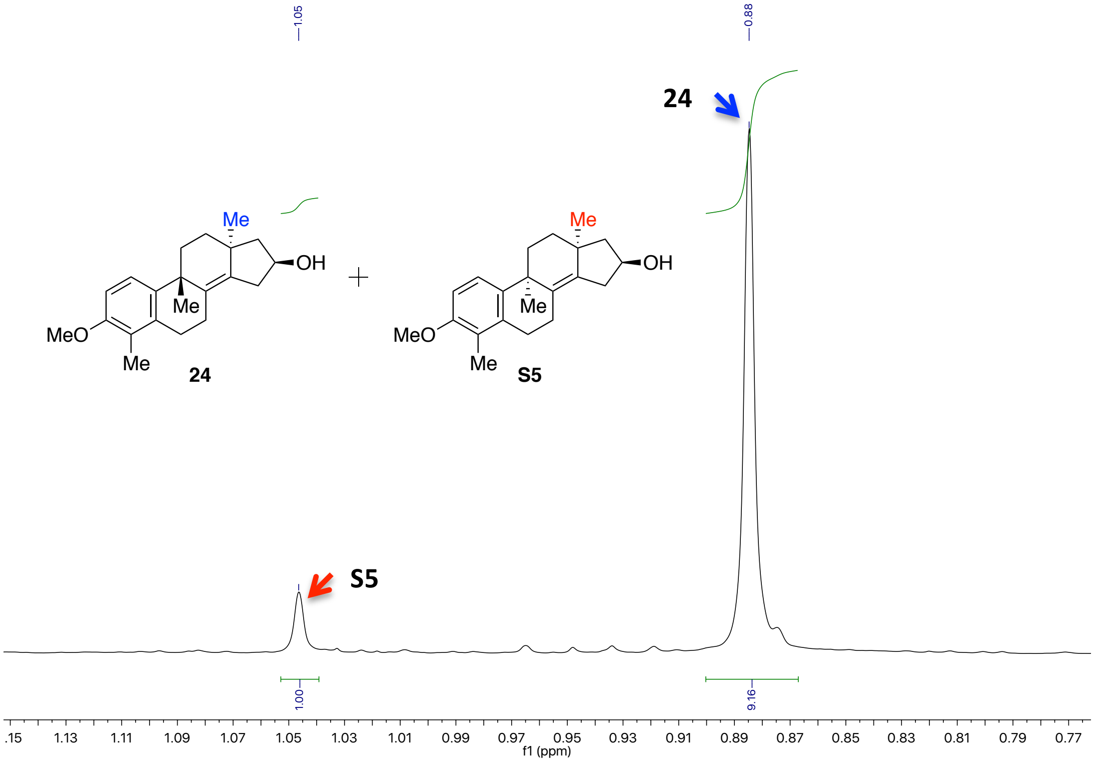

Supplementary Figure 31. Expanded Crude ^{1}H NMR of *o,o'*–dihydroxybiphenyl / $SnCl_4$ cyclization

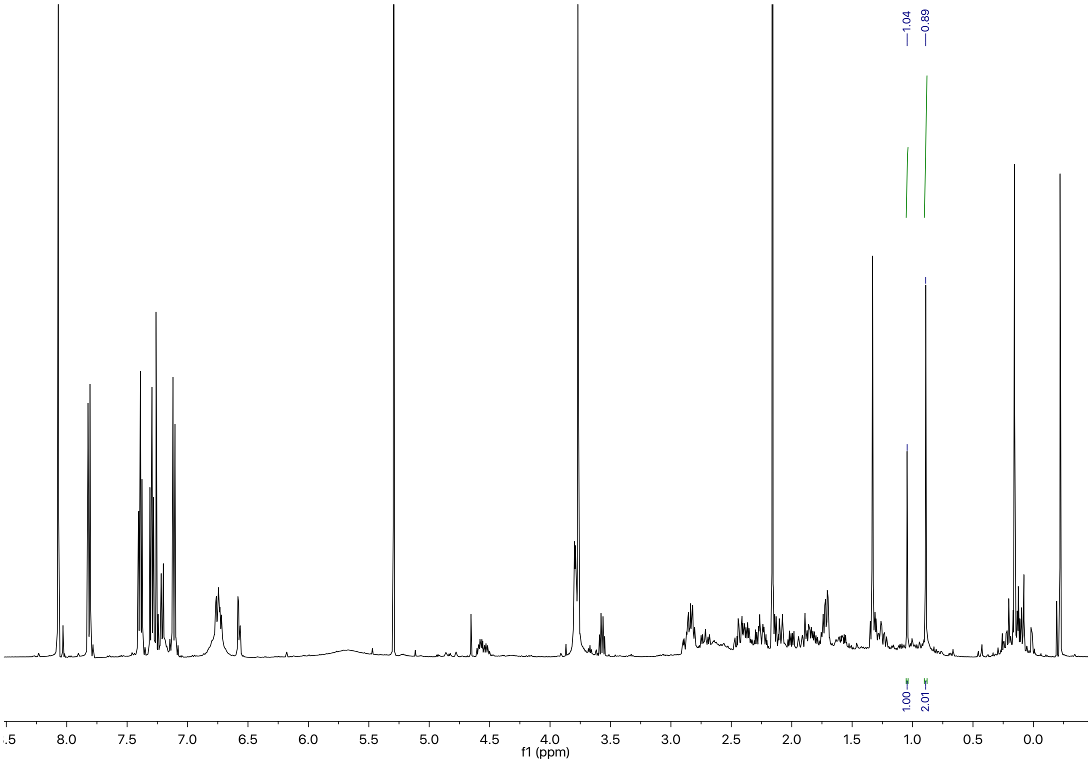

Supplementary Figure 32. Crude ^{1}H NMR of (*R*)–*o*,*o*'–Cl_2BINOL / $SnCl_4$ cyclization

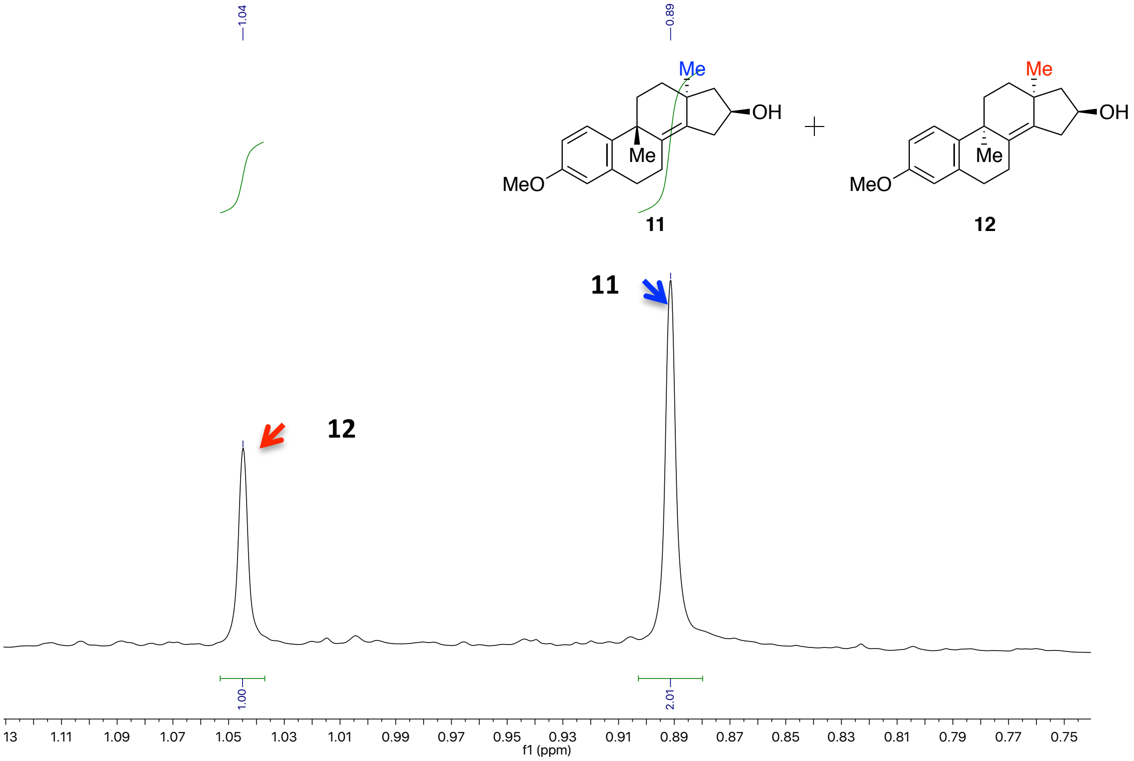

Supplementary Figure 33. Expanded Crude ^{1}H NMR of (*R*)–*o*,*o*'–Cl_2BINOL / $SnCl_4$ cyclization

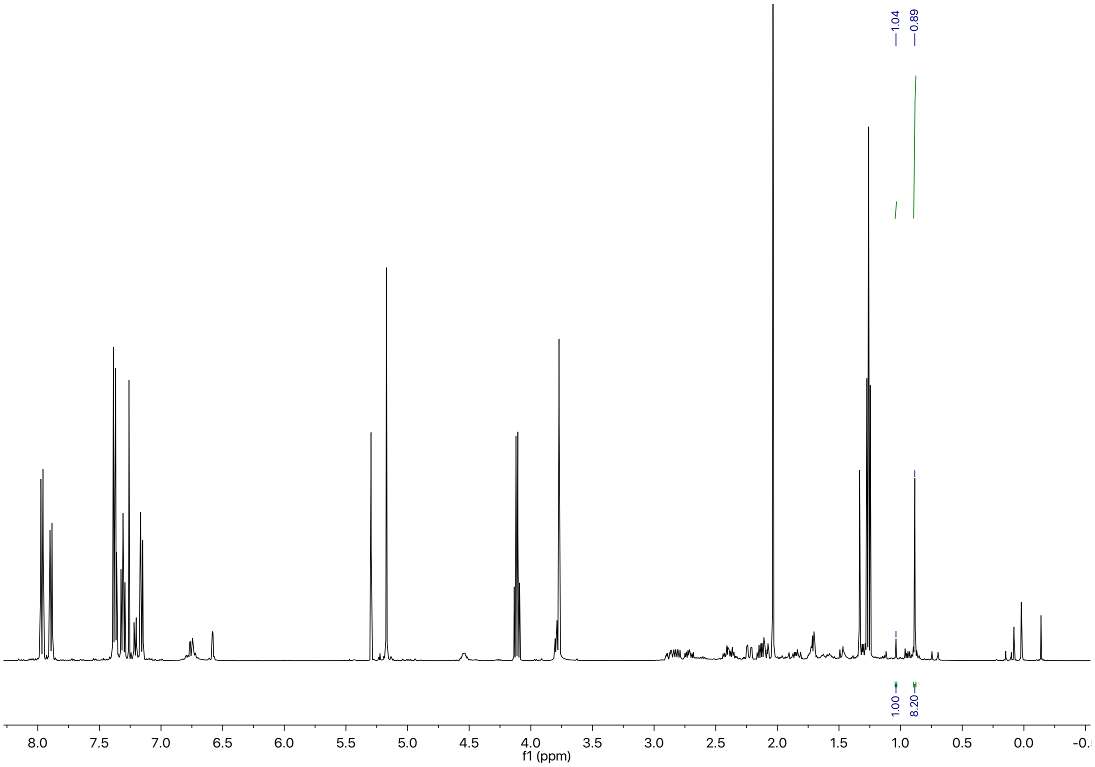

Supplementary Figure 34. Crude ^{1}H NMR of (*R*)–BINOL / $SbCl_5$ cyclization

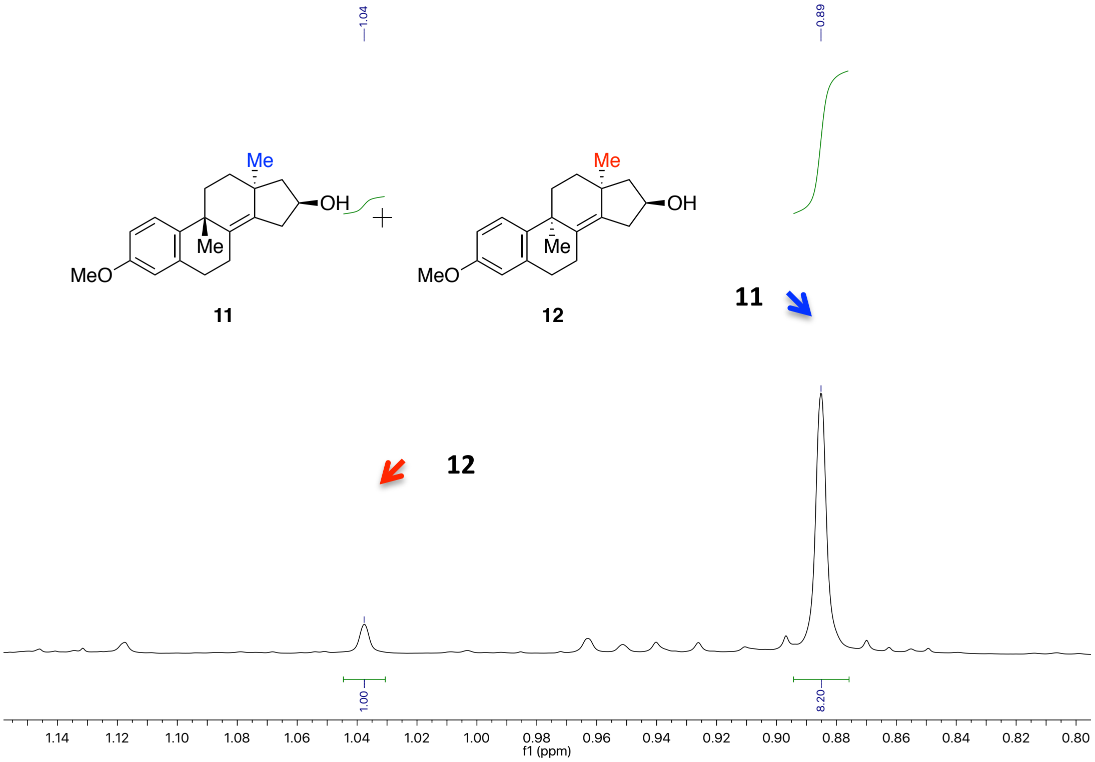

Supplementary Figure 35. Expanded Crude ^{1}H NMR of (*R*)–BINOL / $SbCl_5$ cyclization

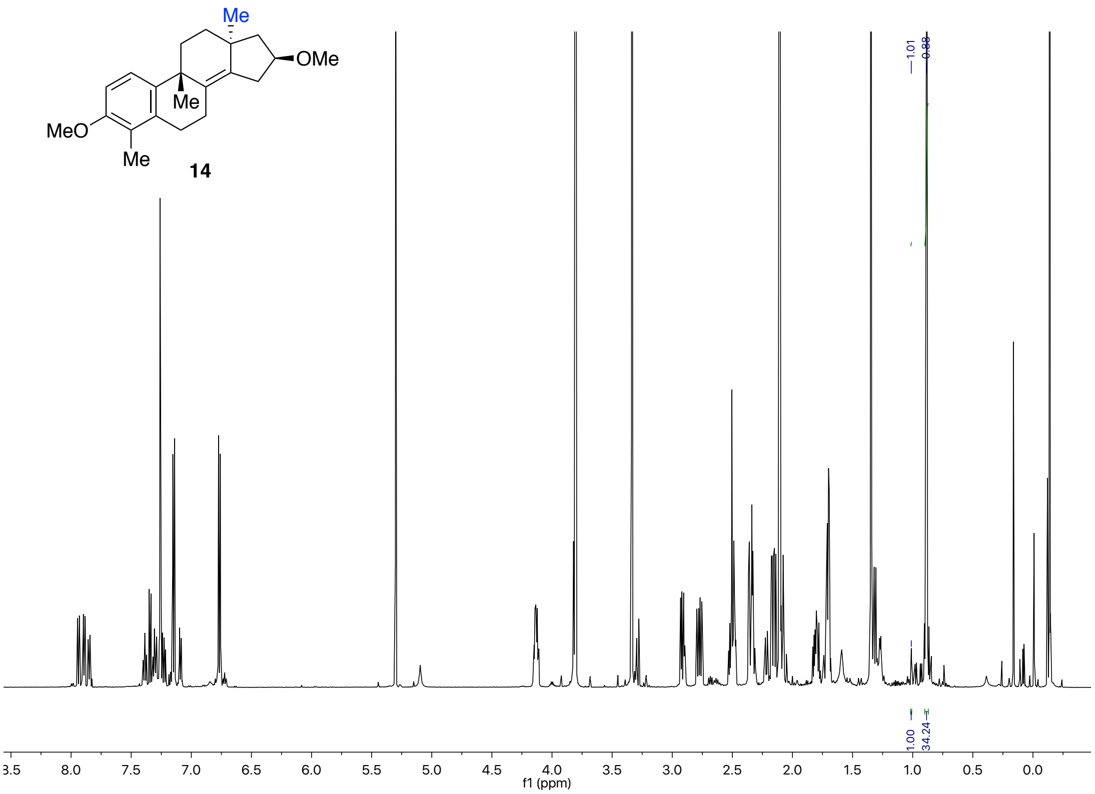

Supplementary Figure 36. Crude ^{1}H NMR of **14**

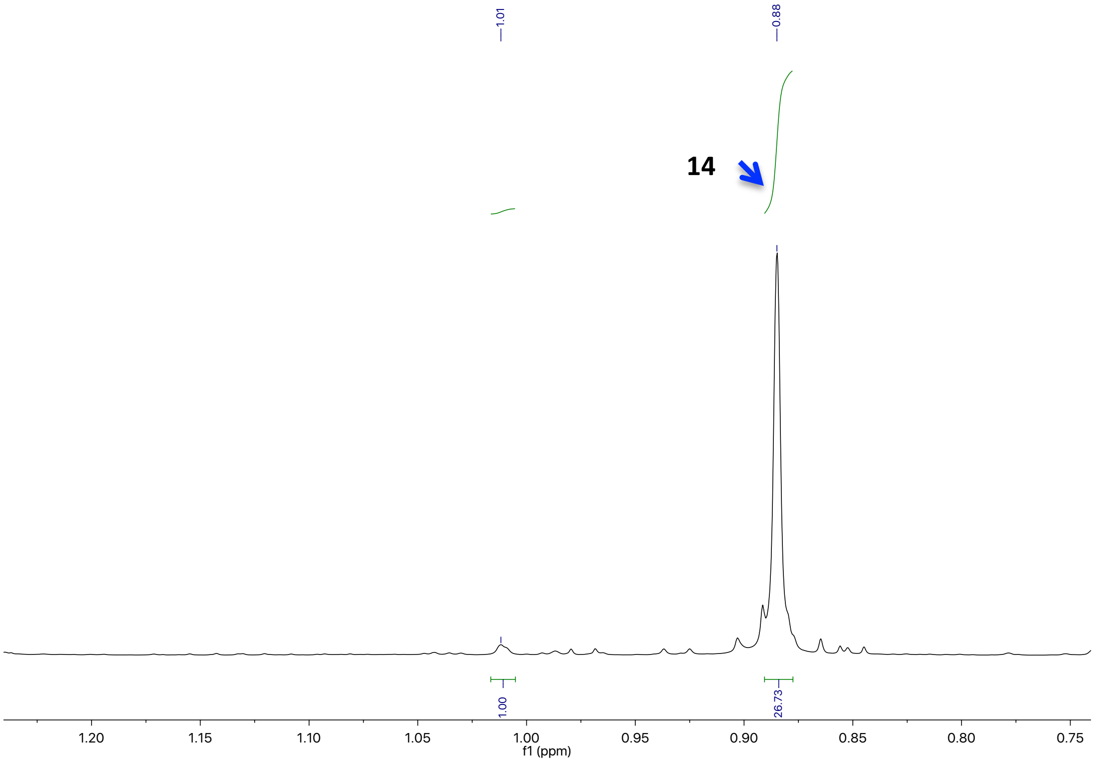

Supplementary Figure 37. Expanded Crude ^{1}H NMR of **14**

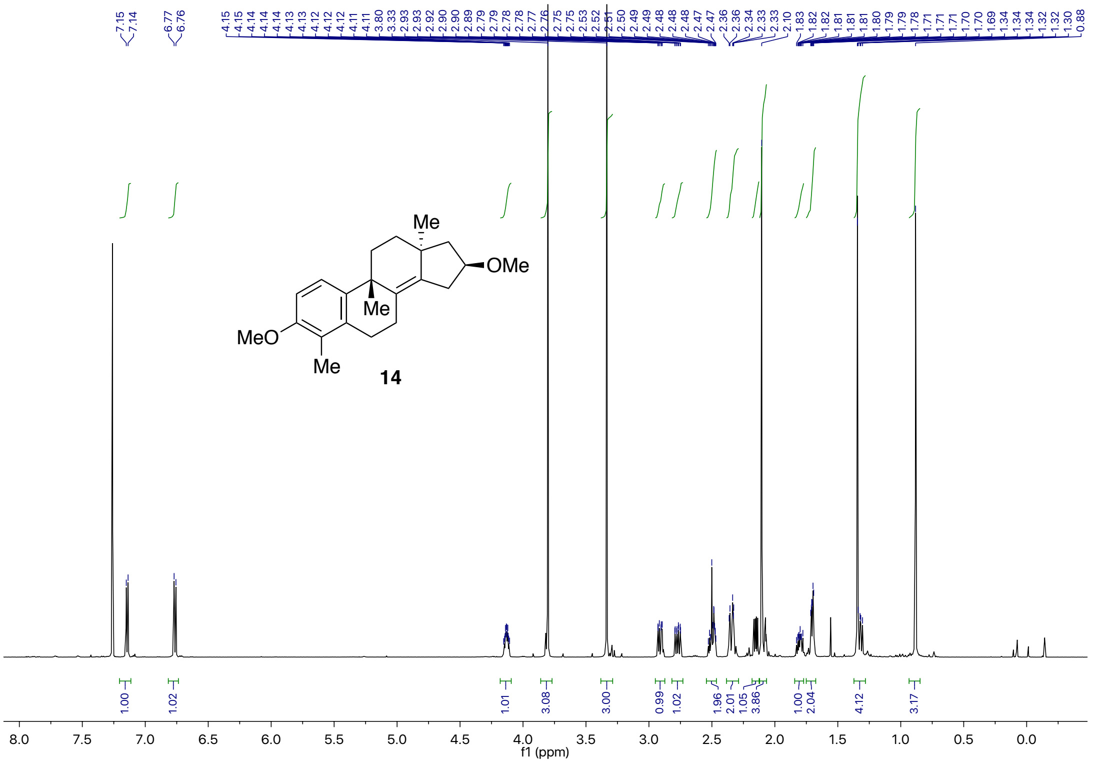

Supplementary Figure 38. ^{1}H NMR of **14**

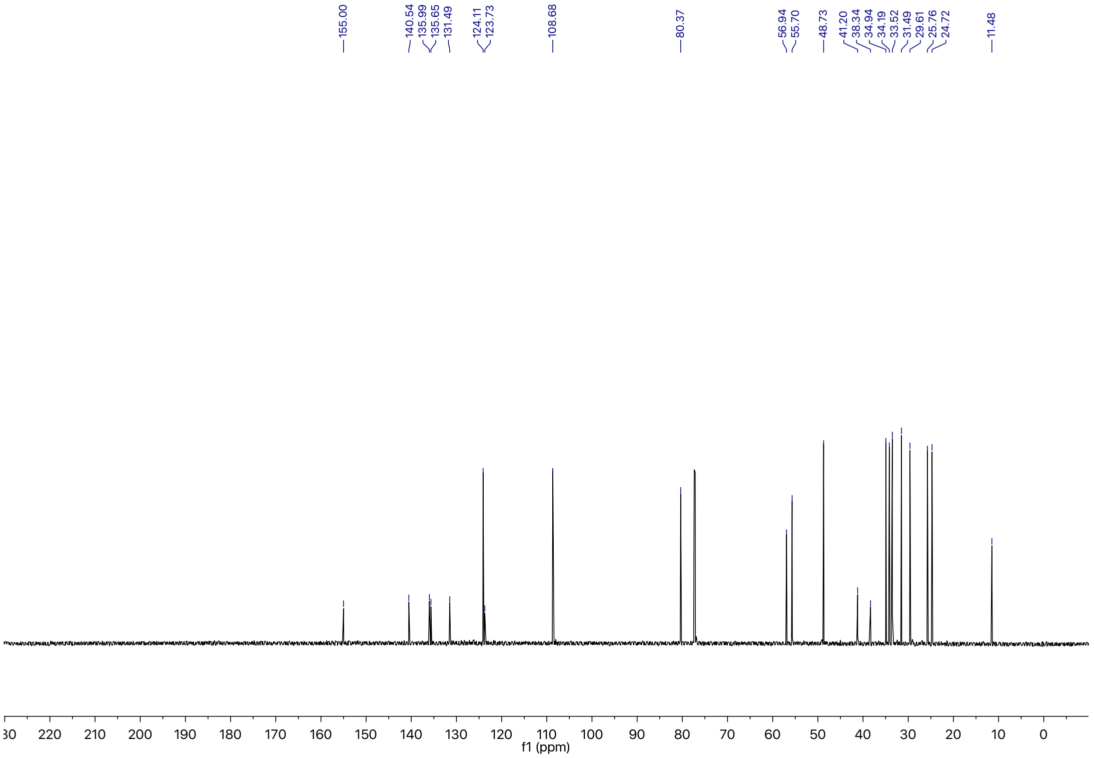

Supplementary Figure 39. ^{13}C NMR of **14**

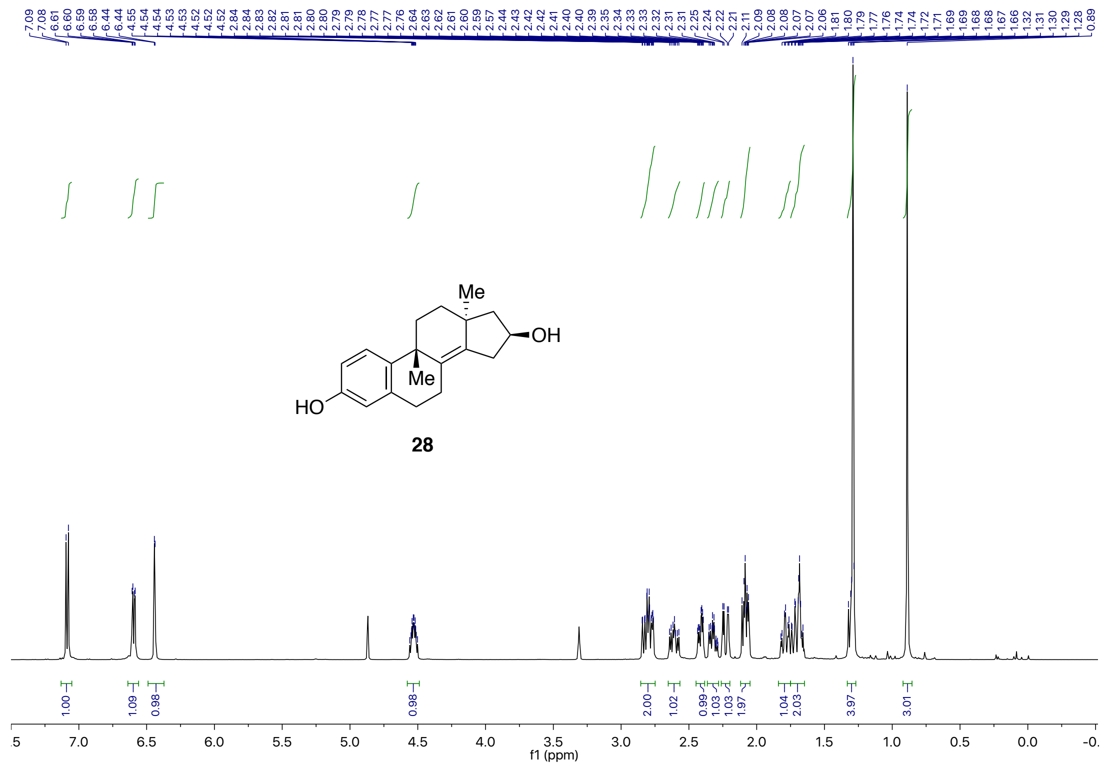

Supplementary Figure 40. ^{1}H NMR of **28**

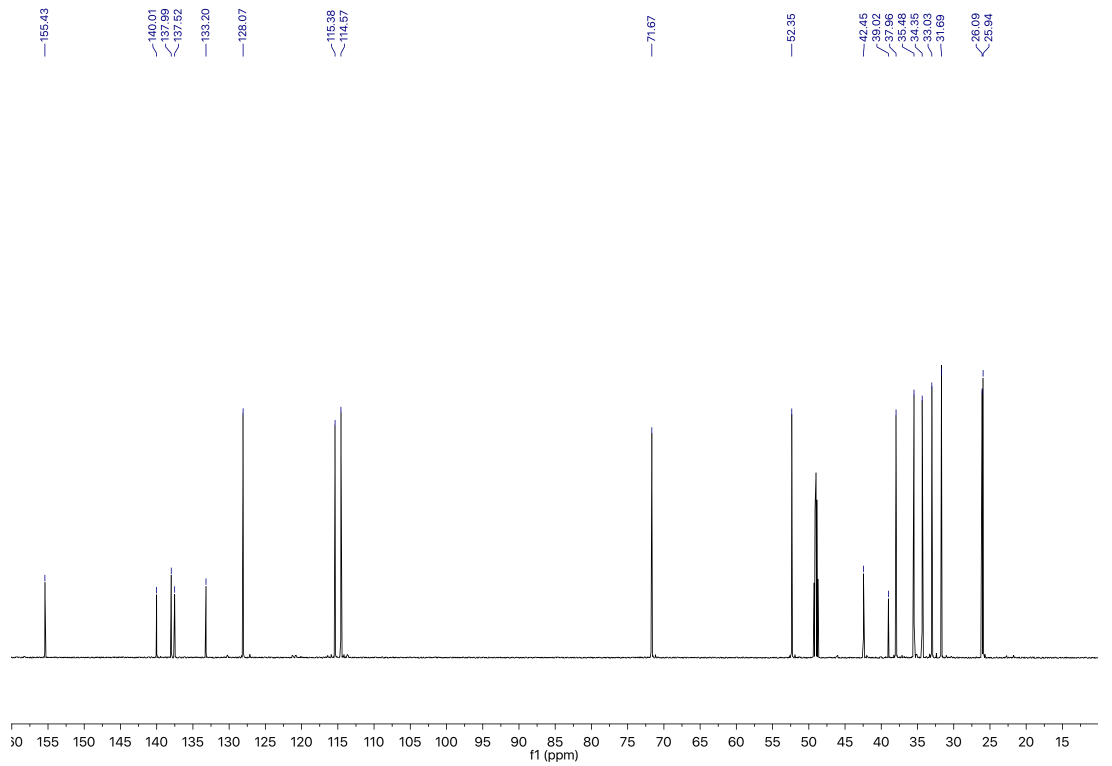

Supplementary Figure 41. ^{13}C NMR of **28**

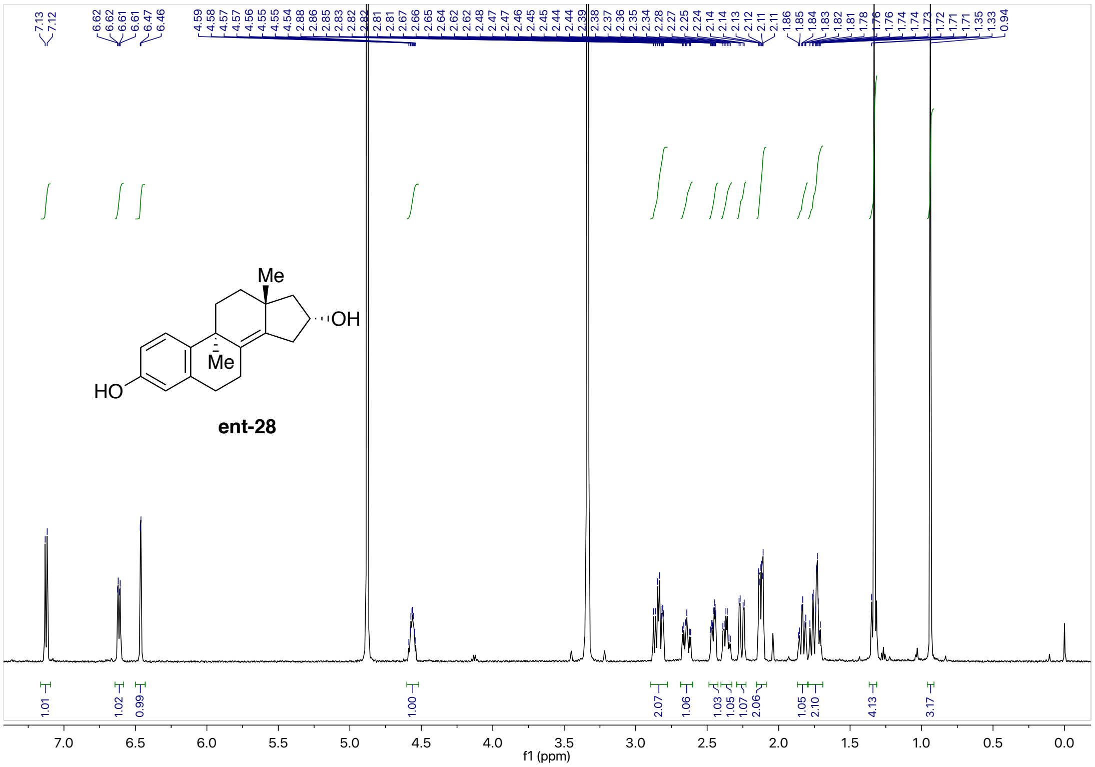

Supplementary Figure 42. ^{1}H NMR of **ent-28**

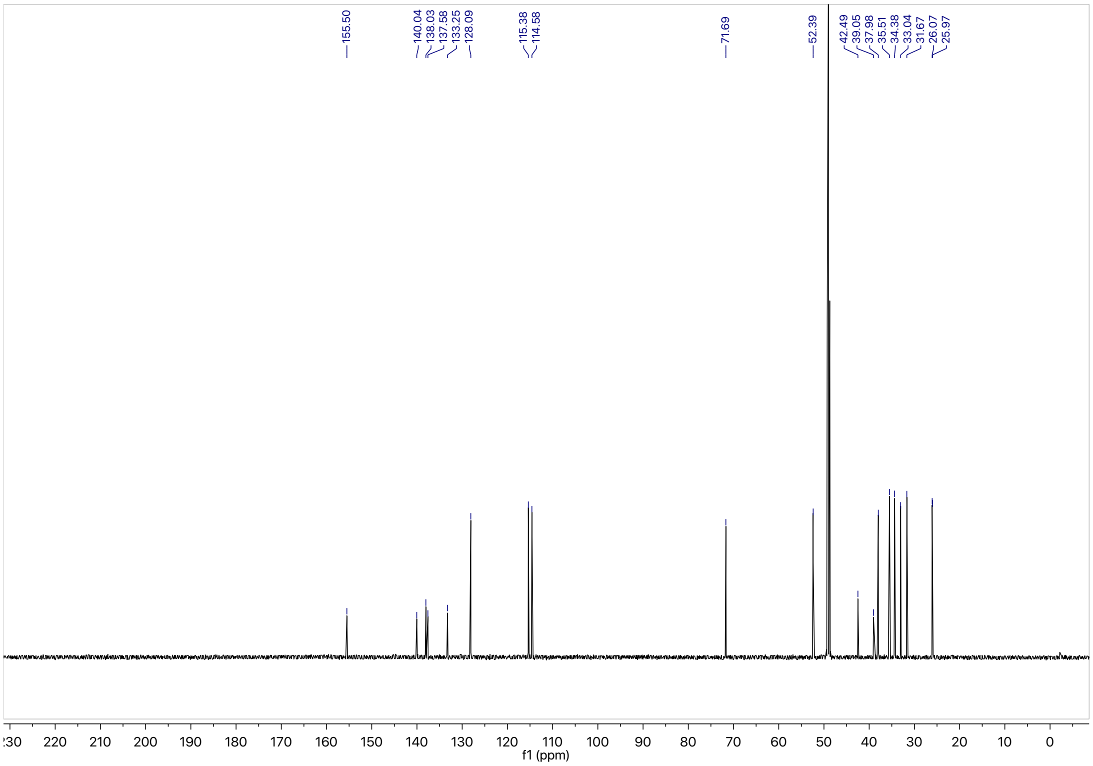

Supplementary Figure 43. ^{13}C NMR of ent-**28**

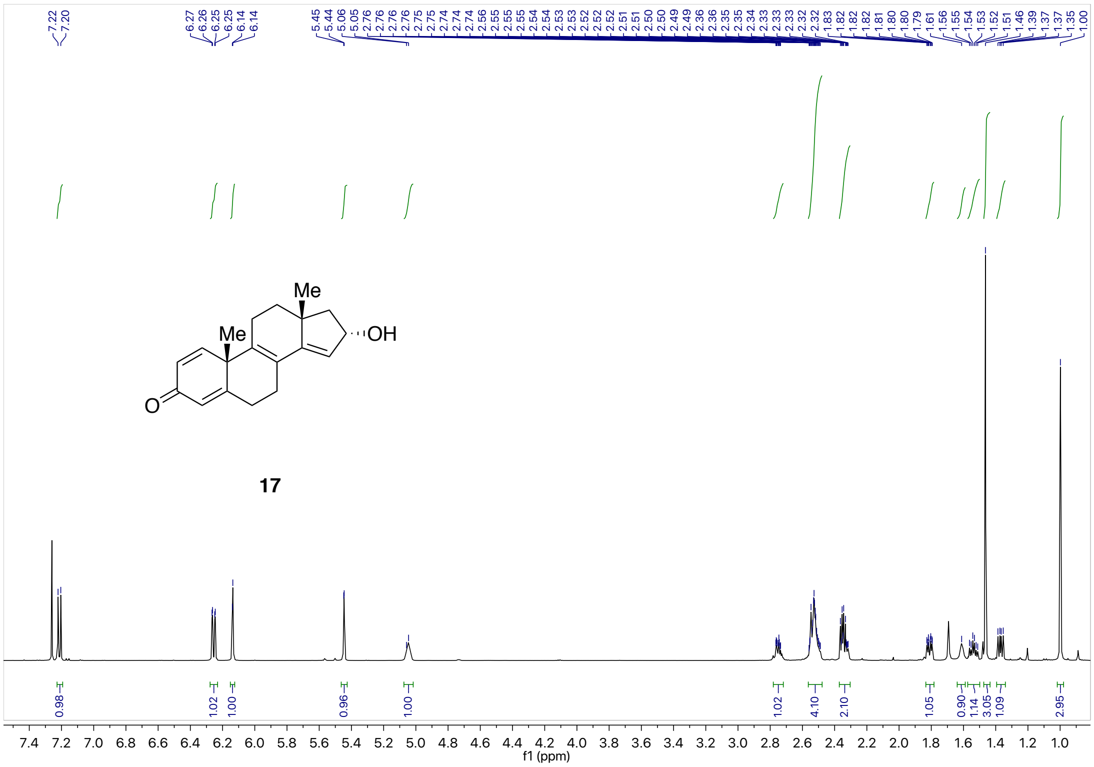

Supplementary Figure 44. ^{1}H NMR of **17**

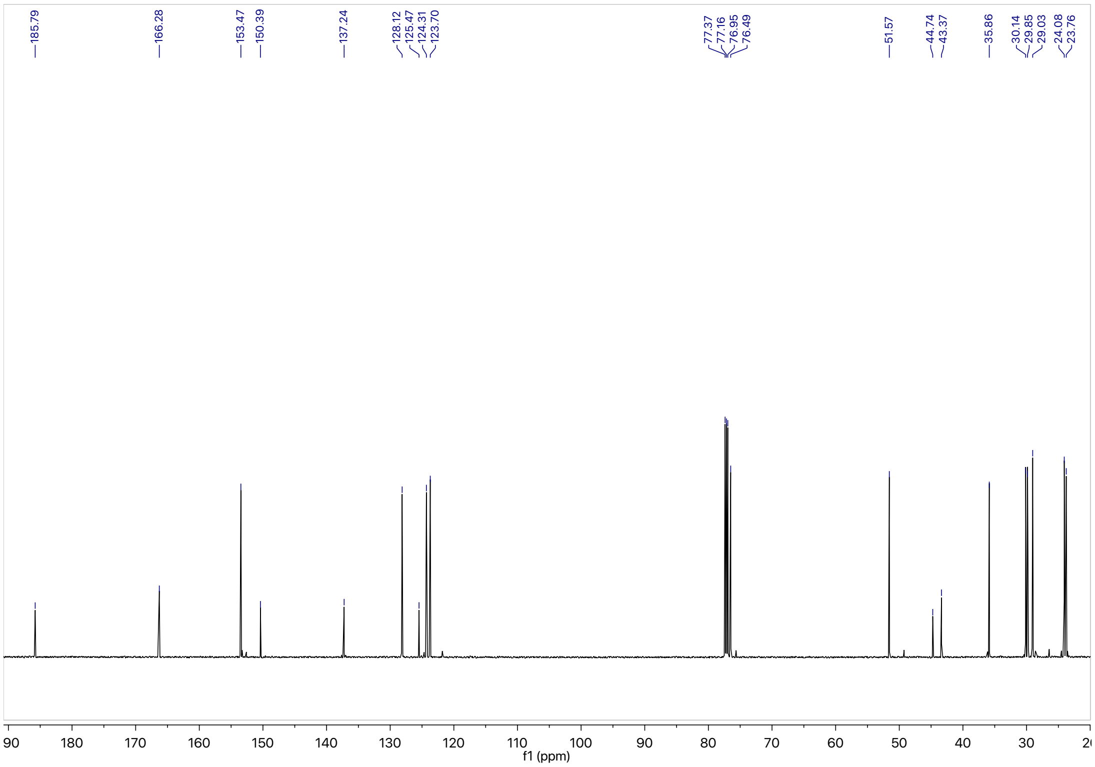

Supplementary Figure 45. ^{13}C NMR of **17**

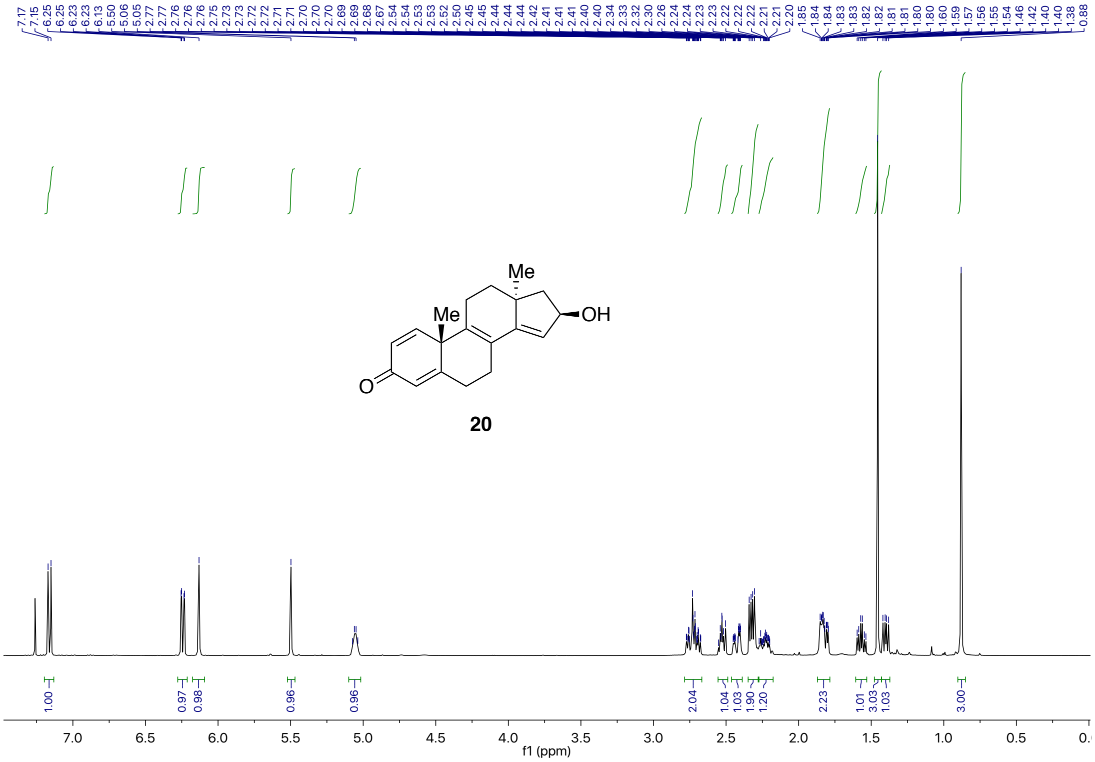

Supplementary Figure 46. ^{1}H NMR of **20**

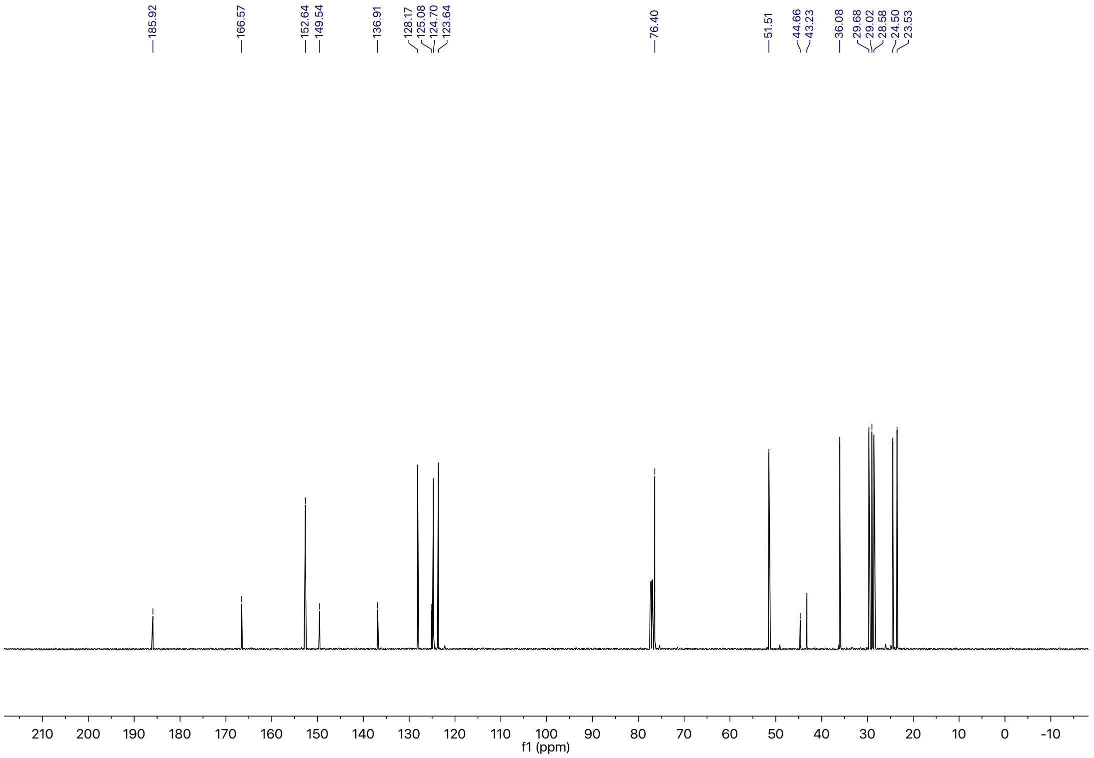

Supplementary Figure 47. ^{13}C NMR of **20**

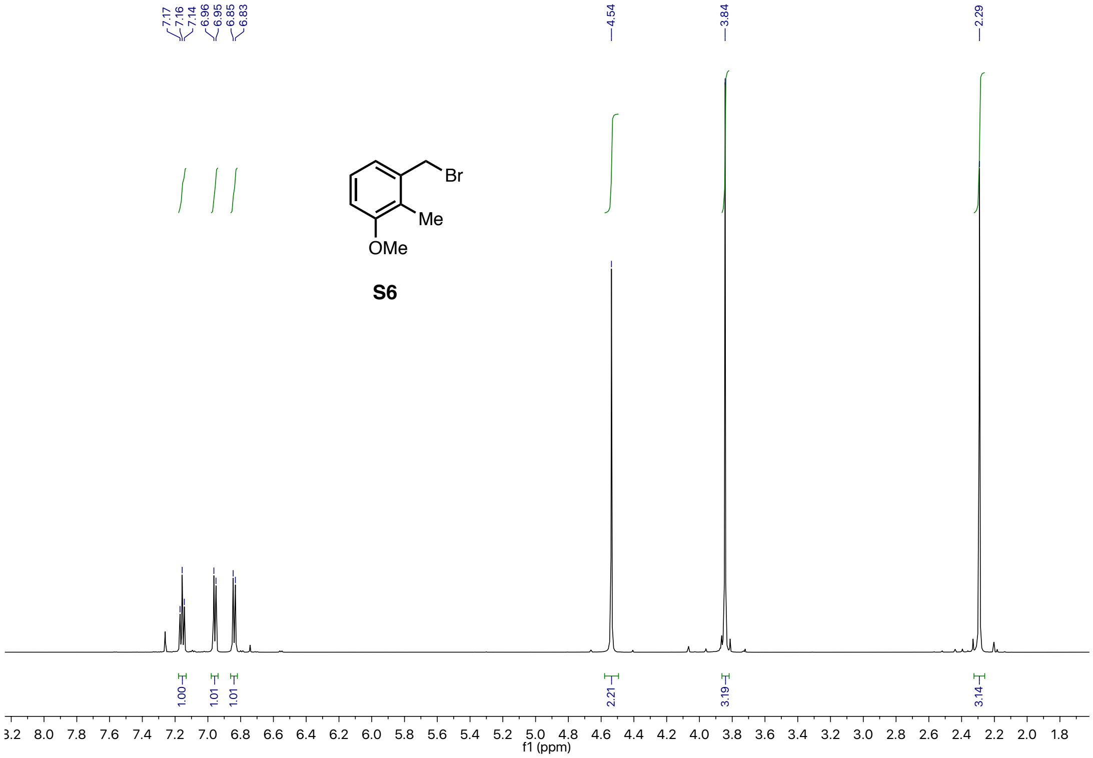

Supplementary Figure 48. ^{1}HNMR of **S6**

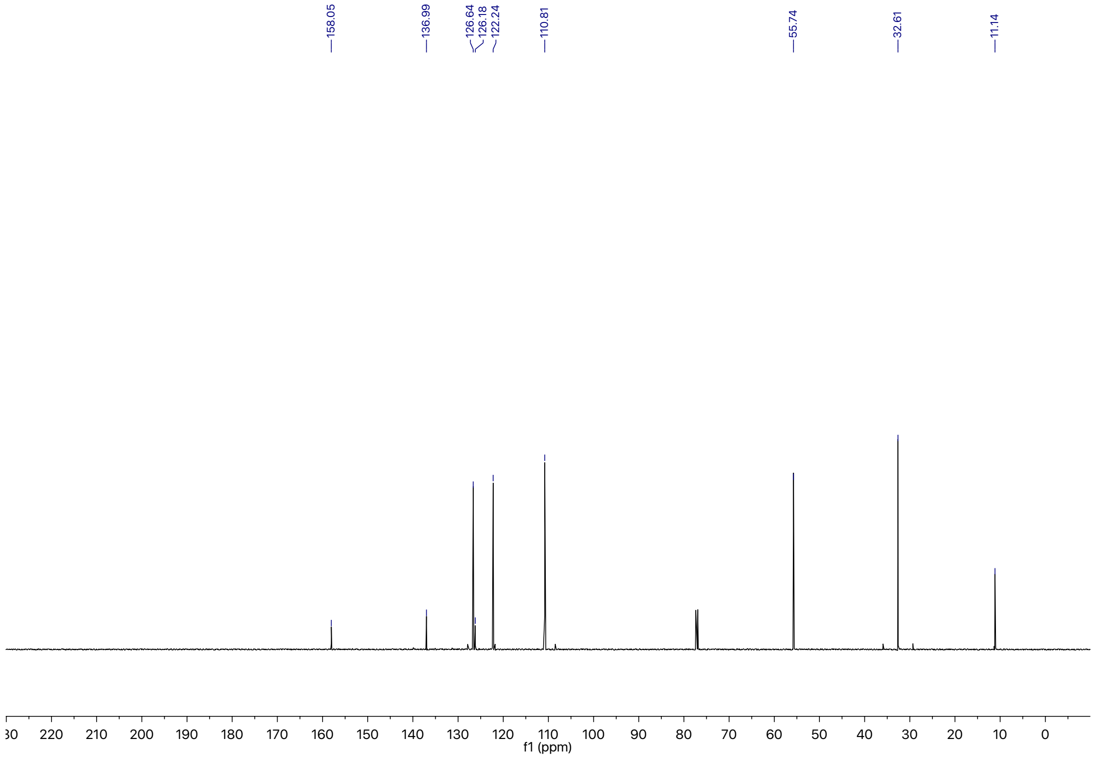

Supplementary Figure 49. ^{13}CNMR of **S6**

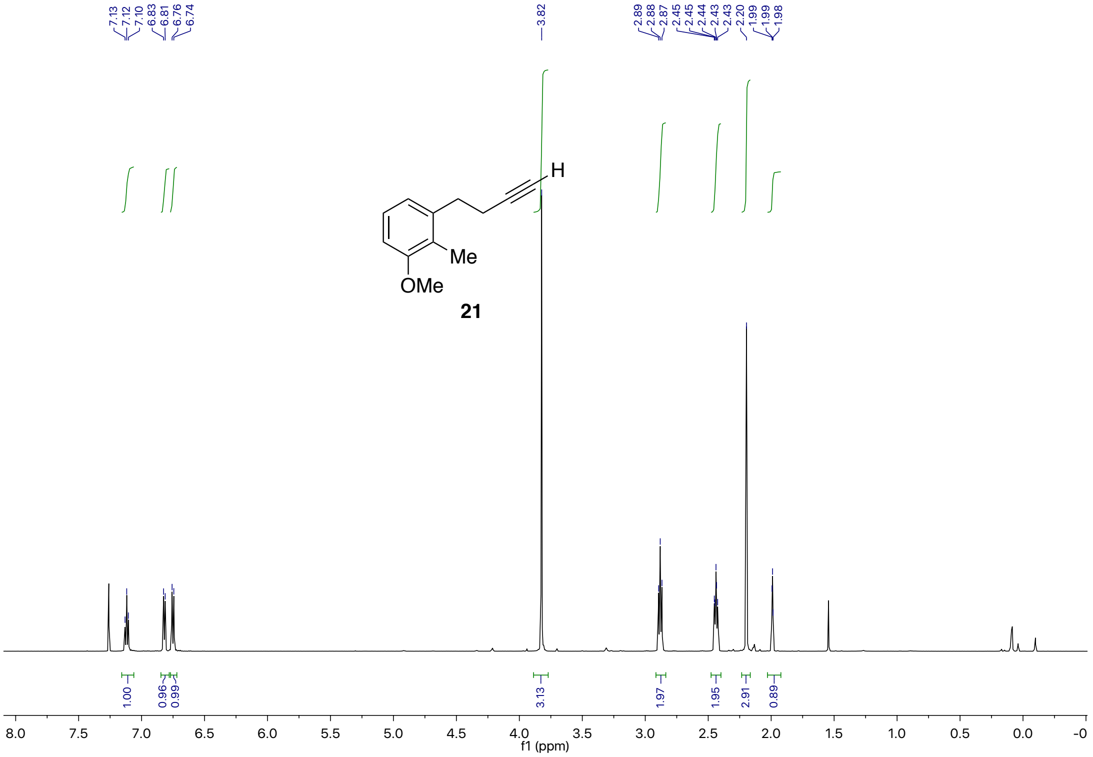

Supplementary Figure 50. ^{1}H NMR of **21**

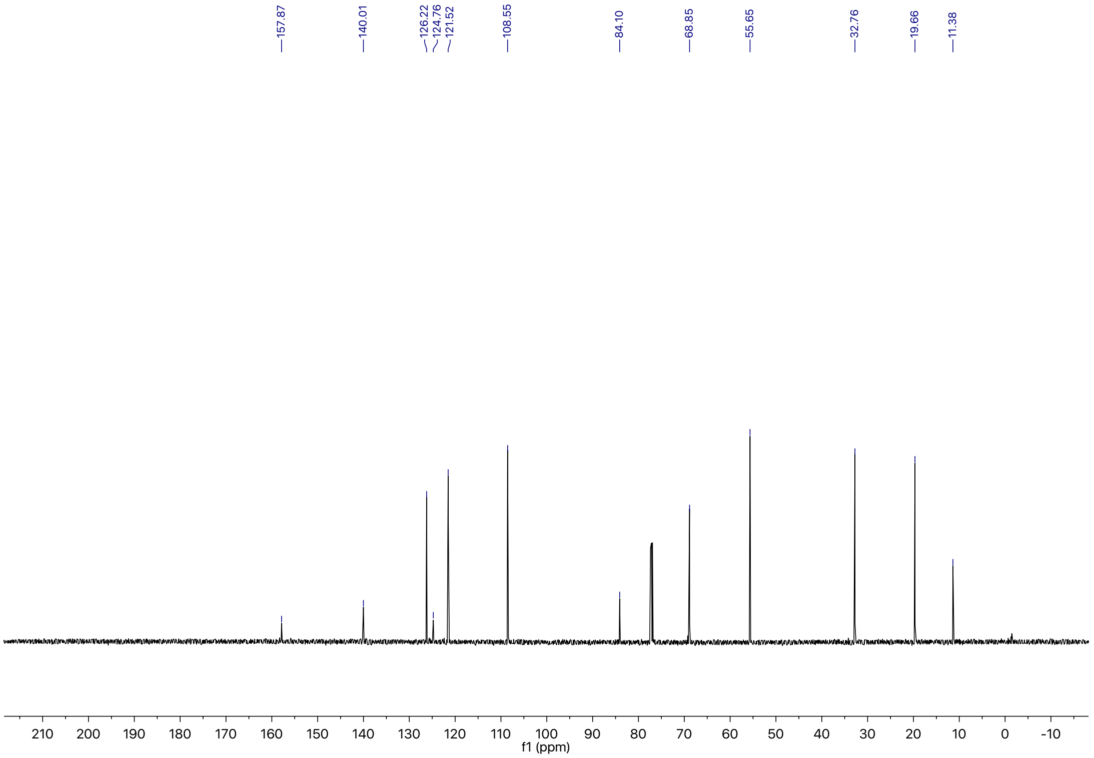

Supplementary Figure 51. ^{13}C NMR of **21**

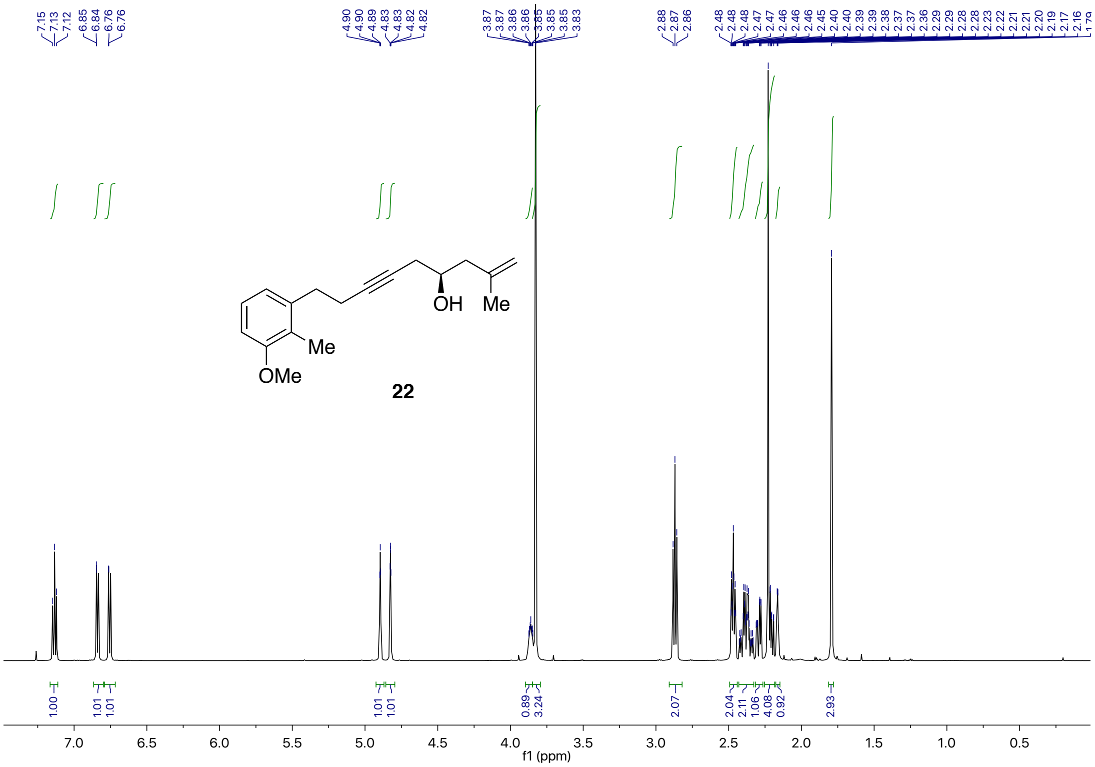

Supplementary Figure 52. ^{1}H NMR of **22**

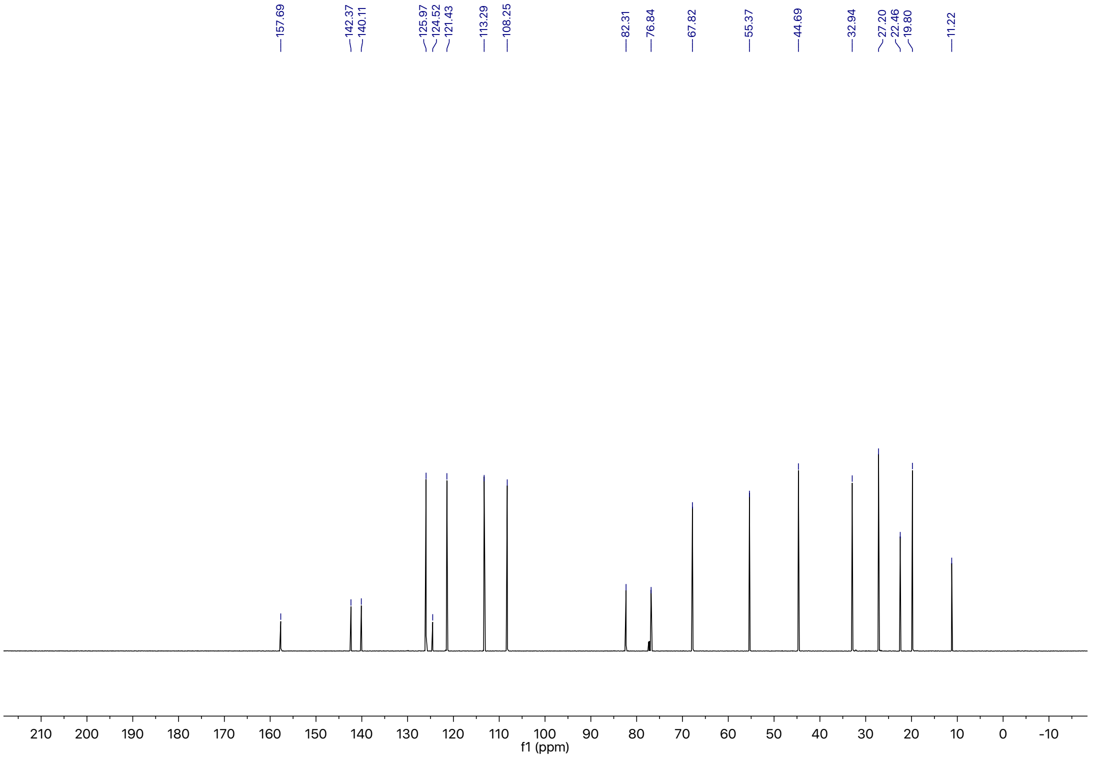

Supplementary Figure 53. ^{13}C NMR of **22**

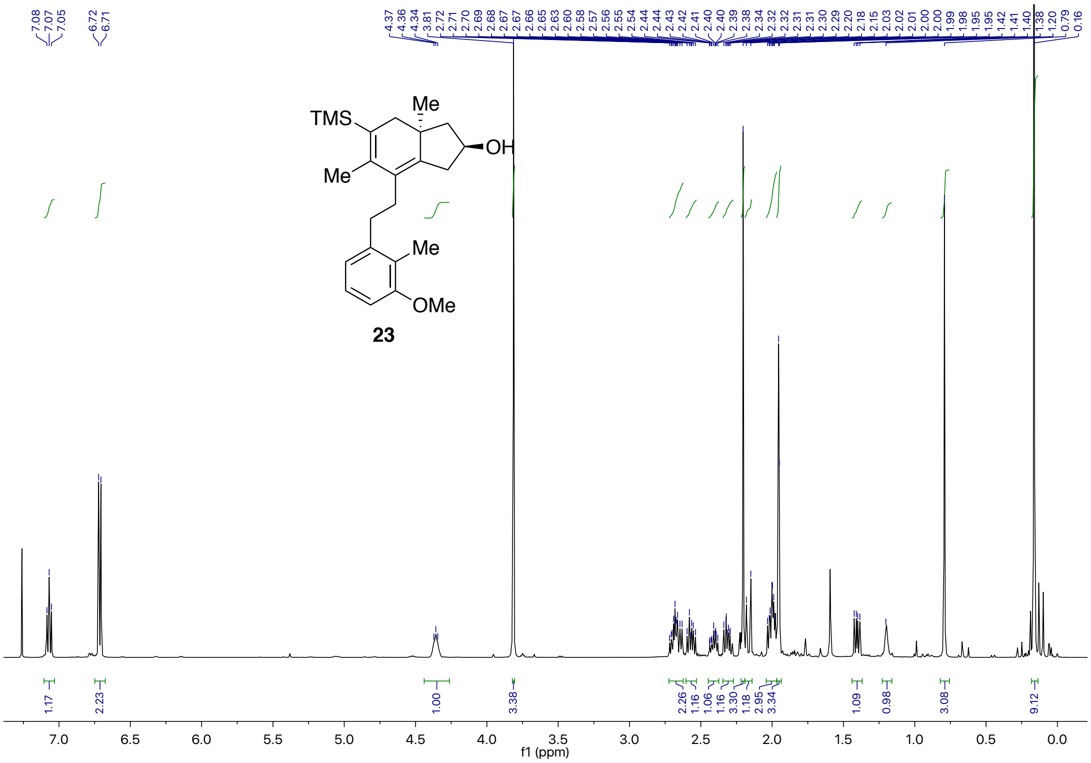

Supplementary Figure 54. ^{1}H NMR of **23**

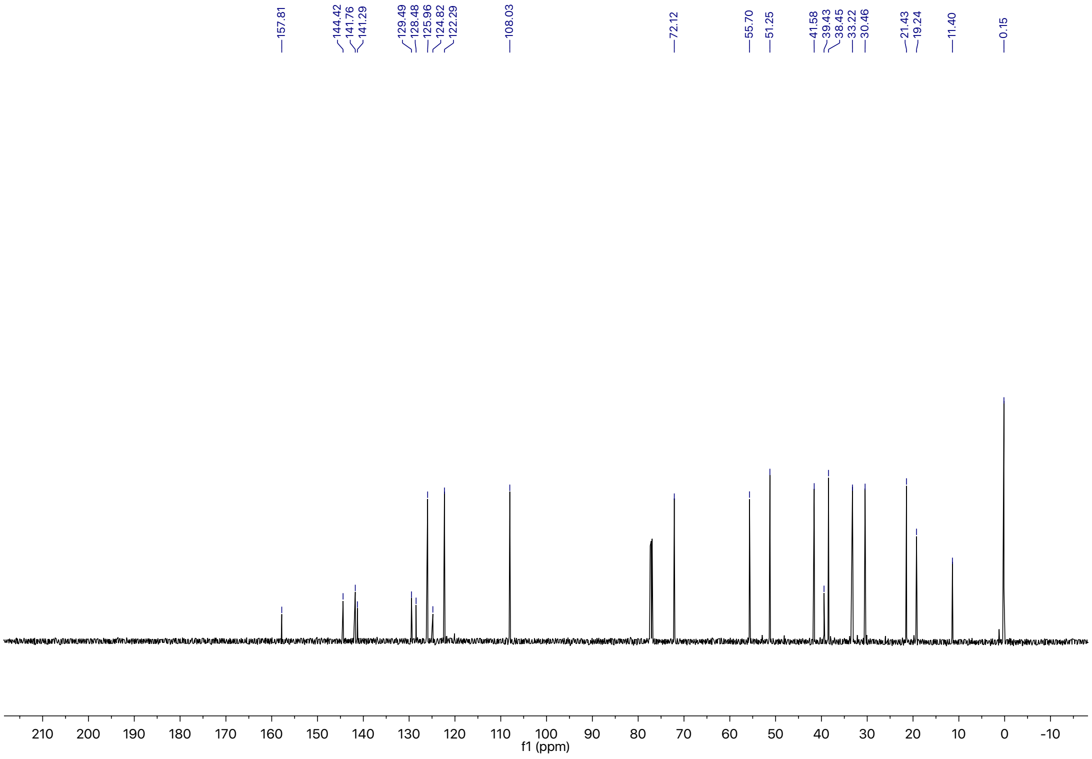

Supplementary Figure 55. ^{13}C NMR of **23**

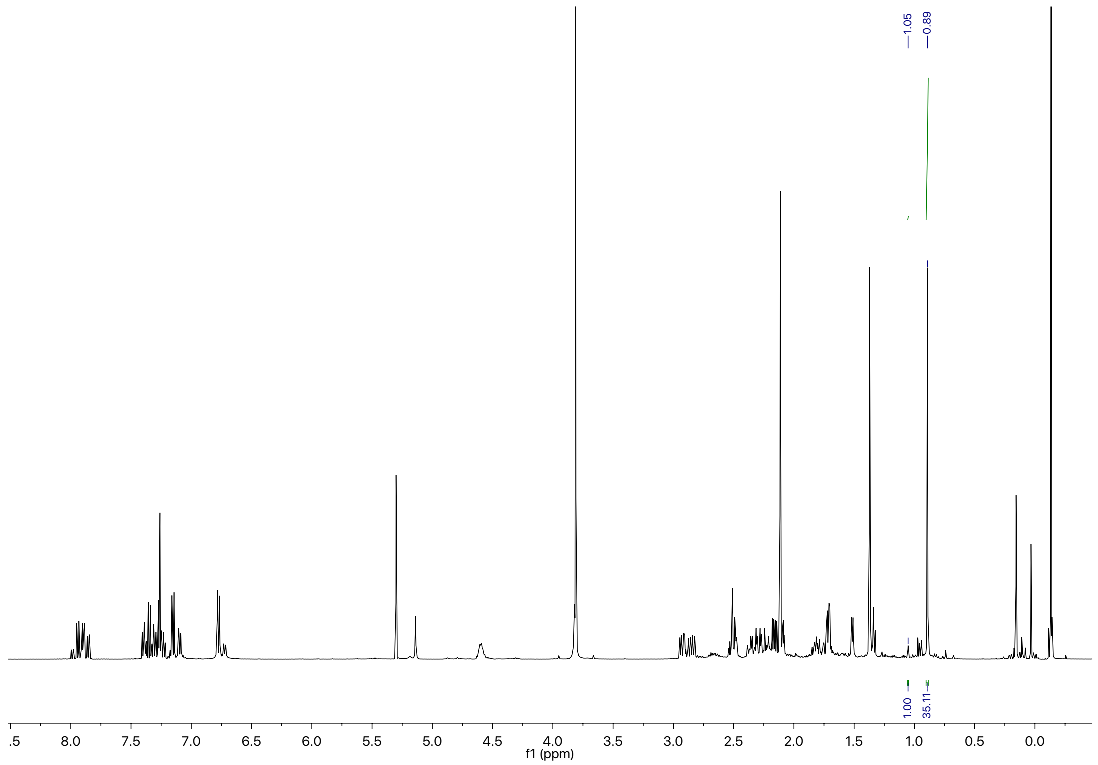

Supplementary Figure 56. Crude ^{1}H NMR of **24**

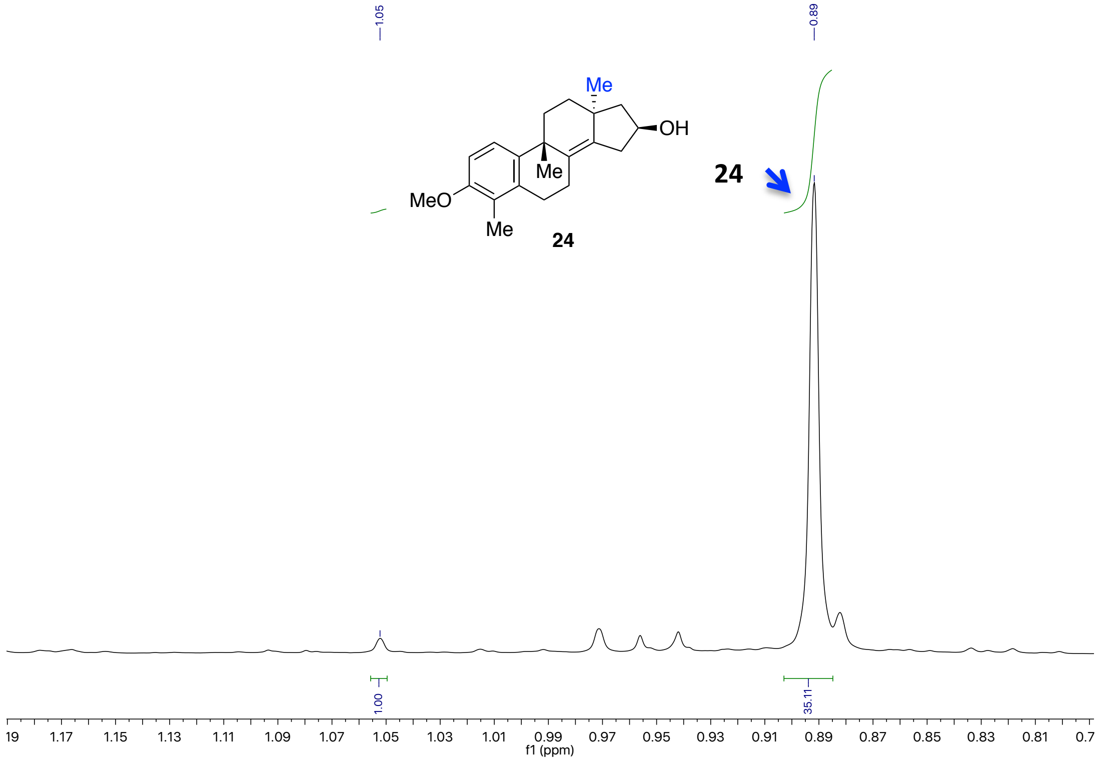

Supplementary Figure 57. Expanded Crude ^{1}H NMR of **24**

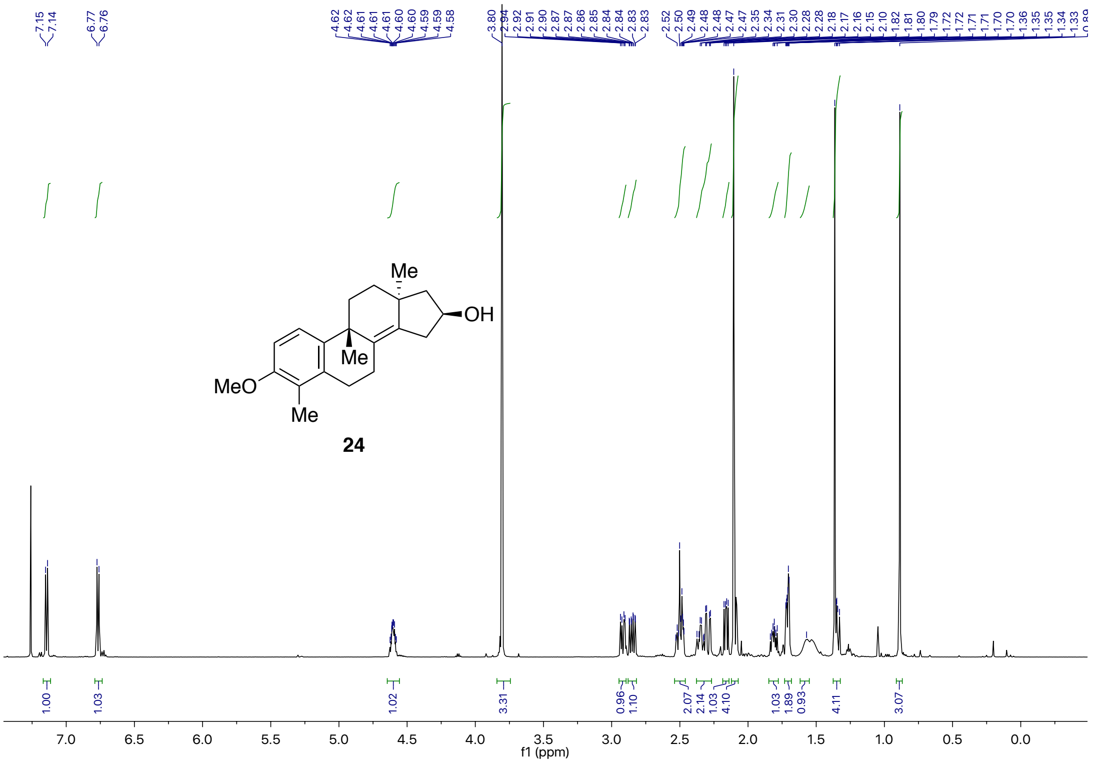

Supplementary Figure 58. ^{1}H NMR of **24**

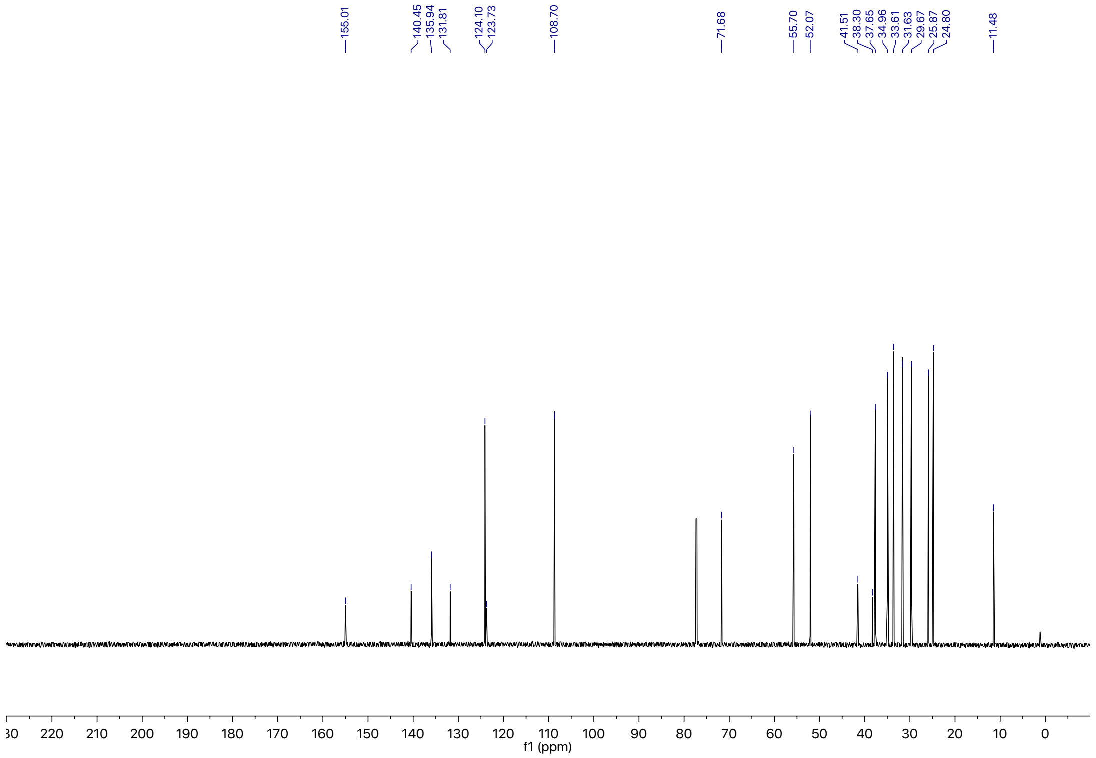

Supplementary Figure 59. ^{13}C NMR of **24**

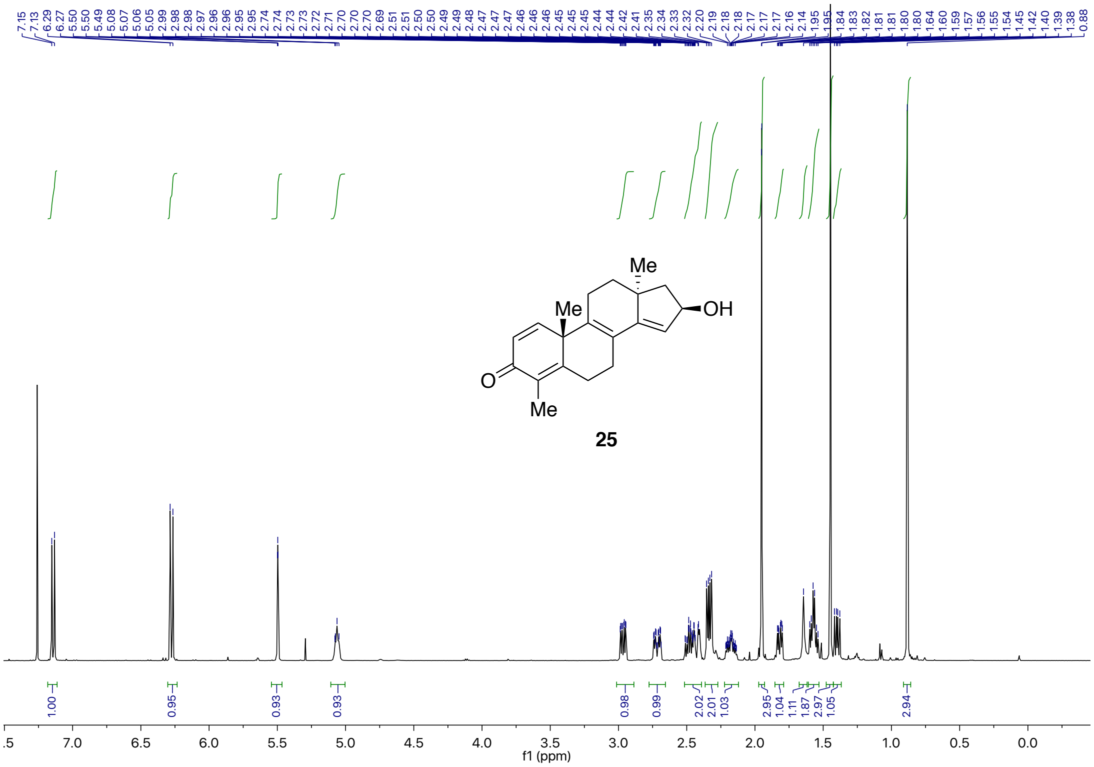

Supplementary Figure 60. ^{1}H NMR of **25**

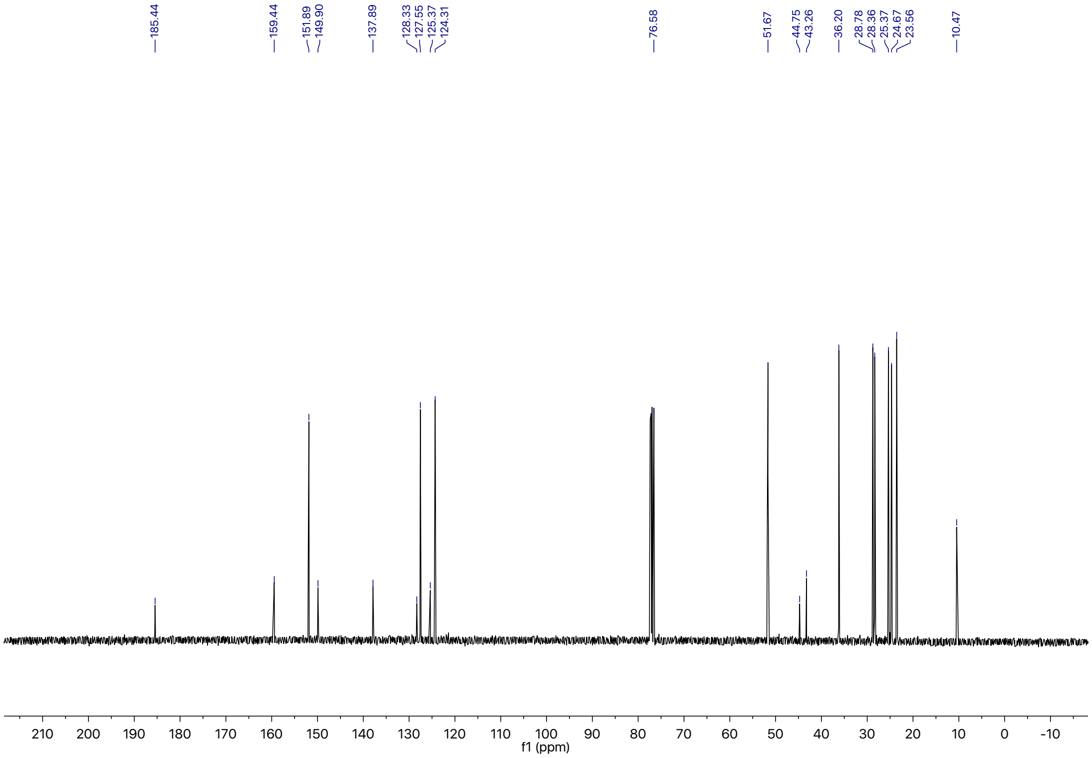

Supplementary Figure 61. ^{13}C NMR of **25**

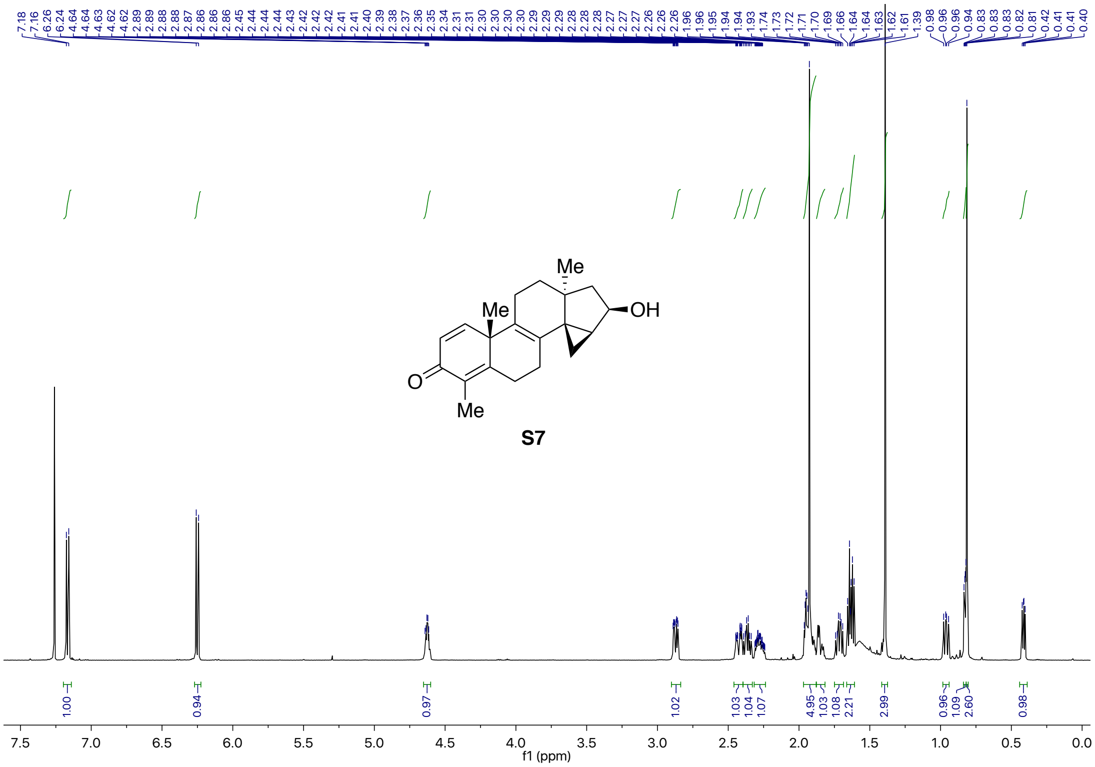

Supplementary Figure 62. ^{1}H NMR of **S7**

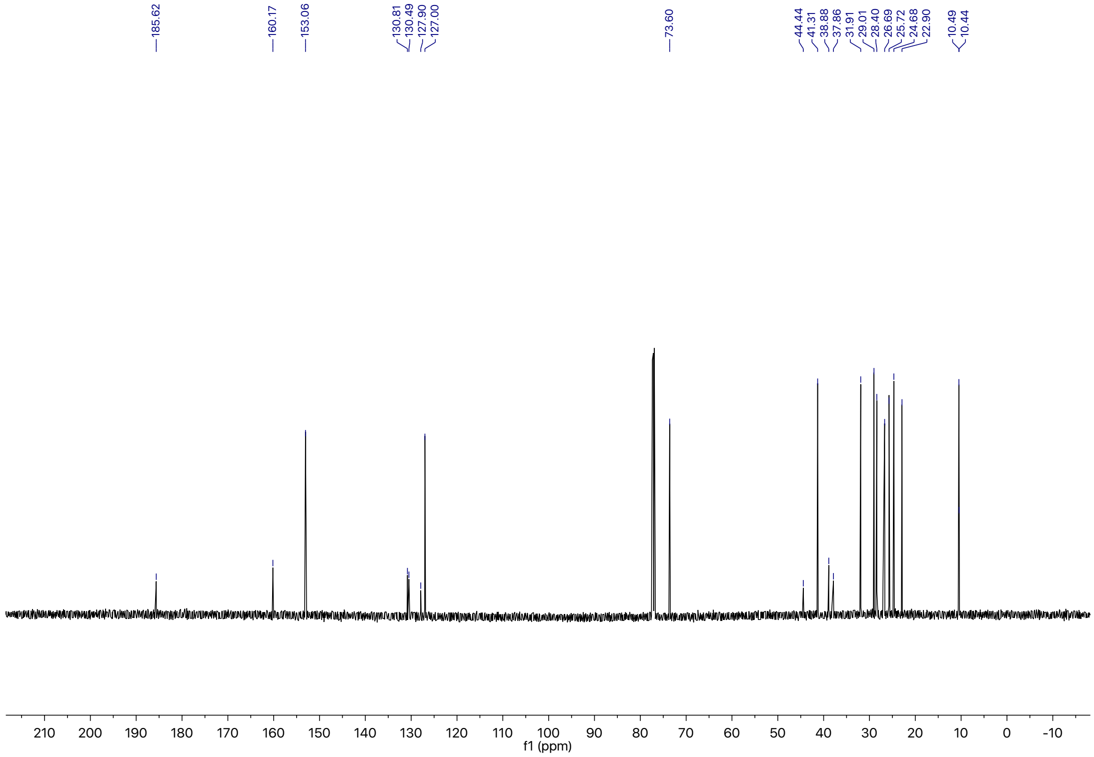

Supplementary Figure 63. ^{13}C NMR of **S7**

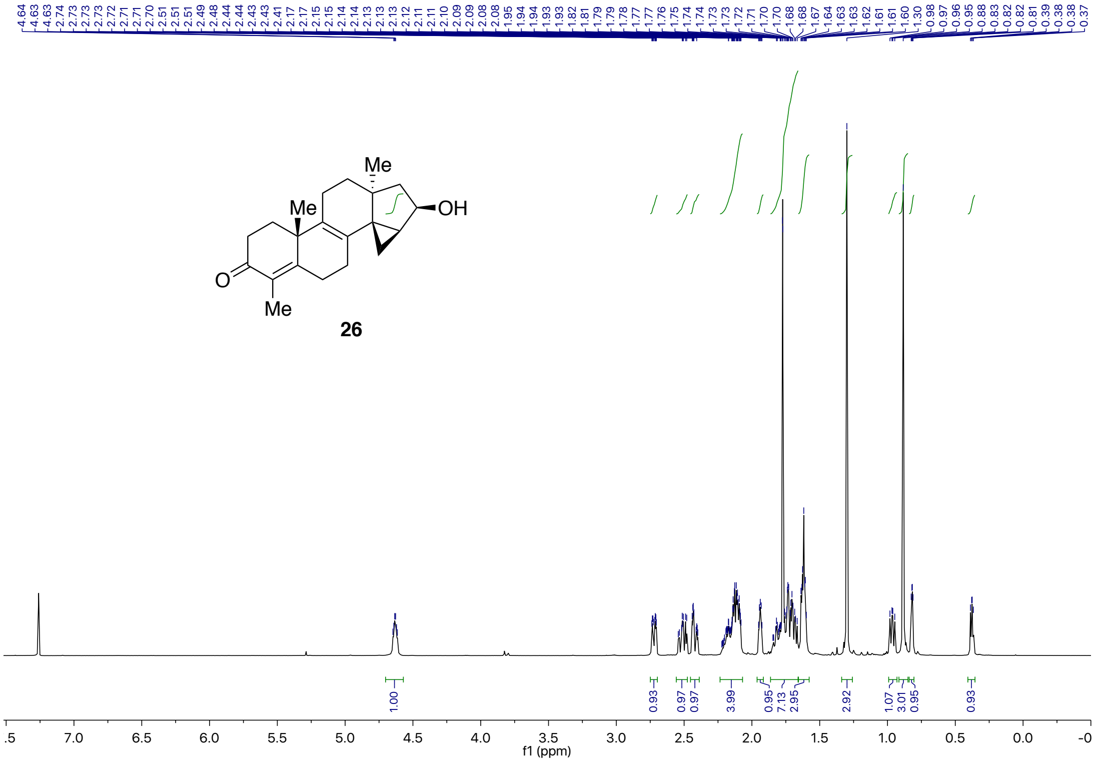

Supplementary Figure 64. ^{1}H NMR of **26**

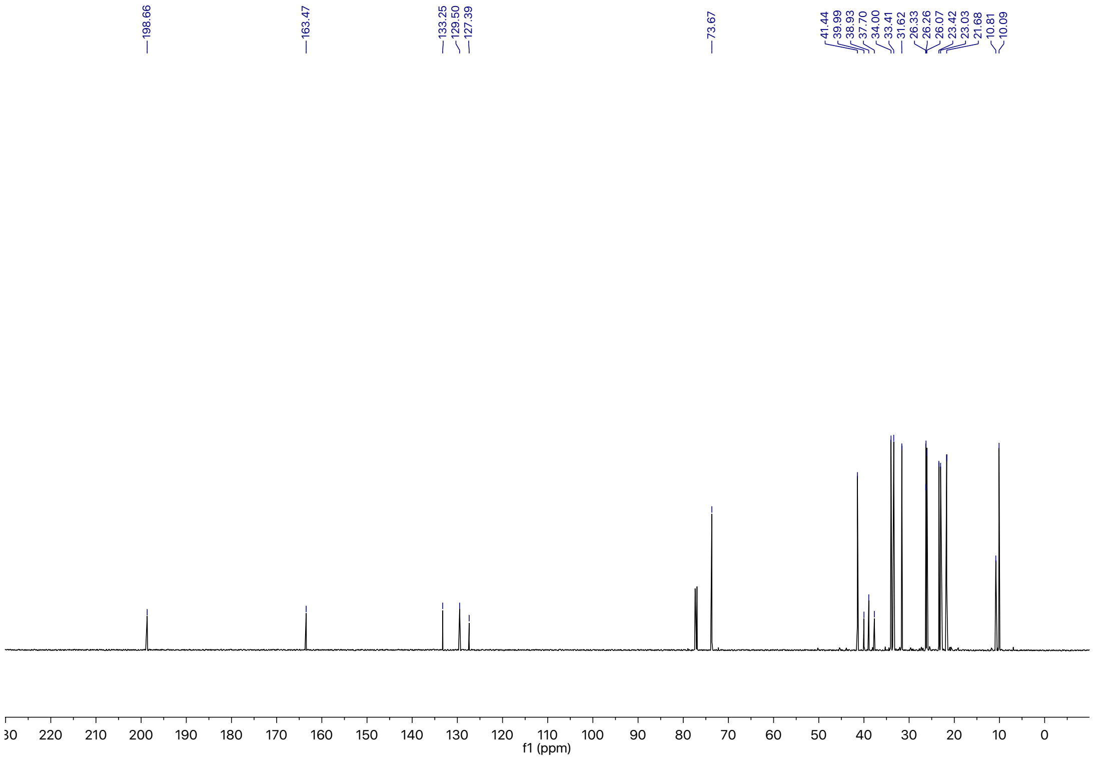

Supplementary Figure 65. ^{13}C NMR of **26**

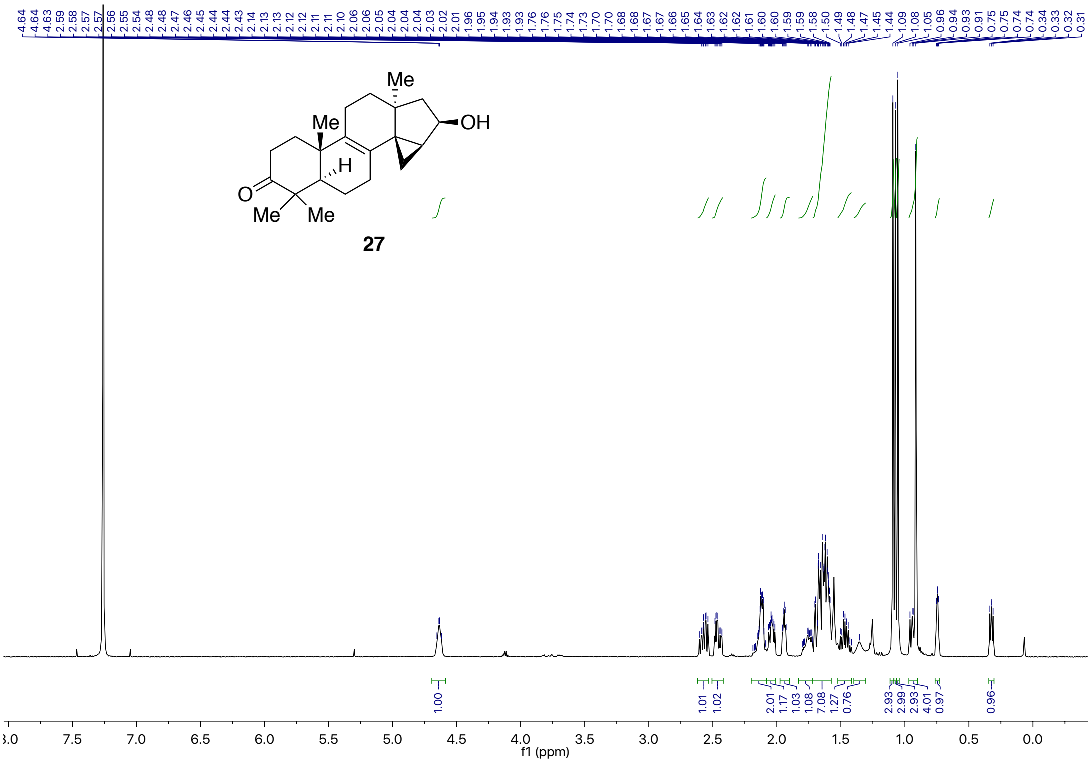

Supplementary Figure 66. ^{1}H NMR of **27**

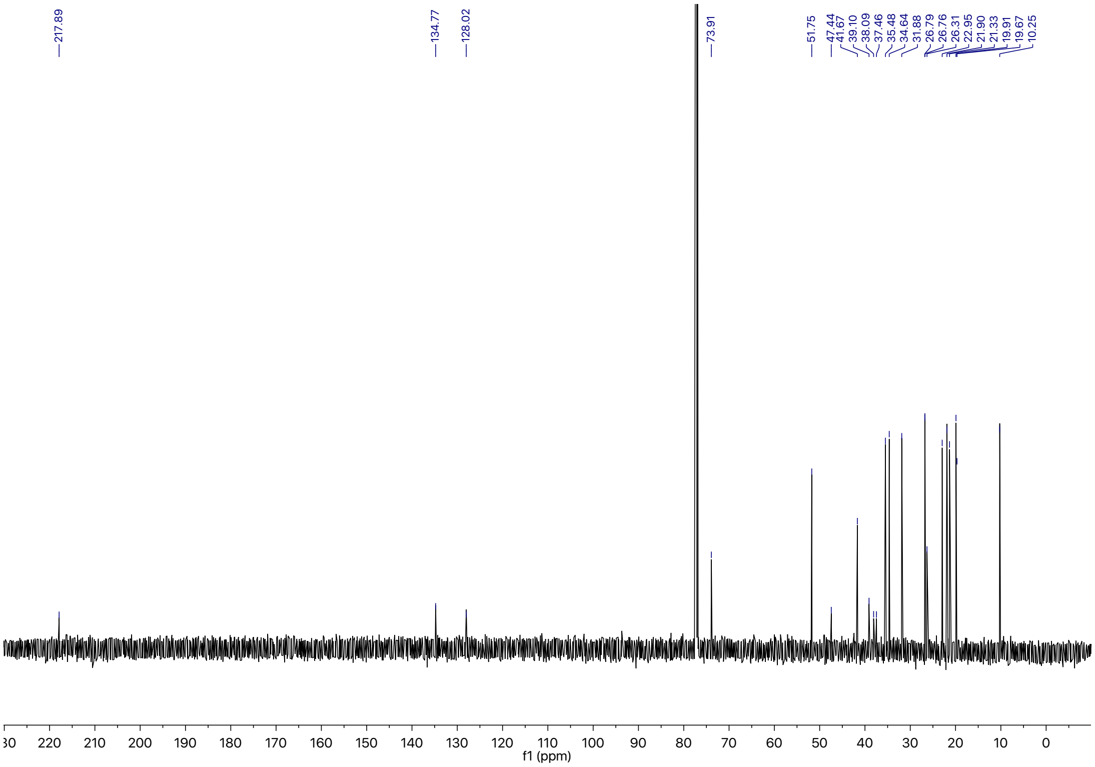

Supplementary Figure 67. ^{13}C NMR of **27**

Supplementary Methods

All reactions were conducted in flame-dried glassware under a nitrogen atmosphere with dry solvents, unless otherwise noted. All reagents and starting materials were purchased from commercial sources and used as supplied, unless otherwise indicated. Anhydrous tetrahydrofuran (THF), toluene (PhMe), and methylene chloride (CH_2Cl_2) were obtained from Glass Contour Solvent Purification System. (*R*)-BINOL and (*S*)-BINOL were purchased from Chem Impex. Titanium isopropoxide (Ti(O*i*-Pr)$_4$) was purchased from Acros, and distilled before use. Solutions of *n*-BuLi (2.5 M in hexanes) were purchased from Aldrich and titrated against N-benzylbenzamide. Yields refer to chromatographically and spectroscopically (^{1}H NMR) homogeneous materials, unless otherwise stated. Flash column chromatography was performed on the Biotage® Automated Liquid Chromatography System Isolera One® using Biotage® SNAP KPM-Sil 10-100 g silica gel cartridges and Biotage® SNAP HP-Sphere ultra 10-25 g silica gel cartridges. Dry column vacuum chromatography was performed on EMD Silica gel 60 (0.015-0.040 mm) as reported.[1] TLC analyses were performed on EMD TLC Silica gel 60 F254 Glass Plates and the spots were visualized by UV-light (254 nm), an aqueous solution of phosphomolybdic acid, ceric sulfate, and sulfuric acid, or a solution of ethanol, surfuric acid, glacial acetic acid, and *p*-anisaldehyde.^{1}H NMR data were recorded on Bruker Avance III 500 and 600 MHz spectrometer (TBI probe) with calibration of spectra to residual $CDCl_3$ (7.26 ppm), CD_3OD (3.31 ppm) and CD_2Cl_2 (5.32 ppm). ^{13}C NMR data were recorded at 125 MHz and 150 MHz on Bruker Avance III 500 and 600 MHz spectrometer (TBI probe) with calibration to the central line of $CDCl_3$ (77.16 ppm), CD_3OD (49.0 ppm) and CD_2Cl_2 (53.84 ppm). Infrared spectra were recorded on a JASCO FT/IRM4100 Fourier Transform Infrared Spectrometer. Optical rotations were measured with a JASCO P-2000 polarimeter, and the concentration (*c*) is reported in g/mL. HRMS (ESI or EI) analyses were performed at the Mass Spectrometry Laboratory of University of Illinois at Urbana-Champaign. All compounds purified by chromatography were sufficiently pure for use in further experiments, unless indicated otherwise. For abbreviations, diisobutylaluminum hydride (DIBAL-H), phenyliodonium diacetate or (diacetoxyiodo) benzene (PIDA), 1,1,1,3,3,3-hexafluoro-2-propanol (HFIP), dimethoxyethane (DME).

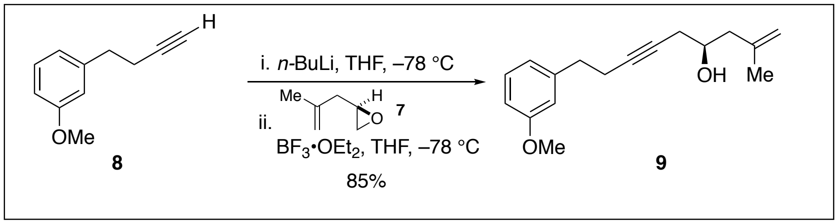

Supplementary Figure 68. Synthesis of Enyne 9

Enyne 9: To a stirring solution of **8**[2] (21.7 g, 135 mmol, 2.49 equiv) in 450 mL THF at –78 °C under N_2 atmosphere was added *n*-BuLi (2.52 M in hexanes, 32.2 ml, 81.1 mmol, 1.50 equiv) dropwise. The resulting mixture was stirred at the same temperature for 45 min, and then BF_3•OEt_2 was added dropwise. The mixture was stirred for 40 min, and then a solution of **7**[3] (6.40 g, 54.1 mmol, 1.0 equiv) in 180 ml THF was added dropwise over 1 hr. The resulting mixture was further stirred for 40 min, and then quenched with 300 mL saturated solution of $NaHCO_3$ at –78 °C. The mixture was warmed to rt, and the organic layer was separated. The aqueous layer was extracted with 3 × 250 mL ethyl acetate. The combined organic layers were dried over $MgSO_4$, filtered, and then the filtrate was concentrated in *vacuo*. SiO_2 flash column chromatography afforded 11.9 g of the title compound **9** as a pale yellow oil (85% isolated yield). Spectral data for **9**: ^{1}H NMR (600 MHz, Chloroform-*d*) δ 7.21 (t, *J* = 7.8 Hz, 1H), 6.81 (d, *J* = 7.9 Hz, 1H), 6.79 – 6.75 (m, 2H), 4.86 (app p, *J* = 1.6 Hz, 1H), 4.81 – 4.76 (m, 1H), 3.85 – 3.80 (m, 1H), 3.79 (s, 3H), 2.80 (t, *J* = 7.5 Hz, 2H), 2.49 (tt, *J* = 7.5, 2.4, 2.4 Hz, 2H), 2.40 – 2.29 (m, 2H), 2.25 (dd, *J* = 14.1, 4.9 Hz, 1H), 2.17 (dd, *J* = 13.8, 8.5 Hz, 1H), 2.01 (d, *J* = 4.0 Hz, 1H), 1.75 (s, 3H); ^{13}C NMR (150 MHz, Chloroform-*d*) δ 159.7, 142.5, 142.4, 129.4, 120.9, 114.4, 113.4, 111.5, 82.3, 77.1, 67.9, 55.2, 44.8, 35.4, 27.2, 22.5, 20.9; IR (thin film): 3452, 2933, 2835, 1602, 1585, 1491, 1452, 1153 cm^{-1}; HRMS (ESI-TOF): calculated for $C_{17}H_{23}O_2$ $[M+H]^+$ 259.1698, found 259.1702; $[\alpha]_D^{23}$ = –1.7 (*c* 0.084, $CHCl_3$). See Supplementary Figures 8 and 9 for ^{1}HNMR and ^{13}CNMR spectra.

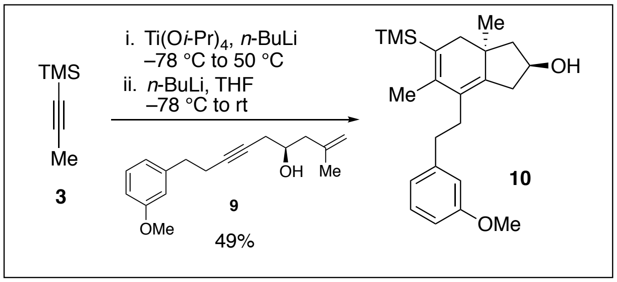

Supplementary Figure 69. Synthesis of Hydrindane **10**

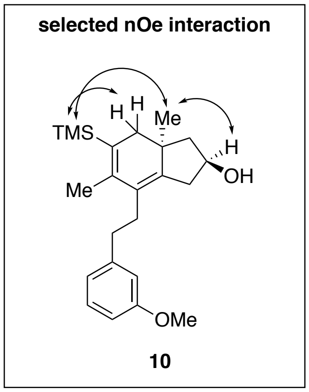

Supplementary Figure 70. Selected nOe interaction

Hydrindane 10: To a stirring solution of **3** (17 g, 0.15 mol, 3.3 equiv) and Ti(O*i*-Pr)$_4$ (45 g, 0.15 mol, 3.3 equiv) in 500 mL anhydrous toluene at –78 °C under N_2 atmosphere was added *n*-BuLi (2.5 M in hexanes, 119 mL, 0.30 mol, 6.5 equiv) dropwise. After the addition, the cooling bath was removed, and the resulting dark brown mixture was warmed to rt, and then further warmed to 50 °C. The reaction mixture was stirred for 1 hr at the same temperature without a reflux condenser, and then cooled to rt. A separate round bottom flask charged with a solution of **9** (12 g, 46 mmol, 1.0 equiv) in 200 mL anhydrous toluene at –78 °C under N_2 atmosphere was added *n*-BuLi (2.5 M in hexanes, 18 mL, 46 mmol, 1.0 equiv) dropwise. The resulting solution was warmed to rt, cannulated into the above dark brown Ti–Alkyne mixture, and then stirred overnight at rt under N_2 atmosphere (approx. 12 hr). After this period, 250 mL saturated solution of $NaHCO_3$ was added to the reaction mixture. The organic layer was separated, and the aqueous layer was extracted with 500 mL × 8 ethyl acetate. The combined organic layers were dried with $MgSO_4$, filtered, and the filtrate was concentrated in *vacuo*. Purification of the crude product by SiO_2 flash column chromatography using 5% ethyl acetate/95% hexanes, 10% ethyl acetate/90% hexanes, 15% ethyl acetate/85% hexanes as the eluent afforded 3.9 g of the title compound **10** as a thick yellow oil (70% isolated yield). Spectral Data for **10**: ^{1}H NMR (500 MHz, Chloroform-*d*) δ 7.19 (t, *J* = 7.8 Hz, 1H), 6.77 – 6.72 (m, 2H), 6.68 – 6.70 (m, 1H), 4.36 (p, *J* = 6.8 Hz, 1H), 3.79 (s, 3H), 2.69 – 2.60 (m, 2H), 2.57 – 2.43 (m, 2H), 2.38 – 2.30 (m, 1H), 2.17 (d, *J* = 15.8 Hz, 1H), 2.04 – 1.95 (m, 3H), 1.93 (app d, *J* = 2.6 Hz, 3H), 1.39 (dd, *J* = 12.3, 7.7 Hz, 1H), 1.28 (s, 1H), 0.79 (s, 3H), 0.16 (s, 9H); ^{13}C NMR (150 MHz, Chloroform-*d*) δ 159.6, 144.5, 143.9, 141.2, 129.28, 129.26, 128.5, 121.3, 114.8, 111.1, 72.1, 55.3, 51.3, 41.6, 39.5, 38.5, 35.7, 31.6, 21.3, 19.2, 0.2; IR (thin film): 3348, 2949, 2857, 1602, 1584, 1454, 1248, 1058, 835 cm^{-1}; HRMS (ESI–TOF): calculated for $C_{23}H_{35}O_2Si$ $[M+H]^+$ 371.2406, found 371.2393; $[\alpha]_D^{22}$ = –44.0 (*c* 0.022, $CHCl_3$). See Supplementary Figures 10 and 11 for ^{1}HNMR and ^{13}CNMR spectra.

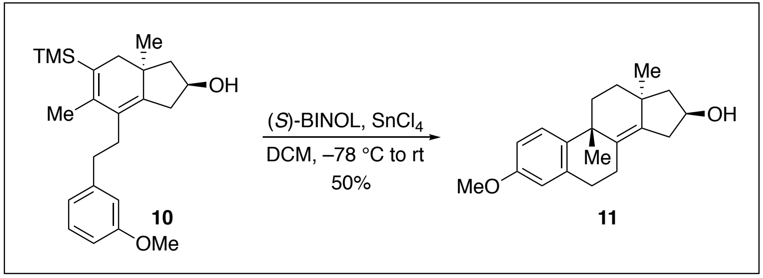

Supplementary Figure 71. Synthesis of Tetracycle **11**

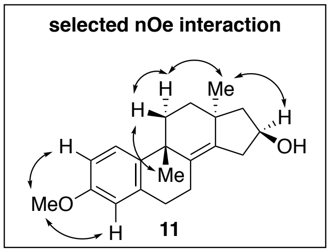

Supplementary Figure 72. Selected nOe interaction

Tetracyle 11: To a stirring suspension (see supplementary note **1**) of (*S*)–BINOL (13 g, 44 mmol, 5.3 equiv) in 450 mL dichloromethane at –78 °C under N_2 atmosphere was added a solution of $SnCl_4$ (1.0 M in dichloromethane, 44 mL, 44 mmol, 5.4 equiv) dropwise using syringe. The resulting mixture was stirred for 18 min at –78 °C, and then a solution of **10** (3.1 g, 8.2 mmol, 1.0 equiv) in 150 mL dichloromethane was added dropwise over 1 hr 20 min *via* cannula. The resulting mixture was stirred for an additional 1 hr at –78 °C, the cooling bath was removed, and then warmed to rt over 50 min. At this point, the reaction was judged to be complete by TLC-analysis. A 100 mL saturated solution of $NaHCO_3$ was added, and the resulting mixture was further diluted with 200 mL dichloromethane. The organic layer was separated, and the aqueous layer was extracted with 500 mL × 2 ethyl acetate. The combined organic layers were dried over Na_2SO_4, filtered, and the filtrate was concentrated in *vacuo*. The crude product was purified by dry column vacuum chromatography[1] using 7 cm × 6.5 cm (height × diameter) SiO_2 column, and 1% ethyl acetate–10% ethyl acetate (1% gradient/fraction) in dichloromethane as the eluent (200 mL/fraction) to afford 1.2 g of the title compound **11** as a thick yellow oil (50% isolated yield). Spectral Data for **11** ^{1}H NMR (500 MHz, Chloroform-*d*) δ 7.21 (d, *J* = 8.7 Hz, 1H), 6.76 (dd, *J* = 8.7, 2.6 Hz, 1H), 6.58 (d, *J* = 2.8 Hz, 1H), 4.65 – 4.56 (m, 1H), 3.78 (s, 3H), 2.90 – 2.81 (m, 2H), 2.72 (ddd, *J* = 16.3, 11.7, 5.7 Hz, 1H), 2.46 – 2.32 (m, 2H), 2.27 (dd, *J* = 16.8, 4.2 Hz, 1H), 2.17 (dd, *J* = 12.0, 6.7 Hz, 1H), 2.09 (dt, *J* = 13.1, 3.3 Hz, 1H), 1.88 – 1.80 (m, 1H), 1.76 – 1.68 (m, 2H), 1.58 (s, 1H), 1.38 – 1.30 (m, 4H), 0.90 (s, 3H); ^{13}C NMR (150 MHz, Chloroform-*d*) δ 157.1, 140.1, 137.3, 136.4, 132.0, 127.3, 113.1, 112.5, 71.6, 55.3, 52.0, 41.6, 38.1, 37.7, 34.4, 33.4, 32.3, 31.4, 25.9, 25.0; IR (thin film): 3375, 2933, 2853, 1608, 1498, 1273, 1233, 1035, 732 cm^{-1}; HRMS (ESI-TOF): calculated for $C_{20}H_{27}O_2$ $[M+H]^+$ 299.2011, found 299.2012; $[\alpha]_D^{22}$ = –223.0 (*c* 0.014 , $CHCl_3$). See Supplementary Figures 16 and 17 for ^{1}HNMR and ^{13}CNMR spectra.

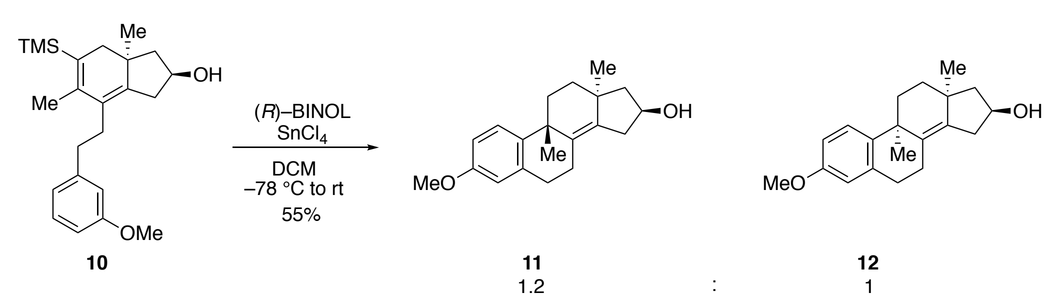

Supplementary Figure 73. Synthesis of Tetracycle **11** and **12**

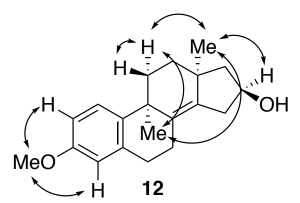

Supplementary Figure 74. Selected nOe interaction

Tetracycle 11 and 12: To a stirring suspension (see supplementary note **1**) of (*R*)–BINOL (1.0 g, 3.6 mmol, 5.4 equiv) in 36 mL dichloromethane at –78 °C under N_2 atmosphere was added a solution of $SnCl_4$ (1.0 M in dichloromethane, 3.6 mL, 3.6 mmol, 5.4 equiv) dropwise using syringe. The resulting mixture was stirred for 21 min at –78 °C, and then a solution of **10** (0.25 g, 0.68 mmol, 1.0 equiv) in 13 mL dichloromethane was added dropwise over 3 min *via* syringe. The resulting mixture was stirred for 1.8 hr at –78 °C, and then warmed to rt over 8 min. At this point, the reaction was judged to be complete by TLC-analysis. 50 mL saturated solution of $NaHCO_3$ was added, stirred for 20 min, and then further diluted with 100 mL dichloromethane. The organic layer was separated, and the aqueous layer was extracted with 100 mL × 2 dichloromethane. The combined organic layers were dried over Na_2SO_4, filtered, and the filtrate was concentrated in *vacuo*. The crude product was obtained as a 1.2:1 mixture of **11** and **12**. A subsequent purification by SiO_2 flash column chromatography afforded 40 mg of the title compound **12** as a thick yellow film (20% isolated yield), and 71 mg of 4:1 mixture of **11** and **12** as a thick yellow film (35% isolated yield, 55% combined yield of **11** and **12**). Spectral data for **12**: ^{1}H NMR (500 MHz, Methylene Chloride-d_2) δ 7.24 (d, *J* = 8.7 Hz, 1H), 6.70 (dd, *J* = 8.7, 2.9 Hz, 1H), 6.56 (d, *J* = 2.9 Hz, 1H), 4.52 (tdt, *J* = 8.2, 6.4, 5.3 Hz, 1H), 3.74 (s, 3H), 2.86 – 2.78 (m, 3H), 2.50 – 2.42 (m, 1H), 2.41 – 2.32 (m, 1H), 2.29 – 2.18 (m, 2H), 1.99 (dd, *J* = 12.0, 6.5 Hz, 1H), 1.91 (ddd, *J* = 14.2, 12.3, 3.5 Hz, 1H), 1.57 (ddd, *J* = 12.8, 5.6, 3.5 Hz, 1H), 1.44 (d, *J* = 5.3 Hz, 1H), 1.31 (s, 3H), 1.25 – 1.17 (m, 2H), 1.04 (s, 3H); ^{13}C NMR (150 MHz, Chloroform-*d*) δ 157.3, 139.0, 138.6, 137.1, 133.6, 126.4, 113.6, 112.1, 71.1, 55.3, 51.5, 41.3, 39.1, 38.1, 34.4, 34.2, 33.0, 31.9, 25.9, 24.9; IR (thin film): 3335, 2950, 2919, 2861, 1607, 1497, 1451, 1272, 1231, 1039 cm^{-1}; HRMS (EI-TOF): calculated for $C_{20}H_{26}O_2$ [M$^+$] 298.1933, found 298.1938; $[\alpha]_D^{23}$ = +139.7 (*c* 0.0065, $CHCl_3$). See Supplementary Figures 18 and 19 for ^{1}HNMR and ^{13}CNMR spectra.

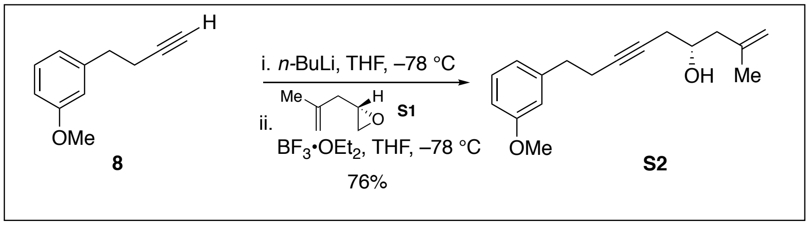

Supplementary Figure 75. Synthesis of Enyne **S2**

Enyne S2: To a stirring solution of **8**[2] (23.2 g, 145 mmol, 2.50 equiv) in 400 mL THF at –78 °C under N_2 atmosphere was added *n*-BuLi (2.33 M in hexanes. 37.3 ml, 87.0 mmol, 1.50 equiv) dropwise. The resulting mixture was stirred at the same temperature for 44 min, and then BF_3•OEt_2 was added dropwise. The mixture was stirred for 33 min, and then a solution of **S1**[5] (7.72 g, 58.0 mmol, 1.00 equiv)[3] in 60 ml THF was added dropwise over 20 min. The mixture was stirred for 45 min, and the reaction was quenched with 200 mL saturated solution of $NaHCO_3$ at –78 °C. The mixture was warmed to rt and the organic layer was separated. The aqueous layer was extracted with 200 mL x 3 ethyl acetate. The combined organic layers were dried over Mg_2SO_4, filtered, and the filtrate was concentrated in *vacuo*. SiO_2 flash column chromatography afforded 11.5 g of the title compound **S2** as a yellow oil (76% isolated yield). Spectral data for **S2**: ^{1}H NMR (500 MHz, Chloroform-*d*) δ 7.22 (t, *J* = 7.8 Hz, 1H), 6.82 (d, *J* = 7.6, 1.2 Hz, 1H), 6.80 – 6.73 (m, 2H), 4.86 (m, 1H), 4.79 (dd, *J* = 2.2, 1.1 Hz, 1H), 3.86 – 3.80 (m, 1H), 3.79 (s, 3H), 2.80 (t, *J* = 7.5 Hz, 2H), 2.49 (tt, *J* = 7.5, 2.4 Hz, 2H), 2.41 – 2.29 (m, 2H), 2.25 (ddd, *J* = 13.9, 4.9, 1.2 Hz, 1H), 2.17 (dd, *J* = 13.9, 8.2 Hz, 1H), 2.08 (d, *J* = 4.3 Hz, 1H), 1.76 (s, 1H).[1] ^{13}C NMR (150 MHz, Chloroform-*d*) δ 159.6, 142.39, 142.35, 129.3, 120.8, 114.3, 113.3, 111.5, 82.2, 77.1, 67.8, 55.1, 44.7, 35.3, 27.2, 22.5, 20.8; IR (thin film): 3077, 2936, 2833, 1600, 1583, 1488, 1452, 1275, 1149, 1051, 891, 694 cm^{-1}; HRMS (ESI-TOF): calculated for $C_{17}H_{23}O_2$ $[M+H]^+$ 259.1698, found 259.1699; $[\alpha]_D^{22}$ = +1.5 (0.0252, $CHCl_3$). See Supplementary Figures 20 and 21 for ^{1}HNMR and ^{13}CNMR spectra.

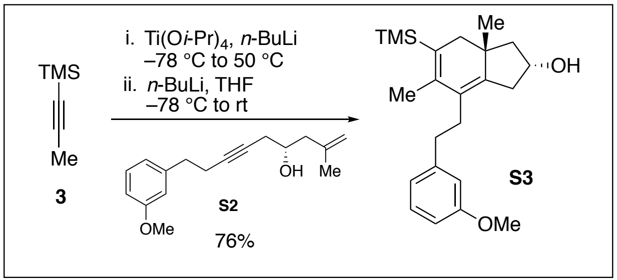

Supplementary Figure 76. Synthesis of Hydrindane **S3**

Hydrindane S3: To a stirring solution of 1-(Trimethylsilyl)propyne (**3**) (66.4 mL, 448 mmol, 3.30 equiv) and Ti(O*i*-Pr)$_4$ (132 mL, 448 mmol, 3.30 equiv) in 2.24 L anhydrous toluene at –78 °C under N_2 atmosphere was added *n*-BuLi (2.52 M in hexanes, 350 mL, 883 mmol, 6.50 equiv) dropwise. After the addition, the cooling bath was removed, and the resulting dark brown mixture was warmed to rt, and then further warmed to 55 °C. The reaction mixture was stirred for 1 hr at the same temperature without a reflux condenser, and then cooled to rt. A separate round bottom flask charged with a solution of **S2** (35.1 g, 136 mmol, 1.00 equiv) in 250 mL anhydrous toluene at –78 °C was added *n*-BuLi (2.52 M in hexanes, 53.9 mL, 136 mmol, 1.00 equiv) dropwise. The resulting solution was warmed to rt, cannulated into the above dark brown mixture, and then stirred overnight at rt under N_2 atmosphere (approx. 12 hr). After this period, 800 mL saturated solution of $NaHCO_3$ was added to the reaction mixture. The organic layer was separated, and the aqueous layer was extracted with 500 mL × 6 ethyl acetate. The combined organic layers were dried with $MgSO_4$, filtered, and the filtrate was concentrated in vacuo. The crude product was purified by flash chromatography to afford 38.5 g of **S3** as a yellow oil (76 % isolated yield). Spectral Data for **S3**: ^{1}H NMR (600 MHz, Chloroform-*d*) δ 7.18 (t, *J* = 7.9 Hz, 1H), 6.77 – 6.72 (m, 2H), 6.69 (t, *J* = 2.0 Hz, 1H), 4.36 (app p, *J* = 7.1 Hz, 1H), 3.79 (s, 3H), 2.70 – 2.60 (m, 2H), 2.56 – 2.50 (m, 1H), 2.48 – 2.42 (m, 1H), 2.37 – 2.31 (m, 1H), 2.17 (d, *J* = 15.9 Hz, 1H), 2.03 – 1.95 (m, 3H), 1.93 (app d, *J* = 2.6 Hz, 3H), 1.39 (dd, *J* = 12.3, 7.7 Hz, 1H), 1.26 (s, 1H), 0.79 (s, 3H), 0.16 (s, 9H). ^{13}C NMR (150 MHz, Chloroform-*d*) δ 159.6, 144.5, 143.9, 141.2, 129.29, 129.27, 128.5, 121.3, 114.9, 111.1, 72.1, 55.3, 51.3, 41.6, 39.5, 38.5, 35.7, 31.6, 21.4, 19.2, 0.2; IR (thin film): 3354, 2964, 2852, 1600, 1580, 1453, 1246, 1052, 835, 693 cm^{-1}; HRMS (ESI–TOF): calculated for $C_{23}H_{35}O_2Si$ [M+H]$^+$ 371.2406, found 371.2406; $[\alpha]_D^{22}$ = +30.2 (*c* 0.0133, $CHCl_2$). See Supplementary Figures 22 and 23 for ^{1}HNMR and ^{13}CNMR spectra.

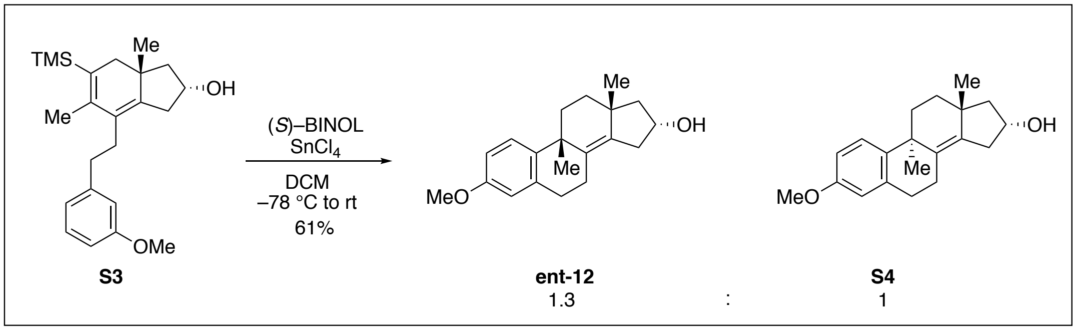

Supplementary Figure 77. Synthesis of Tetracycle **ent-12**

Tetracycle ent-12: To a stirring suspension (see supplementary note **1**) of (*S*)–BINOL (16 g, 56mmol, 1.2 equiv) in 280 mL dichloromethane at –78 °C under N_2 atmosphere was added a solution of $SnCl_4$ (1.0 M in dichloromethane, 56 mL, 56 mmol, 1.2 equiv) dropwise using syringe. The resulting mixture was stirred for 25 min at –78 °C, and then a solution of **S3** (17 g, 46 mmol, 1.0 equiv) in 230 mL dichloromethane was added dropwise over 1 hr *via* canula. The resulting mixture was stirred for 2 hr at –78 °C, and then warmed to rt. The reaction was judged to be complete by TLC-analysis. 500 mL saturated solution of $NaHCO_3$ was added, stirred vigorously for 3 hr. The organic layer was separated, and the aqueous layer was extracted with 300 mL × 3 DCM. The combined organic layers were dried over $MgSO_4$, filtered, and the filtrate was concentrated in *vacuo*. The crude product was obtained as a 1.3:1 mixture of **ent-12** and **S4**. A subsequent purification by SiO_2 flash column chromatography afforded 5.4 g of the title compound **ent-12** (39% isolated yield, 61% combined yield of **ent-12** and **S4**) as yellow solid. Spectral data for **ent-12**: ^{1}H NMR (600 MHz, Chloroform-*d*) δ 7.25 (d, *J* = 8.7 Hz, 1H), 6.72 (dd, *J* = 8.7, 2.9 Hz, 1H), 6.57 (d, *J* = 2.7 Hz, 1H), 4.62 – 4.48 (m, 1H), 3.77 (s, 3H), 2.90 – 2.81 (m, 3H), 2.46 (dt, *J* = 13.3, 4.5 Hz, 1H), 2.42 – 2.34 (m, 1H), 2.29 (dd, *J* = 16.7, 5.1 Hz, 1H), 2.23 (ddd, *J* = 14.3, 5.6, 3.5 Hz, 1H), 2.02 (dd, *J* = 12.1, 6.5 Hz, 1H), 1.91 (ddd, *J* = 14.7, 12.3, 3.6 Hz, 1H), 1.57 (ddd, *J* = 12.9, 5.6, 3.5 Hz, 1H), 1.39 (s, 1H), 1.33 (s, 3H), 1.30 – 1.23 (m, 2H), 1.05 (s, 3H). ^{13}C NMR (150 MHz, Chloroform-*d*) δ 157.3, 138.9, 138.6, 137.1, 133.6, 126.4, 113.6, 112.1, 71.1, 55.3, 51.5, 41.3, 39.1, 38.1, 34.4, 34.2, 33.0, 31.9, 25.9, 24.9; IR (thin film): 3320, 2954, 2912, 2846, 1598, 1482, 1447, 1270, 1240, 1037 cm^{-1}; HRMS (ESI-TOF): calculated for $C_{20}H_{27}O_2$ $[M+H]^+$ 299.2013, found 299.2011; $[\alpha]_D^{22}$ = -117.2 (*c* 0.0086, $CHCl_3$). See Supplementary Figures 26 and 27 for ^{1}HNMR and ^{13}CNMR spectra.

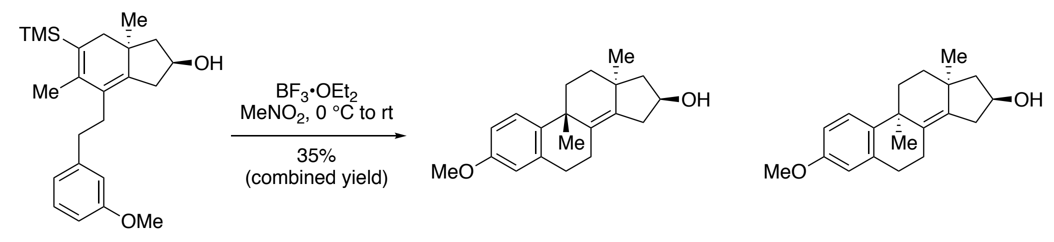

10 **11** 6 : **12** 1

Supplementary Figure 78. BF_3•OEt_2 Mediated Cyclization

Tetracycle 11 and 12: To a stirring solution of hydrindane **10** (0.39 g, 1.0 mmol, 1.0 equiv) in 20 mL $MeNO_2$ at 0 °C under N_2 atmosphere was added BF_3•OEt_2 (0.59 g, 4.2 mmol, 4.2 equiv). The resulting mixture was stirred for 3.3 hr at 0 °C, and then warmed to rt. The reaction mixture was stirred for another 2 hr and then quenched with 6 mL saturated solution of $NaHCO_3$. The resulting mixture was diluted with 25 mL dichloromethane, and the organic layer was separated. The aqueous layer was further extracted with 25 mL × 2 ethyl acetate. The combined organic layers were dried with Na_2SO_4, filtered, and the filtrate was concentrated in *vacuo*. The crude product was purified with SiO_2 flash column chromatography to afford 0.11 g of the title compound **11** and **12** as a yellow film (35% combined yield). See Supplementary Figures 28 and 29 for ^{1}HNMR analysis of the crude material.

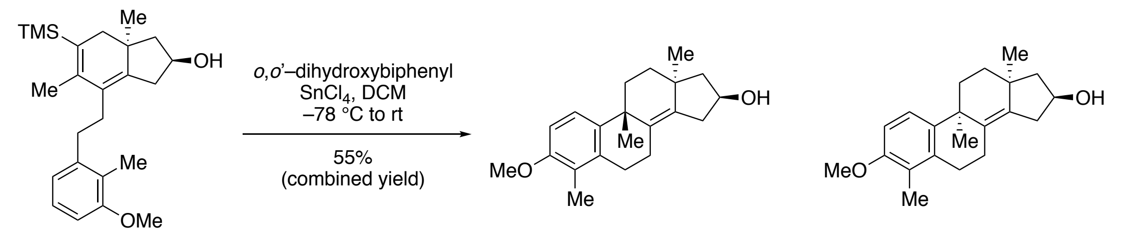

23 **24** 9 : **S5** 1

Supplementary Figure 79. *o,o'*-dihydroxybiphenyl/$SnCl_4$ Mediated Cyclization

Tetracycle 11 and 12: To a stirring solution of *o,o*–dihydroxybiphenyl (0.12 g, 0.64 mmol, 5.4 equiv) in 6 mL dichloromethane at –78 °C under N_2 atmosphere was added a solution of $SnCl_4$ (1.0 M in dichloromethane, 0.60 mL, 0.60 mmol, 5.0 equiv) dropwise using syringe. The resulting mixture was stirred for 30 min at –78 °C, and then a solution of **23** (47 mg, 0.12 mmol, 1.0 equiv) in 2 mL dichloromethane was added dropwise using syringe. The resulting mixture was stirred for 1.5 hr at –78 °C, and then warmed to rt over 9 min. At this point, the reaction was judged to be complete by TLC-analysis. The reaction was quenched with saturated solution of $NaHCO_3$, and then further diluted with dichloromethane. The organic layer was separated, and the aqueous layer was extracted with dichloromethane. The combined organic layers were dried over Na_2SO_4, filtered, and the filtrate was concentrated in *vacuo*. A subsequent purification by SiO_2 flash column chromatography afforded 21 mg of the title compound **24** and **S5** as an

amorphous white solid (55% combined yield). See Supplementary Figures 30 and 31 for ^{1}HNMR analysis of the crude material.

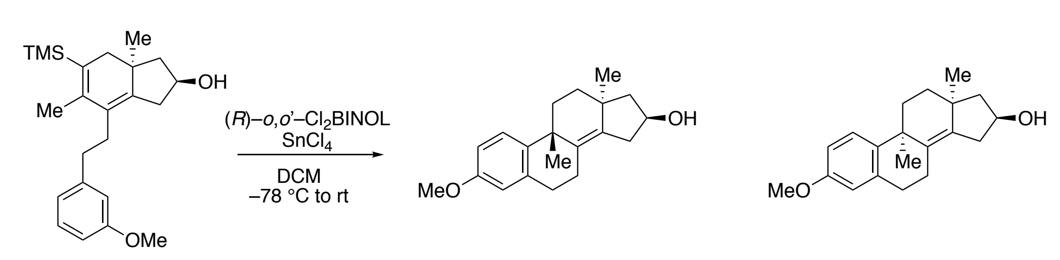

Supplementary Figure 80. (*R*)-*o,o'*-Cl_2BINOL/$SnCl_4$ Mediated Cyclization

Tetracycle 11 and 12: To a stirring solution of (*R*)–*o,o*–Cl_2BINOL[6] (64 mg, 0.18 mmol, 0.81 equiv) in 2 mL dichloromethane at –78 °C under N_2 atmosphere was added a solution of $SnCl_4$ (1.0 M in dichloromethane, 0.18 mL, 0.18 mmol, 0.81 equiv) dropwise using syringe. The resulting mixture was stirred for 30 min at –78 °C, and then a solution of **10** (80 mg, 0.22 mmol, 1.0 equiv) in 4 mL dichloromethane was added dropwise using syringe. The resulting mixture was stirred for 2.2 hr at –78 °C, and then warmed to rt. At this point, the reaction was judged to be complete by TLC-analysis. The reaction was quenched with 2 mL saturated solution of $NaHCO_3$, and then further diluted with dichloromethane. The organic layer was separated, and the aqueous layer was extracted with dichloromethane. The combined organic layers were dried over Na_2SO_4, filtered, and the filtrate was concentrated in *vacuo*. Yield not determined. See Supplementary Figures 32 and 33 for ^{1}HNMR analysis of the crude material.

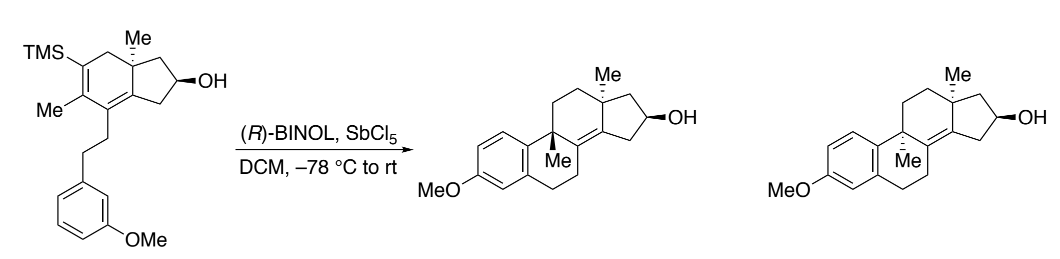

Supplementary Figure 81. (*R*)-BINOL/$SbCl_5$ Mediated Cyclization

Tetracycle 11 and 12: To a stirring solution (see supplementary note **1**) of (*R*)–BINOL (0.15 g, 0.52 mmol, 1.3 equiv) in 5 mL dichloromethane at –78 °C under N_2 atmosphere was added a solution of $SbCl_5$ (1.0 M in dichloromethane, 0.40 mL, 0.40 mmol, 1.0 equiv) dropwise using syringe. The resulting mixture was stirred for 20 min at –78 °C, and then a solution of **10** (0.15 g, 0.40 mmol, 1.0 equiv) in 7 mL dichloromethane was added dropwise using syringe. The resulting mixture was stirred for 30 min at –78 °C, and then warmed to rt. At this point, the reaction was judged to be complete by TLC-analysis. The reaction was quenched with 10 mL saturated solution of NH_4Cl, and then further diluted with 25 mL water. The organic layer was separated, and the aqueous layer was extracted with 30 mL dichloromethane. The combined organic layers

were dried over Na_2SO_4, filtered, and the filtrate was concentrated in *vacuo*. Yield not determined. See Supplementary Figures 34 and 35 for ^{1}HNMR analysis of the crude material.

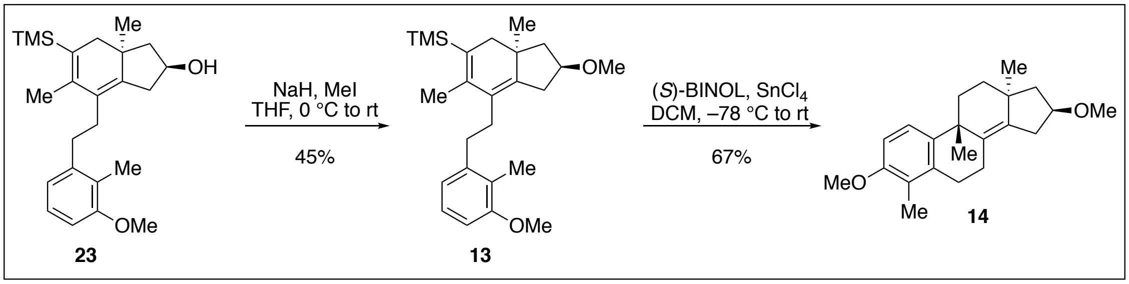

Supplementary Figure 82. Synthesis of Tetracycle 14

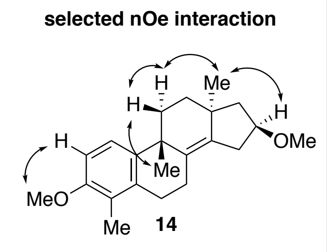

Supplementary Figure 83. Selected nOe Interaction

Tetracycle 14: To a stirring solution of **23** (0.12 g, 0.31 mmol, 1.0 equiv) in 1.5 mL THF at 0 °C under N_2 was added NaH (60% dispersion in mineral oil, 37 mg, 0.93 mmol, 3.0 equiv) followed by MeI (0.26 g, 1.8 mmol, 5.9 equiv). The resulting mixture was warmed to rt, and then stirred overnight (approx. 16 hr). 20 mL water was added to the reaction mixture, and then the organic layer was separated. The aqueous layer was extracted with 25 mL ethyl acetate, and the combined organic layers were dried over Na_2SO_4. The resulting suspension was filtered, and the filtrate was concentrated in *vacuo*. A subsequent purification by SiO_2 flash column chromatography afforded 56 mg of tentatively assigned hydrindane **13** (45% isolated yield).

To a stirring solution (see supplementary note **1**) of (*S*)-BINOL (0.22 g, 0.77 mmol, 5.5equiv) in 8 mL dichloromethane at –78 °C under N_2 atmosphere was added $SnCl_4$ (1.0 M in dichloromethane, 0.70 mL, 0.70 mol, 5.0 equiv). The resulting mixture was stirred for 30 min at –78 °C followed by a dropwise addition of of **13** (56 mg, 0.14 mmol, 1.0 equiv) in 3 mL dichloromethane, and then stirred for 1.5 hr at the same temperature. After this period, the reaction mixture was warmed to rt, and then quenched by a saturated solution of $NaHCO_3$. The organic layer was separated, and the aqueous layer was extracted with 25 mL dichloromethane. The combined organic layers were washed with 10 mL 10% solution of NaOH (w/w), dried over Na_2SO_4, and then filtered. The resulting filtrate was concentrated in *vacuo* to yield the crude product as a yellow film. A subsequent purification by SiO_2 flash column chromatography afforded 31 mg of the title compound **14** as an amorphous white solid (67% isolated yield, 30% yield over two steps). Spectral data for **14**: ^{1}H NMR (600 MHz, Chloroform-*d*) δ 7.14 (d, *J* = 8.6 Hz, 1H), 6.76 (d, *J* = 8.7 Hz, 1H), 4.13 (tdd, *J* = 8.5, 6.7, 4.4 Hz, 1H), 3.80 (s, 3H), 3.33 (s, 3H), 2.95 – 2.88 (m, 1H), 2.77 (ddd, *J* = 16.9, 8.8, 1.6 Hz, 1H), 2.55 – 2.46 (m, 2H), 2.38 – 2.32 (m, 2H), 2.15 (dd, *J* = 11.7, 6.7 Hz, 1H), 2.12 – 2.07 (m, 4H), 1.83 – 1.76 (m, 1H), 1.72 – 1.68 (m, 2H), 1.36 – 1.30 (m, 4H), 0.88 (s, 3H); ^{13}C NMR (150 MHz, Chloroform-*d*) δ 155.0, 140.5, 136.0, 135.7, 131.5,

124.1, 123.7, 108.7, 80.4, 56.9, 55.7, 48.7, 41.2, 38.3, 34.9, 34.2, 33.5, 31.5, 29.6, 25.8, 24.7, 11.5; IR (thin film): 2933, 2831, 1595, 1482, 1464, 1373, 1264, 1119, 1101, 802 cm^{-1}; HRMS (ESI–TOF): calculated for $C_{22}H_{31}O_2$ $[M+H]^+$ 327.2324, found 327.2315. $[\alpha]_D^{23}$ = –203.4 (*c* 0.010 $CHCl_3$). See Supplementary Figures 38 and 39 for ^{1}HNMR and ^{13}CNMR spectra.

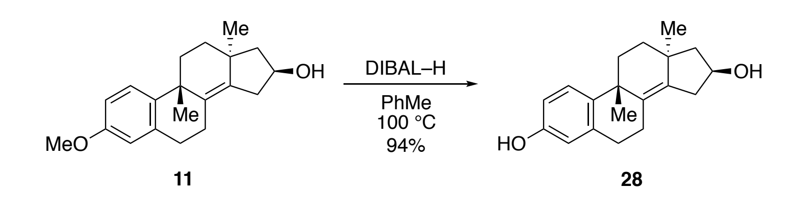

Supplementary Figure 84. Synthesis of Phenol **28**

Phenol 28: To a stirring solution of **11** (0.17 g, 0.57 mmol, 1.0 equiv) in 5 mL anhydrous toluene at rt under N_2 atmosphere was added DIBAL-H (1.0 M in hexanes, 5.7 mL, 5.7 mmol, 10 equiv). The resulting mixture was warmed to 100 °C, refluxed overnight (approx. 20 hr), and then cooled to rt. Small chunks of ice was slowly added, and the resulting mixture was acidified with 3M aqueous hydrochloric acid (3 mL). The organic layer was separated, and the aqueous layer was extracted with 50 mL × 3 ethyl acetate. The combined organic layers were dried over Na_2SO_4, filtered, and then the filtrate was concentrated in *vacuo*. The crude product was purified with SiO_2 flash column chromatography to afford 0.15 g of the title compound **28** as an amorphous white solid (94% isolated yield). Spectral Data for **28**: ^{1}H NMR (500 MHz, Methanol-d_4) δ 7.09 (d, *J* = 8.6 Hz, 1H), 6.60 (dd, *J* = 8.5, 2.7 Hz, 1H), 6.44 (d, *J* = 2.6 Hz, 1H), 4.53 (tdd, *J* = 8.6, 6.7, 4.5 Hz, 1H), 2.87 – 2.77 (m, 2H), 2.61 (ddd, *J* = 15.9, 11.8, 5.7 Hz, 1H), 2.41 (ddd, *J* = 12.8, 5.7, 2.5 Hz, 1H), 2.32 (td, *J* = 13.4, 12.8, 5.1 Hz, 1H), 2.23 (dd, *J* = 16.9, 4.5 Hz, 1H), 2.12–2.04 (m, 2H), 1.79 (td, *J* = 12.8, 4.0 Hz, 1H), 1.74–1.63 (m, 2H), 1.34–1.26 (m, 4H), 0.89 (s, 3H); ^{13}C NMR (150 MHz, Methanol-d_4) δ 155.4, 140.0, 138.0, 137.5, 133.2, 128.1, 115.4, 114.6, 71.7, 52.4, 42.5, 39.0, 38.0, 35.5, 34.4, 33.0, 31.7, 26.1, 26.0; IR (thin film): 3314, 2932, 2857, 1608, 1497, 1279, 1238, 737, 443 cm^{-1}; HRMS (ESI–TOF): Calculated for $C_{19}H_{25}O_2$ $[M+H]^+$ 285.1855, found 285.1847; $[\alpha]_D^{23}$ = –229.4 (*c* 0.024, MeOH). See Supplementary Figures 40 and 41 for ^{1}HNMR and ^{13}CNMR spectra.

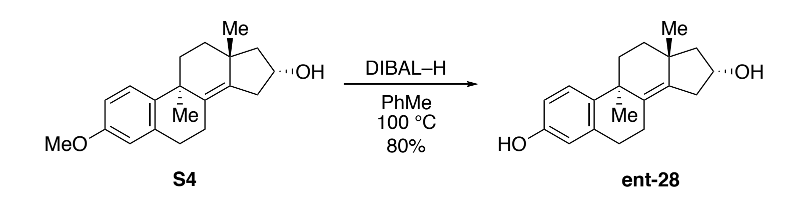

Supplementary Figure 85. Synthesis of Tetracycle **ent-28**

Tetracycle ent-28: To a stirring solution of **S4** (0.40 g, 1.3 mmol, 1.0 equiv) in 13 mL anhydrous toluene at rt under N_2 atmosphere was added DIBAL-H (1.0 M in toluene, 13 mL, 13 mmol, 10 equiv). The resulting mixture was warmed to 100 °C, refluxed overnight (approx. 20 hr), and then cooled to rt. The solution was slowly quenched with a saturated aqueous solution of Rochelle's salt, and the resulting mixture was stirred until biphasic and the organic layer was separated. The aqueous layer was acidified with 1M aqueous hydrochloric acid and extracted with 50 mL × 3 ethyl acetate. The combined organic layers were dried over $MgSO_4$, filtered, and then the filtrate was concentrated in *vacuo* and purified by SiO_2 flash column chromatography to afford 0.30 g of **ent-28** as a yellow amorphous solid (80 % isolated yield). Spectral data for **ent-28**: ^{1}H NMR (600 MHz, Methanol-d_4) δ 7.11 (d, *J* = 8.6 Hz, 1H), 6.60 (dd, *J* = 8.3, 2.6 Hz, 1H), 6.45 (d, *J* = 2.6 Hz, 1H), 4.55 (dt, *J* = 8.8, 3.0 Hz, 1H), 2.88 – 2.76 (m, 2H), 2.67 – 2.58 (m, 1H), 2.47 – 2.40 (m, 1H), 2.36 (dd, *J* = 12.8, 5.4 Hz, 1H), 2.24 (dd, *J* = 17.0, 4.5 Hz, 1H), 2.14 – 2.08 (m, 2H), 1.81 (td, *J* = 13.1, 3.8 Hz, 1H), 1.76 – 1.66 (m, 2H), 1.34 – 1.29 (m, 4H), 0.92 (s, 3H). ^{13}C NMR (150 MHz, Methanol-d_4) δ 155.5, 140.0, 138.0, 137.6, 133.3, 128.1, 115.4, 114.6, 71.7, 52.4, 42.5, 39.1, 38.0, 35.5, 34.4, 33.0, 31.7, 26.1, 26.0; IR (thin film): 3389, 2932, 2723, 2518, 1650, 1608, 1587, 1497, 1452, 1280, 1235, 1132, 1019, 919, 814; HRMS (EI-TOF): calculated for $C_{19}H_{25}O_2$ $[M+H]^+$ 285.1855 found 285.1842; $[\alpha]_D^{22}$ = +184.8 (*c* 0.0034, MeOH). See Supplementary Figures 42 and 43 for ^{1}HNMR and ^{13}CNMR spectra.

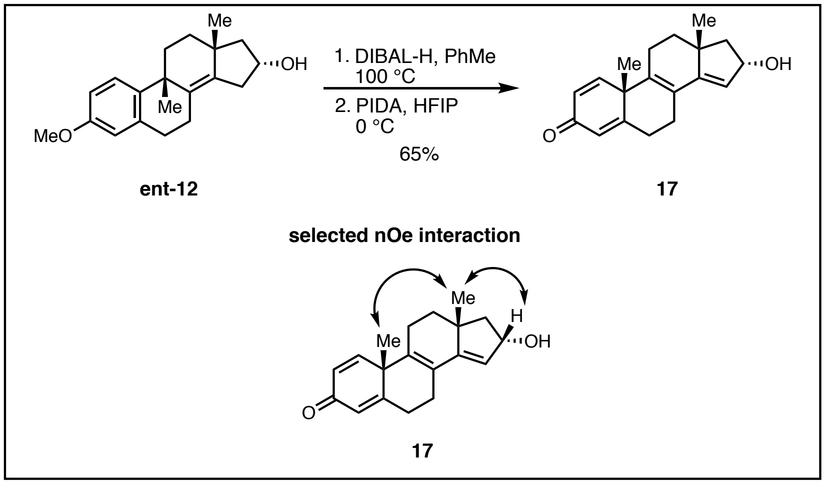

Supplementary Figure 86. Synthesis of **Enone 17**

Enone 17: To a stirring solution of **ent-12** (0.89 g, 2.9 mmol, 1.0 equiv) in 34 mL anhydrous toluene at rt under N_2 atmosphere was added DIBAL-H (1.0 M in toluene, 34 mL, 34 mmol, 12 equiv). The resulting mixture was warmed to 100 °C, refluxed overnight (approx. 20 hr), and then cooled to rt. The solution was slowly quenched with a saturated aqueous solution of Rochelle's salt, and the resulting mixture was stirred until biphasic and the organic layer was separated. The aqueous layer was acidified with 1M aqueous hydrochloric acid and extracted with 200 mL × 3 ethyl acetate. The combined organic layers were dried over $MgSO_4$, filtered, and then the filtrate was concentrated in *vacuo* to afford 1.2 g of the crude product as an amorphous yellow solid. The crude product was then dissolved in 45 mL HFIP and cooled to 0 °C under N_2 atmosphere, and stirred for 20 min. PIDA (1.3 g, 4.2 mmol, 0.95 eqiv) was added to the reaction mixture, stirred for 1 min at the same temperature (until PIDA is fully dissolved), and then 30 mL saturated solution of $NaHCO_3$ was added. HFIP was subsequently removed from the reaction mixture under vacuum, diluted with 75 mL ethyl acetate, and the organic layer was separated. The aqueous layer was extracted with 3 × 100 mL ethyl acetate, and the organic layers were combined. The combined organic layers were dried over $MgSO_4$, filtered, and the filtrate was concentrated in *vacuo*. The crude product was purified with SiO_2 flash column chromatography to afford 0.55 g of the title compound **17** as an amorphous tan solid (65% isolated yield over two steps). Spectral data for **17**: ^{1}H NMR (600 MHz, Chloroform-*d*) δ 7.21 (d, *J* = 10.2 Hz, 1H), 6.26 (dd, *J* = 10.2, 1.9 Hz, 1H), 6.14 (t, *J* = 1.7 Hz, 1H), 5.45 (d, *J* = 1.7 Hz, 1H), 5.09 – 5.01 (m, 1H), 2.80 – 2.71 (m, 1H), 2.58 – 2.47 (m, 4H), 2.34 (app td, *J* = 11.0, 9.8, 6.7 Hz, 2H), 1.81 (ddd, *J* = 12.7, 5.7, 1.8 Hz, 1H), 1.61 (s, 1H), 1.54 (td, *J* = 12.2, 5.9 Hz, 1H), 1.46 (s, 3H), 1.37 (dd, *J* = 12.4, 7.5 Hz, 1H), 1.00 (s, 3H). ^{13}C NMR (150 MHz, Chloroform-*d*) δ 185.8, 166.3, 153.5, 150.4, 137.2, 128.1, 125.5, 124.3, 123.7, 76.5, 51.6, 44.7, 43.4, 35.9, 30.1, 29.9, 29.0, 24.1, 23.8; IR (thin film): 3376, 2958, 2914, 2854, 1743, 1722, 1701, 1660, 1548 cm^{-1}; HRMS (ESI-TOF): calculated for $C_{19}H_{22}O_2$ $[M+H]^+$ 283.1686, found 283.1698; $[\alpha]_D^{22}$ = -153.3 (*c* 0.008, $CHCl_3$). See Supplementary Figures 44 and 45 for ^{1}HNMR and ^{13}CNMR spectra.

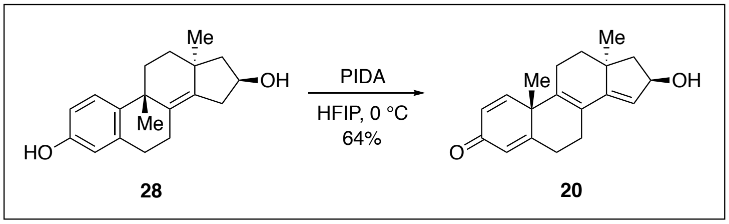

Supplementary Figure 87. Synthesis of Enone 20

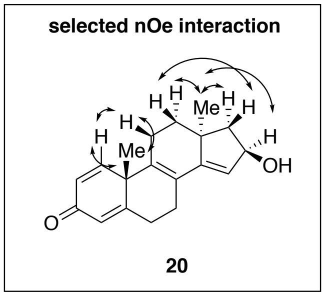

Supplementary Figure 88. Selected nOe interaction

Enone 20[7]: To a round bottom flask charged with **28** (63 mg, 0.22 mmol, 1.0 equiv) at 0 °C under N_2 atmosphere was added 2 mL HFIP, and the resulting mixture was stirred for 6 min. PIDA (70 mg, 0.22 mmol, 1.0 equiv) was added, stirred for 1 min at the same temperature (PIDA was fully dissolved at this point), and then 1 mL saturated solution of $NaHCO_3$ was added. The resulting mixture was further diluted with 20 mL ethyl acetate, and the organic layer was separated. The aqueous layer was extracted with 15 mL ethyl acetate, and then the combined organic layers were dried over Na_2SO_4 and then filtered. The filtrate was concentrated in *vacuo*. The crude product was purified with SiO_2 flash column chromatography to afford 40 mg of the title compound **20** as an amorphous white solid (64% isolated yield) (see supplementary note **2**). (Caution: Prolonged stirring of more than 30 min after PIDA addition resulted in a significantly lower yield). Spectral Data for **5**: ^{1}H NMR (500 MHz, Chloroform-*d*) δ 7.16 (d, *J* = 10.1 Hz, 1H), 6.24 (dd, *J* = 10.1, 1.9 Hz, 1H), 6.13 (s, 1H), 5.50 (s, 1H), 5.09 – 5.01 (m, 1H), 2.78 – 2.67 (m, 2H), 2.55 – 2.50 (m, 1H), 2.46 – 2.39 (m, 1H), 2.32 (app dd, *J* = 12.1, 6.5 Hz, 2H), 2.28 – 2.17 (m, 1H), 1.86 – 1.78 (m, 2H), 1.57 (td, *J* = 12.4, 5.5 Hz, 1H), 1.46 (s, 3H), 1.40 (dd, *J* = 12.1, 7.6 Hz, 1H), 0.88 (s, 3H); ^{13}C NMR (150 MHz, Chloroform-*d*) δ 185.9, 166.6, 152.6, 149.5, 136.9, 128.2, 125.1, 124.7, 123.6, 76.4, 51.5, 44.7, 43.2, 36.1, 29.7, 29.0, 28.6, 24.5, 23.5; IR (thin film): 3388, 2952, 2923, 2851, 1662, 1624, 1057, 887, 731 cm^{-1}; HRMS (ESI–TOF): Calculated for $C_{19}H_{23}O_2$ $[M+H]^+$ 283.1698, found 283.1693; $[\alpha]_D^{23}$ = +128.0 (*c* 0.042, $CHCl_3$). See Supplementary Figures 46 and 47 for ^{1}HNMR and ^{13}CNMR spectra.

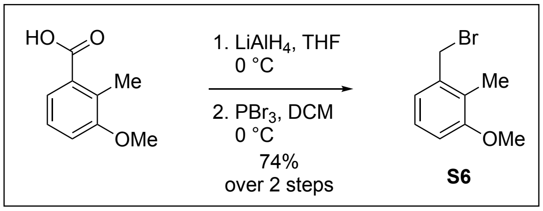

Supplementary Figure 89. Synthesis of **S6**

Benzyl Bromide S6: To a stirring suspension of $LiAlH_4$ (25 g, 0.66 mol, 1.1 equiv) in 1.3 L THF at 0 °C under N_2 was added 3-methoxy-2-methylbenzoic acid (100 g, 0.60 mol, 1.0 equiv) portion wise over 30 min (3 to 12 g/portion) (caution: violent H_2 evolution). After the addition was complete, the ice bath was removed, and the resulting mixture was stirred for 2.5 hr at rt. After this period, the reaction mixture was cooled to 0 °C, 28 mL water was added, and then stirred for 20 min. 83 mL 5% (w/w) NaOH (aq) solution was added to the reaction mixture at 0 °C, the cooling bath was removed, and then stirred for 13 min. After sitting at rt without stirring for another 13 min, the mixture was further diluted with 0.85 L Et_2O, and then the organic layer was separated. The aqueous suspension was further extracted with 1 L Et_2O, separated, and then the aqueous suspension was filtered. After the filtration, the filter cake was washed with 500 mL × 2 Et_2O. The combined organic layers were dried with Na_2SO_4, filtered, and then the filtrate was concentrated in *vacuo*. The crude product was obtained as a white solid (90 g), and then used without further purification.

To a stirring solution of the above crude product (32 g) in 600 mL dichloromethane at 0 °C was added PBr_3 (20 g, 74 mmol) dropwise using a syringe. The resulting mixture was stirred at 0 °C until the reaction was judged to be complete by TLC-analysis, and then 100 mL water was added at the same temperature. The resulting mixture was warmed to rt, and the organic layer was separated. The aqueous layer was further extracted with 400 mL dichloromethane, and the resulting organic layers were combined. The combined organic layers were dried with Na_2SO_4, filtered, and the filtrate was concentrated in *vacuo*. The remaining residue was dissolved in 500 mL pentane, and then passed through a pad of silica gel using 1 L pentane and 500 mL 5% ethyl acetate in pentane as the eluent. The combined filtrate was concentrated in *vacuo* to yield benzyl bromide **S6** as a pale yellow solid (34 g, 74% yield over two steps). Spectral data for **S6**: ^{1}H NMR (600 MHz, Chloroform-*d*) δ 7.16 (t, *J* = 7.9 Hz, 1H), 6.96 (d, *J* = 7.6 Hz, 1H), 6.84 (d, *J* = 8.2 Hz, 1H), 4.54 (s, 2H), 3.84 (s, 3H), 2.29 (s, 3H); ^{13}C NMR (151 MHz, Chloroform-*d*) δ 158.1, 137.0, 126.6, 126.2, 122.2, 110.8, 55.7, 32.6, 11.1; IR (thin film): 3000, 2935, 2834, 1585, 1472, 1439, 1311, 1261, 1211, 1098 cm^{-1}; HRMS (ESI-TOF) calculated for $C_9H_{12}BrO$ $[M+H]^+$ 215.0072, found 215.0072). See Supplementary Figures 48 and 49 for ^{1}HNMR and ^{13}CNMR spectra.

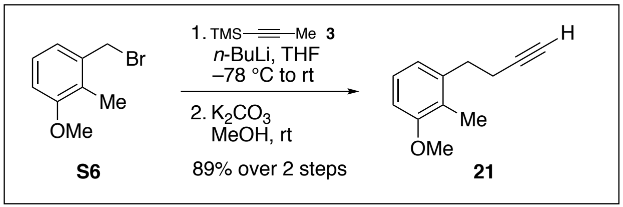

Supplementary Figure 90. Synthesis of Alkyne **21**

Alkyne 21: To a stirring solution of 1-(trimehtylsilyl)propyne (**3**) (6.5 g, 58 mmol, 1.5 equiv) in THF at –78 °C under N_2 atmosphere was added *n*-BuLi (2.5 M in hexanes, 19 mL, 48 mmol, 1.3 equiv) dropwise, and the mixture was stirred for 1.2 hr at the same temperature. A solution **S6** (7.9 g, 36 mmol, 1.0 equiv) in 10 mL THF in a separate round bottom flask was added dropwise to the reaction mixture followed by a 10 mL THF rinse, and the resulting mixture was stirred for 26 min at –78 °C under N_2 atmosphere. The reaction mixture was warmed to rt, stirred for 24 min, and then 50 mL water was added. The org layer was separated, and the aqueous layer was extracted with 250 mL × 2 ethyl acetate. The combined organic layers were dried over Na_2SO_4, filtered, and concentrated in *vacuo*. The crude oil (9.6 g) was dissolved in 100 mL MeOH, and K_2CO_3 (25 g, 183 mmol, 5.1 equiv) was added. The resulting suspension was stirred at rt under N_2 atmosphere until the reaction was judged to be complete *via* TLC-analysis. The suspension was filtered, and the filtrate was concentrated in *vacuo*. The crude oil was dissolved in 100 mL hexanes, passed through a pad of silica using 100 mL × 4 hexanes as the eluent, and then concentrated in *vacuo* to afford **21** as a clear oil (5.7 g, 89% over 2 steps). Spectral data for **21**:
^{1}H NMR (600 MHz, Chloroform-*d*): δ 7.12 (t, *J* = 7.9 Hz, 1H), 6.82 (d, *J* = 7.7 Hz, 1H), 6.75 (d, *J* = 8.2 Hz, 1H), 3.82 (s, 3H), 2.88 (t, *J* = 7.8 Hz, 2H), 2.49 – 2.37 (m, 2H), 2.20 (s, 3H), 2.05 – 1.95 (m, 1H); ^{13}C NMR (150 MHz, Chloroform-*d*): δ 157.9, 140.0, 126.2, 124.8, 121.5, 108.6, 84.1, 68.9, 55.7, 32.8, 19.7, 11.4; IR (thin film): 3293, 2998, 2938, 2835, 2117, 1586, 1471, 1439, 1258, 1102 cm^{-1}; HRMS (ESI-TOF): calculated for $C_{12}H_{14}O$ [M^+] 174.1045, found 174.1049**.** See Supplementary Figures 50 and 51 for ^{1}HNMR and ^{13}CNMR spectra.

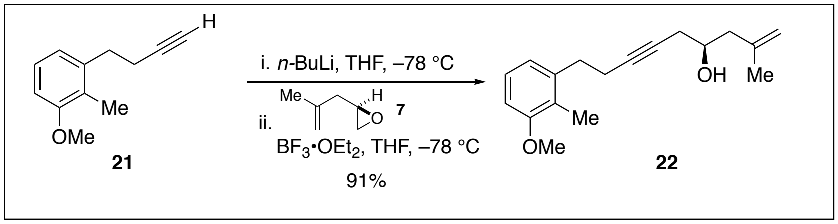

Supplementary Figure 91. Synthesis of Enyne **22**

Enyne 22: To a stirring solution of **21** (5.4 g, 31 mmol, 2.0 equiv) in 90 mL THF at –78 °C under N_2 atmosphere was added *n*-BuLi (2.5 M in hexanes, 10 mL, 25 mmol, 1.6 equiv) dropwise over 3 min, and the resulting mixture was stirred for 30 min at the same temperature. After the specified period of time, $BF_3•OEt_2$ (4.0 g, 28 mmol, 1.8 equiv) was added dropwise to the reaction mixture followed by **7**[5] (1.5g, 15 mmol, 1.0 equiv). The resulting mixture was stirred at –78 °C under N_2 atmosphere for 55 min, and then quenched with 50 mL saturated solution of $NaHCO_3$ at the same temperature. The resulting biphasic solution was warmed to rt, and then further diluted with 50 mL ethyl acetate. The organic layer was separated, dried over Na_2SO_4, filtered, and then the filtrate was concentrated in *vacuo*. SiO_2 flash column chromatography afforded the title compound **22** as a clear oil (3.9 g, 91%); Spectral Data for **22**: ^{1}H NMR (600 MHz, Chloroform-*d*): δ 7.13 (t, *J* = 7.9 Hz, 1H), 6.85 (d, *J* = 1.1 Hz, 1H), 6.76 (d, *J* = 1.1 Hz, 1H), 4.92 – 4.86 (m, 1H), 4.86 – 4.80 (m, 1H), 3.89 – 3.84 (m, 1H), 3.83 (s, 3H), 2.87 (t, *J* = 7.7 Hz, 2H), 2.47 (tt, *J* = 7.6, 2.4 Hz, 2H), 2.43 – 2.33 (m, 2H), 2.29 (ddd, *J* = 13.9, 4.9, 1.2 Hz, 1H), 2.25 – 2.18 (m, 4H), 2.16 (s, 1H), 1.79 (s, 3H); ^{13}C NMR (150 MHz, Chloroform-*d*): δ 157.7, 142.4, 140.1, 126.0, 124.5, 121.4, 113.3, 108.3, 82.3, 76.8, 67.8, 55.4, 44.7, 32.9, 27.2, 22.5, 19.8, 11.2; IR (thin film): 3441, 2933, 2835, 1585, 1463, 1439, 1258, 1101 cm^{-1} ;HRMS (ESI–TOF) calculated for $C_{18}H_{25}O_2$ $[M+H]^+$ 273.1855, found 273.1855; $[\alpha]_D^{22}$ = –1.1 (0.05, $CHCl_3$). See Supplementary Figures 52 and 53 for ^{1}HNMR and ^{13}CNMR spectra.

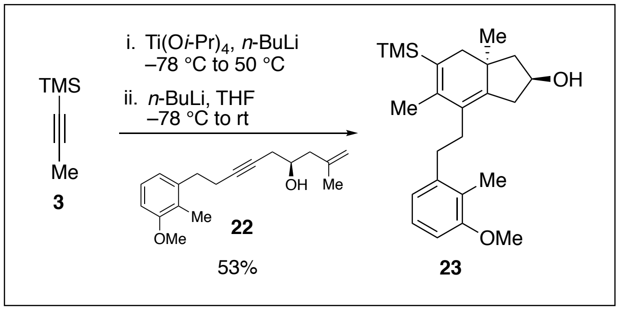

Supplementary Figure 92. Synthesis of Hydrindane **23**

Hydrindane 23: To a stirring solution of 1-(Trimethylsilyl)propyne (**3**) (4.5 g, 40 mmol, 3.1 equiv) and Ti(O*i*-Pr)$_4$ (11 g, 38 mmol, 3.0 equiv) in 200 mL anhydrous toluene at –78 °C under N_2 atmosphere was added *n*-BuLi (2.3 M in hexanes, 33 mL, 76 mmol, 5.9 equiv) dropwise. After the addition, the cooling bath was removed, and the resulting dark brown mixture was warmed to rt, and then further warmed to 50 °C. The reaction mixture was stirred for 50 min at the same temperature without a reflux condenser, and then cooled to rt. A separate round bottom flask charged with a solution of **22** (3.5 g, 13 mmol, 1.0 equiv) in 50 mL anhydrous toluene at –78 °C under N_2 atmosphere was added *n*-BuLi (2.3 M in hexanes, 5.5 mL, 13 mmol, 1.0 equiv) dropwise. The resulting solution was warmed to rt, cannulated into the above dark brown mixture, and then stirred overnight at rt under N_2 atmosphere (approx. 12 hr). After this period, 100 mL saturated solution of NH_4Cl was added, and the mixture was further diluted with 100 mL ethyl acetate. The organic layer was separated, and the aqueous layer was extracted with 250 mL × 2 ethyl acetate. The combined organic layers were dried with Na_2SO_4, filtered, and the filtrate was concentrated in vacuo. SiO_2 flash column chromatography afforded the title compound **23** as a yellow oil (2.6 g, 53% isolated yield); Spectral Data for **23**: ^{1}H NMR (500 MHz, Chloroform-*d*): δ 7.07 (t, *J* = 7.9 Hz, 1H), 6.71 (d, *J* = 7.9 Hz, 2H), 4.41 – 4.31 (m, 1H), 3.81 (s, 3H), 2.73 – 2.62 (m, 2H), 2.57 (dt, *J* = 13.5, 8.0 Hz, 1H), 2.44 – 2.37 (m, 1H), 2.35 – 2.27 (m, 1H), 2.20 (s, 3H), 2.16 (d, *J* = 15.5 Hz, 1H), 2.05 – 1.97 (m, 3H), 1.95 (app d, *J* = 2.7 Hz, 3H), 1.40 (dd, *J* = 12.4, 7.5 Hz, 1H), 1.20 (s, 1H), 0.79 (s, 3H), 0.16 (s, 9H); ^{13}C NMR (150 MHz, Chloroform-*d*): δ 157.8, 144.4, 141.8, 141.3, 129.5, 128.5, 126.0, 124.8, 122.3, 108.0, 72.1, 55.7, 51.3, 41.6, 39.4, 38.5, 33.2, 30.5, 21.4, 19.2, 11.4, 0.2; IR (thin film): 3349, 2950, 1585, 1465, 14371102, 1063, 1063, 835 cm^{-1}; HRMS (ESI-TOF): calculated for $C_{24}H_{37}O_2Si$ $[M+H]^+$ 385.2563, found 385.2563; $[\alpha]_D^{22}$ = –39.3 (*c* 0.014, $CHCl_3$). See Supplementary Figures 54 and 55 for ^{1}HNMR and ^{13}CNMR spectra.

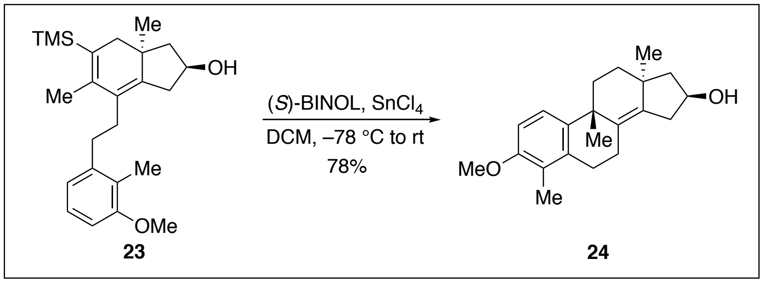

Supplementary Figure 93. Synthesis of Tetracycle **24**

Tetracycle 24 (See Supplementary Note 3)**:** To a stirring suspension (see supplementary note **1**) of (*S*)–BINOL (6.1 g, 21 mmol, 5.3 equiv) in 200 mL dichloromethane at –78 °C under N_2 atmosphere was added a solution of $SnCl_4$ (1.0 M in dichloromethane, 21 mL, 21 mmol, 5.3 equiv) dropwise using syringe. The resulting mixture was stirred for 29 min at –78 °C, and then a solution of **23** (1.5 g, 4.0 mmol, 1.0 equiv) in 80 mL dichloromethane was added dropwise *via* cannula transfer. The resulting mixture was stirred for 1.3 hr at –78 °C, and then warmed to rt over 30 min. The reaction was judged to be complete by TLC-analysis, and 100 mL saturated solution of NH_4Cl was added. The organic layer was separated, washed with 200 mL 5% solution of NaOH. The aqueous layer was further extracted with 200 mL dichloromethane. The combined organic layers were dried over Na_2SO_4, filtered, and the filtrate was concentrated in *vacuo*. SiO_2 flash column chromatography afforded the title compound **24** as an amorphous white solid (0.96 g, 78%); Spectral Data for **24**: ^{1}H NMR (600 MHz, Chloroform-*d*) δ 7.14 (d, *J* = 8.7 Hz, 1H), 6.77 (d, *J* = 8.7 Hz, 1H), 4.63 – 4.57 (m, 1H), 3.80 (s, 3H), 2.92 (dd, *J* = 14.5, 5.7 Hz, 1H), 2.85 (dd, *J* = 16.7, 8.7 Hz, 1H), 2.54 – 2.46 (m, 2H), 2.38 – 2.27 (m, 2H), 2.16 (dd, *J* = 11.9, 6.7 Hz, 1H), 2.13 – 2.05 (m, 4H), 1.85 – 1.78 (m, 1H), 1.71 (app dt, *J* = 9.3, 3.1 Hz, 2H), 1.57 (br s, 1H), 1.38 – 1.32 (m, 4H), 0.89 (s, 3H); ^{13}C NMR (150 MHz, Chloroform-*d*) δ 155.0, 140.5, 135.9, 131.8, 124.1, 123.7, 108.7, 71.7, 55.7, 52.1, 41.5, 38.3, 37.7, 35.0, 33.6, 31.6, 29.7, 25.9, 24.8, 11.5; IR (thin film): 3344, 2934, 2856, 2833, 1595, 1482, 1263, 1101, 755; HRMS (ESI-TOF): calculated for $C_{21}H_{29}O_2$ $[M+H]^+$ 313.2168, found 313.2162; $[\alpha]_D^{22}$ = –169.71 (*c* 0.0046, $CHCl_3$). See Supplementary Figures 58 and 59 for ^{1}HNMR and ^{13}CNMR spectra.

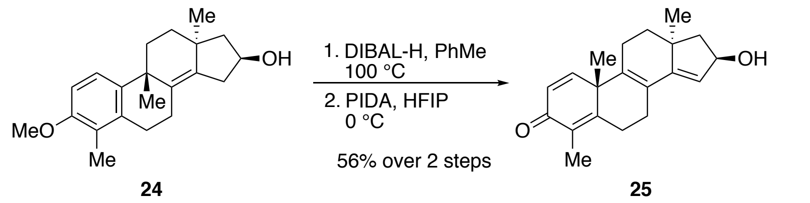

Supplementary Figure 94. Synthesis of Enone **25**

Enone 25: To a stirring solution of **24** (1.5 g, 4.7 mmol, 1.0 equiv) in 50 mL anhydrous toluene at rt under N_2 atmosphere was added DIBAL-H (1.0 M in hexanes, 47 mL, 47 mmol, 10 equiv). The resulting mixture was warmed to 100 °C, refluxed overnight (approx. 16 hr), and then cooled to rt. Small chunks of ice was slowly added, and the resulting mixture was diluted with 200 mL water, 10 mL 1 M solution of HCl, and 250 mL ethyl acetate. The separated organic layer was dried with Na_2SO_4, filtered, and concentrated in *vacuo* to afford 1.41 g of the crude product as an amorphous yellow solid. The crude product (1.4 g) at 0 °C under N_2 atmosphere was added 50 mL HFIP followed by PIDA (1.4 g, 4.3 mmol). The resulting mixture was stirred for 1 min at 0 °C (PIDA was fully dissolved at this point), and then 30 mL saturated solution of $NaHCO_3$ was added. HFIP was removed in *vacuo*, and the remaining aqueous mixture was extracted with 100 mL × 3 ethyl acetate. The combined organic layers were dried over Na_2SO_4, filtered, and the filtrate was concentrated in *vacuo*. The crude product was purified with SiO_2 flash column chromatography to afford the title compound **25** as an amorphous white solid (0.76 g, 54% over 2 steps). Spectral data for **25**: ^{1}H NMR (500 MHz, Chloroform-*d*) δ 7.14 (d, *J* = 10.1 Hz, 1H), 6.28 (d, *J* = 10.1 Hz, 1H), 5.50 (s, 1H), 5.11 – 5.02 (m, 1H), 2.97 (ddd, *J* = 13.0, 6.0, 1.7 Hz, 1H), 2.71 (ddt, *J* = 16.5, 6.3, 2.0 Hz, 1H), 2.53 – 2.39 (m, 2H), 2.34 (app dd, *J* = 12.1, 6.5 Hz, 2H), 2.18 (ddtd, *J* = 18.6, 10.3, 4.1, 2.0 Hz, 1H), 1.95 (s, 3H), 1.82 (ddd, *J* = 12.6, 5.0, 1.4 Hz, 1H), 1.64 (s, 1H), 1.57 (td, *J* = 12.4, 5.6 Hz, 2H), 1.45 (s, 3H), 1.40 (dd, *J* = 12.1, 7.6 Hz, 1H), 0.88 (s, 3H); ^{13}C NMR (150 MHz, Chloroform-*d*) δ 185.4, 159.4, 151.9, 149.9, 137.9, 128.3, 127.6, 125.4, 124.3, 76.6, 51.7, 44.8, 43.3, 36.2, 28.8, 28.4, 25.4, 24.7, 23.6, 10.5; IR (thin film): 3404, 2923, 2851, 1659, 1625,1607, 1449, 1051, 833, 753 cm^{-1}; HRMS (ESI-TOF): calculated for $C_{20}H_{25}O_2[M+H]^+$ 297.1855, found 297.1856; $[\alpha]_D^{22}$ = +156.27 (*c* 0.0032, $CHCl_3$). See Supplementary Figures 60 and 61 for ^{1}HNMR and ^{13}CNMR spectra.

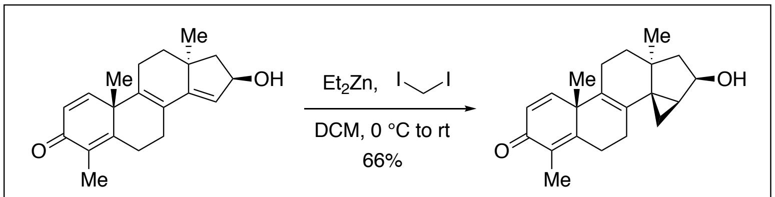

Supplementary Figure 95. Synthesis of Cyclopropane **S7**

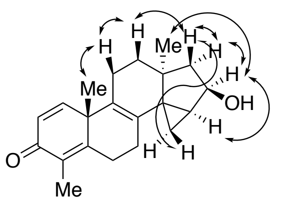

Supplementary Figure 96. Selected nOe interaction

Cyclopropane S7: To a stirring solution of diiodomethane (2.2 g, 8.3 mmol, 3.5 equiv) in 42 mL dichloromethane at 0 °C under N_2 atmosphere was added Et_2Zn (1.0 M in hexanes, 7.7 mL, 7.7 mmol, 3.2 equiv). After stirring for 12 min at the same temperature, a solution of **25** (0.71 g, 2.4 mmol, 1.0 equiv) in 9 mL dichloromethane was added dropwise to the reaction mixture and stirred for 20 min. The resulting mixture was warmed to rt, continued to stir for 1.4 hr, and then quenched with saturated solution of NH_4Cl. The resulting mixture was further diluted with 200 mL ethyl acetate, and the organic layer was separated. The aqueous layer was extracted with 100 mL × 2 ethyl acetate. The combined organic layers were dried over Na_2SO_4, filtered, and the filtrate was concentrated in *vacuo*. SiO_2 flash column chromatography afforded the title compound **S7** as an amorphous white solid (0.49 g, 66% isolated yield) as a single diastereomer; Spectral data for **S7**: ^{1}H NMR (600 MHz, Chloroform-*d*): δ 7.17 (d, *J* = 10.1 Hz, 1H), 6.25 (d, *J* = 10.1 Hz, 1H), 4.63 (dt, *J* = 7.9, 3.8 Hz, 1H), 2.87 (ddd, *J* = 13.0, 5.7, 2.0 Hz, 1H), 2.45 – 2.40 (m, 1H), 2.36 (td, *J* = 12.6, 7.0 Hz, 1H), 2.32 – 2.24 (m, 1H), 1.97 – 1.88 (m, 5H), 1.88 – 1.82 (m, 1H), 1.72 (td, *J* = 11.6, 6.9 Hz, 1H), 1.66 – 1.61 (m, 2H), 1.39 (s, 3H), 0.96 (dd, *J* = 12.4, 8.6 Hz, 1H), 0.83 (dd, *J* = 5.2, 3.2 Hz, 1H), 0.81 (s, 3H), 0.41 (dd, *J* = 7.4, 5.2 Hz, 1H); ^{13}C NMR (150 MHz, Chloroform-*d*): δ 185.6, 160.2, 153.1, 130.8, 130.5, 127.9, 127.0, 73.6, 44.4, 41.3, 38.9, 37.9, 31.9, 29.0, 28.4, 26.7, 25.7, 24.7, 22.9, 10.5, 10.4; IR (thin film): 3399, 2960, 2922, 2854,1659, 1626, 1603,1063, 1047, 753 cm^{-1}; HRMS (ESI-TOF): calculated for $C_{21}H_{27}O_2$ $[M+H]^+$ 311.2011, found 311.2012; $[\alpha]_D^{22}$ = + 39.9 (*c* 0.0075, $CHCl_3$). See Supplementary Figures 62 and 63 for ^{1}HNMR and ^{13}CNMR spectra.

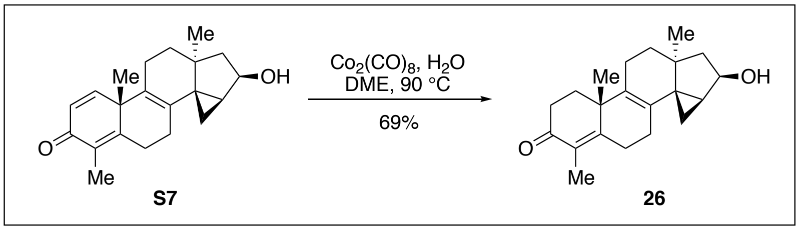

Supplementary Figure 97. Synthesis of Tetracycle **26**

Tetracycle 26: To a stirring solution of **S7** (2.9 g, 9.3 mmol, 1.0 equiv) and water (3.6 g, 200 mmol, 22 equiv) in 190 mL dimethoxyethane was added $Co_2(CO)_8$ (8.0 g, 23 mmol, 2.5 equiv). The resulting mixture was refluxed for 3 hr at 90 °C, and for 1 hr at 100 °C. The reaction mixture was cooled to rt, and another portion of $Co_2(CO)_8$ (3.2 g, 9.4 mmol, 1.0 equiv) was added. The reaction mixture was warmed to 90 °C again, continued to reflux for 1.7 hr, and then cooled to rt. The resulting mixture was passed through a pad of silica gel using 850 mL ethyl acetate, 100 mL 5% methanol/95% ethyl acetate, and 10% methanol/90% ethyl aceate as the eluent, and then the filtrate was concentrated in *vacuo*. The crude product was purified by dry column vacuum chromatography[1] using 200 mL dichloromethane and then a gradient elution (20% ethyl acetate/80% hexanes to 50% ethyl aceate/50% hexanes, 5% increase in ethyl acetate/ fraction, 500 mL/fraction) to afford 2.02 g of the title compound **26** as an amourphous white solid (69% yield). Spectral data for **26**: ^{1}H NMR (600 MHz, Chloroform-*d*) δ 4.63 (td, *J* = 7.7, 4.0 Hz, 1H), 2.72 (ddd, *J* = 12.9, 4.9, 2.3 Hz, 1H), 2.51 (ddd, *J* = 17.3, 14.7, 5.1 Hz, 1H), 2.42 (ddd, *J* = 17.3, 4.8, 2.7 Hz, 1H), 2.24 – 2.07 (m, 4H), 1.94 (dt, *J* = 7.4, 3.5 Hz, 1H), 1.86 – 1.66 (m, 7H), 1.65 – 1.58 (m, 3H), 1.30 (s, 3H), 0.96 (dd, *J* = 12.5, 8.7 Hz, 1H), 0.88 (s, 3H), 0.82 (dd, *J* = 5.1, 3.1 Hz, 1H), 0.38 (dd, *J* = 7.5, 5.2 Hz, 1H); ^{13}C NMR (150 MHz, Chloroform-*d*) δ 198.7, 163.5, 133.3, 129.5, 127.4, 73.7, 41.4, 40.0, 38.9, 37.7, 34.0, 33.4, 31.6, 26.33, 26.26, 26.1, 23.4, 23.0, 21.7, 10.8, 10.1; IR (thin film): 3399, 2922, 1660, 1613, 1450, 1373, 1357, 1332, 1074, 1054, 1025 cm^{-1} ;HRMS: calculated for $C_{21}H_{29}O_2$ $[M+H]^+$ 313.2168, found 313.2164; $[\alpha]_D^{22}$ = +252.2 (*c* 0.021, $CHCl_3$). See Supplementary Figures 64 and 65 for ^{1}HNMR and ^{13}CNMR spectra.

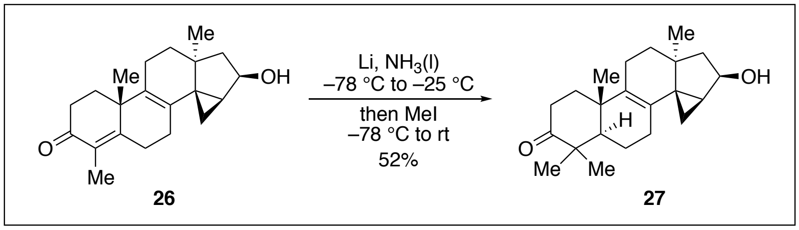

Supplementary Figure 98. Synthesis of Tetracycle **27**

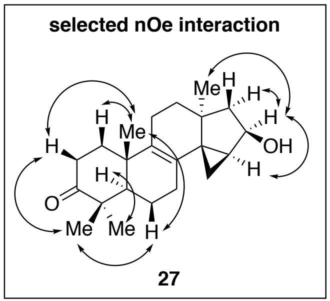

Supplementary Figure 99. Selected nOe interaction

Tetracycle 27: To a stirring blue solution of Li (20 mg, 2.9 mmol, 10 equiv) in 10 mL NH_3 (l) at –78 °C under N_2 atmosphere was added a solution of **26** (89 mg, 0.28 mmol, 1.0 equiv) in 2 mL THF dropwise. The resulting mixture was stirred for 15 min at –78 °C, warmed to –35 °C, and then stirred for 50 min while maintaining the cold bath temperature between –35 °C and –25 °C. After this period, the reaction mixture was cooled to –78 °C, and then MeI (0.81 g, 5.7 mmol, 20 equiv) was added dropwise. The cold bath was removed, and NH_3 (l) was evaporated under a stream of N_2 over 30 min. 5 mL saturated solution of NH_4Cl was added, and the resulting mixture was further diluted with 10 mL ethyl acetate and 5 mL water. The organic layer was separated, and the aqueous layer was extracted with 25 mL × 2 ethyl acetate. The combined organic layers were dried with Na_2SO_4, filtered, and the filtrate was concentrated in *vacuo*. SiO_2 flash column chromatography afforded the title compound **27** as a pale yellow film (48 mg, 52%); Spectral data for **27**: ^{1}H NMR (500 MHz, Chloroform-*d*) δ 4.69 – 4.59 (m, 1H), 2.57 (ddd, *J* = 15.9, 10.4, 7.5 Hz, 1H), 2.46 (ddd, *J* = 15.9, 7.5, 3.8 Hz, 1H), 2.20 – 2.07 (m, 2H), 2.07 – 2.00 (m, 1H), 1.94 (dt, *J* = 7.4, 3.5 Hz, 1H), 1.81 – 1.71 (m, 1H), 1.71 – 1.56 (m, 7H), 1.45 (dq, *J* = 18.4, 6.2 Hz, 1H), 1.36 (br s, 1H), 1.09 (s, 3H), 1.08 (s, 3H), 1.05 (s, 3H), 0.97 – 0.90 (m, 4H), 0.75 (dd, *J* = 5.0, 3.1 Hz, 1H), 0.32 (dd, *J* = 7.5, 5.0 Hz, 1H); ^{13}C NMR (150 MHz, Chloroform-*d*) δ 217.9, 134.8, 128.0, 73.9, 51.7, 47.4, 41.7, 39.1, 38.1, 37.5, 35.5, 34.6, 31.9, 26.79, 26.76, 26.3, 22.9, 21.9, 21.3, 19.9, 19.7, 10.2; IR (thin film): 3392, 2950, 2924, 1704, 1473, 1457, 1383, 1058, 1020, 754 cm^{-1}; HRMS: calculated for C22H31O2 $[M–H]^+$ 327.2324, found 327.2325; $[\alpha]_D^{22}$ = +82.0 (*c* 0.0065, $CHCl_3$). See Supplementary Figures 66 and 67 for ^{1}HNMR and ^{13}CNMR spectra.

Supplementary Notes

Supplementary Note 1: At room temperature, (*S*)-BINOL or (*R*)-BINOL was fully dissolved in dichloromethane. The suspension was observed when the solution was cooled to –78 °C.

Supplementary Note 2: When the crude **28** was used, 54% yield was obtained over the two steps. The following procedure was used: To a stirring solution of **11** (0.23 g, 0.77 mmol, 1 equiv) in 7 mL anhydrous toluene at rt under N_2 atmosphere was added DIBAL-H (1.0 M in hexanes, 7.7 mL, 7.7 mmol, 10 equiv). The resulting mixture was warmed to 100 °C, refluxed overnight (approx. 20 hr), and then cooled to rt. Small chunks of ice was slowly added, and the resulting mixture was acidified with 3M aqueous hydrochloric acid. The organic layer was separated, and the aqueous layer was extracted with 50 mL × 3 ethyl acetate. The combined organic layers were dried over Na_2SO_4, filtered, and then the filtrate was concentrated in *vacuo* to afford 0.21 g of the crude **28** as an amorphous yellow solid. The crude **28** (0.21 g) at 0 °C under N_2 atmosphere was added 7 mL HFIP, and stirred for 18 min. PIDA (0.21 g, 0.65 mmol) was added to the reaction mixture, stirred for 1 min at the same temperature (PIDA was fully dissolved at this point), and then 2 mL saturated sodium bicarbonate solution was added. The resulting mixture was further diluted with 50 mL ethyl acetate, and the organic layer was separated. The aqueous layer was extracted with 50 mL ethyl acetate, and then the combined organic layers were concentrated in *vacuo*. The crude product was purified with SiO_2 flash column chromatography to afford 0.12 g of the title compound **20** as an amorphous white solid (54% isolated yield).

Caution: Prolonged stirring after PIDA addition resulted in a significantly lower yield.

Supplementary Note 3: The absolute stereochemistry of this molecule was confirmed by X-ray Crystallography (deposition number: CCDC 1897943).

Supplementary References

1. Daniel Sejer Pedersen, C. R. Dry Column Vacuum Chromatography. *Synthesis*, 2431-2434 (2001).
2. Reddy, B. V. S., Borkar, P., Yadav, J. S., Sridhar, B. & Grée, R. Tandem Prins/Friedel–Crafts Cyclization for Stereoselective Synthesis of Heterotricyclic Systems. *The Journal of Organic Chemistry* **76**, 7677-7690 (2011).
3. Dai, M., Krauss, I. J. & Danishefsky, S. J. Total Synthesis of Spirotenuipesines A and B. *The Journal of Organic Chemistry* **73**, 9576-9583 (2008).
4. Deng, H., Cao, W., Liu, R., Zhang, Y. & Liu, B. Asymmetric Total Synthesis of Hispidanin A. *Angewandte Chemie International Edition* **56**, 5849-5852 (2017).
5. Turner, H. M., Patel, J., Niljianskul, N. & Chong, J. M. Binaphthol-Catalyzed Asymmetric Conjugate Arylboration of Enones. *Organic Letters* **13**, 5796-5799 (2011).
