# Supplementary material for: A synthesis strategy for tetracyclic terpenoids leads to agonists of ERβ
Source: Nat Commun. 2019 Jun 4;10:2448. doi: 10.1038/s41467-019-10415-6 (PMC6547701; doi:10.1038/s41467-019-10415-6)
Supplement: Supplementary file 2 — Reporting Summary [file 41467_2019_10415_MOESM2_ESM.pdf]

nature research

Corresponding author(s): Glenn C. Micalizio and Arti B. Gaur

Reporting Summary

Nature Research wishes to improve the reproducibility of the work that we publish. This form provides structure for consistency and transparency in reporting. For further information on Nature Research policies, see Authors & Referees and the Editorial Policy Checklist.

Statistical parameters

When statistical analyses are reported, confirm that the following items are present in the relevant location (e.g. figure legend, table legend, main text, or Methods section).

n/a	Confirmed	
☐	☒	The exact sample size (*n*) for each experimental group/condition, given as a discrete number and unit of measurement
☐	☒	An indication of whether measurements were taken from distinct samples or whether the same sample was measured repeatedly
☒	☐	The statistical test(s) used AND whether they are one- or two-sided<br>*Only common tests should be described solely by name; describe more complex techniques in the Methods section.*
☐	☒	A description of all covariates tested
☒	☐	A description of any assumptions or corrections, such as tests of normality and adjustment for multiple comparisons
☐	☒	A full description of the statistics including central tendency (e.g. means) or other basic estimates (e.g. regression coefficient) AND variation (e.g. standard deviation) or associated estimates of uncertainty (e.g. confidence intervals)
☒	☐	For null hypothesis testing, the test statistic (e.g. *F*, *t*, *r*) with confidence intervals, effect sizes, degrees of freedom and *P* value noted<br>*Give P values as exact values whenever suitable.*
☒	☐	For Bayesian analysis, information on the choice of priors and Markov chain Monte Carlo settings
☒	☐	For hierarchical and complex designs, identification of the appropriate level for tests and full reporting of outcomes
☒	☐	Estimates of effect sizes (e.g. Cohen's *d*, Pearson's *r*), indicating how they were calculated
☒	☐	Clearly defined error bars<br>*State explicitly what error bars represent (e.g. SD, SE, CI)*

Our web collection on statistics for biologists may be useful.

Software and code

Policy information about availability of computer code

Data collection	IncuCyte ZOOM 2016B Firmware 20162.1.1.0 was used for generating and exporting images from the IncuCyte Live-Cell Analysis System
Data analysis	GraphPad Prism 6.01 (La Jolla, CA) was used for generating figures. FlowJo 10.5.3 (TreeStar, Ashland, OR) was used for Flow Cytometry Analysis

For manuscripts utilizing custom algorithms or software that are central to the research but not yet described in published literature, software must be made available to editors/reviewers upon request. We strongly encourage code deposition in a community repository (e.g. GitHub). See the Nature Research guidelines for submitting code & software for further information.

Data

Policy information about availability of data

All manuscripts must include a data availability statement. This statement should provide the following information, where applicable:

- Accession codes, unique identifiers, or web links for publicly available datasets
- A list of figures that have associated raw data
- A description of any restrictions on data availability

A Data Availability Statement is included in the manuscript, and all raw data has been provided in the requested Source Data File

Field-specific reporting

Please select the best fit for your research. If you are not sure, read the appropriate sections before making your selection.

☒ Life sciences ☐ Behavioural & social sciences ☐ Ecological, evolutionary & environmental sciences

For a reference copy of the document with all sections, see nature.com/authors/policies/ReportingSummary-flat.pdf

Life sciences study design

All studies must disclose on these points even when the disclosure is negative.

Sample size	All cell culture data in this manuscript (looking at the effect of tetracyclic terpenoids 28 and ent-28 on human glioma cell lines, human neural stem cells as well as human astrocytes) are representative of four independent experiments and four replicates of each treatment condition were analyzed per experiment. Sample size was chosen taking into consideration the means of the target values between the experimental groups (28 or ent-28) and the control group (untreated or equivalent amount of solvent; DMSO), the standard error and the statistical analysis used. Sample size was defined on the basis of past experience with human glioma cell lines, human neural stem cells as well as human astrocytes to detect differences of 20% or greater between the groups (10% significance level and 80% power).
Data exclusions	No Data were excluded
Replication	For each experiment the number of biologically independent samples is reported in the figure legend.
Randomization	This work looked at the effect of tetracyclic terpenoids (28 and ent-28) on human glioma cell lines, human neural stem cells as well as human astrocytes. Neither the compounds nor the cells were randomised in the experiments. Each compound was tested in a dose/time dependent manner with each cell type. Untreated cells as well as cells treated with the equivalent amount of solvent (DMSO) were used as controls.
Blinding	For the hemocytometer analyses, the investigators were unaware of the experimental groups.

Reporting for specific materials, systems and methods

Materials & experimental systems

n/a	Involved in the study	
☒	☐	Unique biological materials
☐	☒	Antibodies
☐	☒	Eukaryotic cell lines
☒	☐	Palaeontology
☒	☐	Animals and other organisms
☒	☐	Human research participants

Methods

n/a	Involved in the study	
☒	☐	ChIP-seq
☐	☒	Flow cytometry
☒	☐	MRI-based neuroimaging

Antibodies

Antibodies used	FITC-Annexin-V (BioLegend Cat# 640906, Lot# B266195), 7-AAD (Biolegend Cat# 420403, Lot # B251165)
Validation	Antibodies were used in accordance to manufacturer's protocols. Lot-specific certificates of analysis can be found on the manufacturer's website.

Eukaryotic cell lines

Policy information about cell lines

Cell line source(s)	U87 and U251 glioma cell lines were purchased from ATCC with documentation of identity.
Authentication	U87 and U251 glioma cell lines were purchased from ATCC with documentation of identity. Newly obtained frozen vials of cells were thawed, morphology was verified and cells were checked for possible mycoplasma contamination. Cell lines were expanded (3 passages) and cryo-aliquoted for future studies. In vivo glioma xenograft growth from these glioma cell lines was verified in immune-compromised mice following cell injection. Low-passage stocks were used in this study. Multiple clones were routinely tested as described above to minimize the risks of off-target effects or selection of an unrepresentative sub-population.

Mycoplasma contamination	Cell lines were negative for Mycoplasma before starting experiments using MycoSEQ™ Mycoplasma Detection Kit, with Discriminatory Positive Control (ThermoFisher; Catalog number: 4460623). These cell lines have been and are routinely checked for mycoplasma as well as genotyped.
Commonly misidentified lines (See ICLAC register)	N/A

Flow Cytometry

Plots

Confirm that:

☒ The axis labels state the marker and fluorochrome used (e.g. CD4-FITC).

☒ The axis scales are clearly visible. Include numbers along axes only for bottom left plot of group (a 'group' is an analysis of identical markers).

☒ All plots are contour plots with outliers or pseudocolor plots.

☒ A numerical value for number of cells or percentage (with statistics) is provided.

Methodology

Sample preparation	As mentioned previously in the Materials and Methods section, U251 and U87 cells were seeded in six-well plates (300,000 cells/well) 24 hours prior to treatment with 28, ent-28, TMZ, or vehicle control DMSO for 24 hours. After cells were treated for 24 hours, media was aspirated and cells were washed with PBS. Cells were trypsinized and spun down with media at 1200 rcf. Supernatant was aspirated, leaving the cell pellet. Cell pellet was resuspended in Annexin V Binding Buffer (Cat# 422201, Lot# B179321) and stained with FITC-Annexin-V and incubated for 15 minutes at 4 degrees C in the dark. Samples were subsequently treated with 7-AAD and incubated for 15 minutes at 4 degrees C in the dark. Prepared samples were analyzed using the MACSQuant Analyzer 10.
Instrument	MACSQuant Analyzer 10 (Miltenyi Biotec)
Software	Software for collection was done with MACSQuantify Version 2.11. Analysis was done with FlowJo 10.5.3 (TreeStar, Ashland, OR)
Cell population abundance	Cells were not sorted. All cells were included in the FACS. Cell clumps were excluded for singlets.
Gating strategy	As mentioned in the supplementary figure, FSC/SSC gating was performed on FSC-A/FSC-H plots to exclude doublets. U251 and U87 cells treated with staurosporine as positive controls were stained for Annexin V-FITC and 7-AAD. Gating was performed using the resulting contour plots.

☒ Tick this box to confirm that a figure exemplifying the gating strategy is provided in the Supplementary Information.
